# Nobiletin (NOB) nanoparticles ameliorate chronic paradoxical sleep deprivation (PSD)-induced cognitive deficits in rats

**DOI:** 10.1038/s41420-025-02738-9

**Published:** 2025-10-13

**Authors:** Yujie Hu, Dan Hou, Shuling Wang, Nianzhen Wang, Qiang Wang, Congcong Zhang, Junling Chen, Xiaohui Su, Guoshuai Yang

**Affiliations:** Department of Neurology, Haikou Affiliated Hospital of Central South University Xiangya School of Medicine, Haikou, China

**Keywords:** Drug delivery, Sleep disorders

## Abstract

Nobiletin (NOB), a naturally occurring polymethoxyflavonoid, has been shown to regulate the expression of the clock gene BMAL1. This study aims to explore the impact of NOB nanoparticles on microglial polarization and cognitive impairments resulting from chronic sleep paradoxical deprivation (PSD), as well as the mechanisms involved. Following PSD modeling, rats treated with NOB nanoparticles exhibited significantly improved cognitive performance in behavioral tests. The treatment upregulated the expression of BMAL1, SIRT1, E2F1, and the NAD+/NADH ratio, shifted microglial polarization from the pro-inflammatory M1 phenotype to the anti-inflammatory M2 phenotype, and enhanced antioxidant defenses. The NAD^+^ inhibitor apocynin and silencing of BMAL1 could reverse the effects of NOB nanoparticles. Overexpression of BMAL1 had similar effects to NOB nanoparticles in LPS-induced cellular models, while silencing of SIRT1 or E2F1 reversed the effects. Co-immunoprecipitation experiments illustrated the binding of SIRT1 and BMAL1 and SIRT1 and E2F1. NOB nanoparticles alleviate chronic PSD-induced microglia M1 polarisation, inflammation, and cognitive deficits in rats by a mechanism that may be related to the BMAL1/SIRT1/E2F1 axis, providing a new direction for the therapeutic approach of chronic PSD-associated cognitive deficits.

**Figure:**
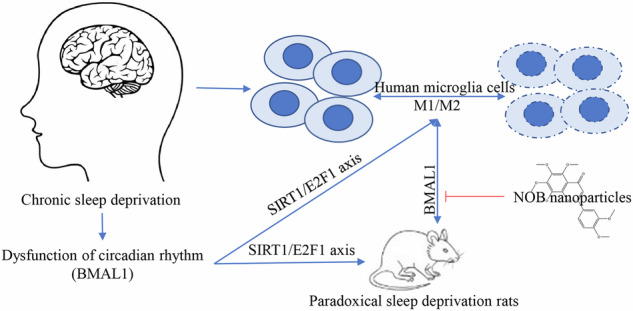
NOB nanoparticles alleviate chronic PSD-induced microglia M1 polarisation, inflammation, and cognitive deficits in rats by a mechanism that may be related to the BMAL1/SIRT1/E2F1 axis.

NOB nanoparticles alleviate chronic PSD-induced microglia M1 polarisation, inflammation, and cognitive deficits in rats by a mechanism that may be related to the BMAL1/SIRT1/E2F1 axis.

## Introduction

Sleep deprivation, significantly influenced by economic elements within contemporary society, has evolved into a worldwide concern [1]. Predominantly, lifestyle habits or sleep disorders account for the majority of people experiencing sleep deprivation. Chronic sleep deprivation (CSD), one prevalent aspect of sleep disorders, merits attention [2]. CSD is notable for its role in elevating plasma concentrations of inflammatory cytokines and is widely recognized as a contributing risk factor for developing anxiety disorders and major depressive disorder [3]. However, the specific mechanisms by which CSD induces cognitive deficits remain shrouded in mystery.

Nobiletin (NOB), a dietary compound naturally occurring in certain polymethoxy flavonoids, has been shown to have a protective effect in various neuroinflammatory conditions [4, 5]. For example, NOB can act as a clock regulator to alleviate neuroinflammation and cognitive impairment associated with astroglial proliferation in an Alzheimer’s disease model [4]. NOB has neuroprotective and anti-dementia potential, partly attributed to its antioxidant, anti-nitration, and anti-inflammatory properties [5]. NOB can improve neurogenesis by alleviating neuroinflammation in the hippocampus of D-galactose-induced aging mice, thus restoring memory impairment [6]. Nanoparticles have been widely reported in inflammation-related diseases [7, 8]. For instance, nanoparticle-mediated drug delivery can target and inhibit tumor inflammation to resist metastatic tumors [8]. NOB and its derivatives, being naturally occurring, non-toxic, and capable of improving metabolism, show considerable potential as drug candidates [9]. However, NOB’s practical application is limited due to its hydrophobic and crystalline nature, which renders it challenging to process, store, and absorb [10]. Nanomaterials or materials with at least one dimension ranging from 1 to 100 nm in a three-dimensional space can be an effective solution [7]. Utilizing nanoparticles can considerably enhance NOB’s bioavailability in animal models [11], either through assembled nanoparticles composed of NOB, zein, and tannic acid (TA) [12] or NOB nanoparticles encapsulated with tannic acid and trivalent metal ions (Fe^3+^ or Al^3+^) films [13]. Applied in the form of amorphous solid dispersion nanoparticles, the bioavailability and central nervous system delivery of NOB can markedly improve [14]. However, the explicit role of NOB nanoparticles in a chronic PSD-induced rat model is yet to be determined.

The brain and muscle arnt-like protein 1 (BMAL1) plays a crucial role in the progression of circadian rhythm disorders and sleep syndromes, as it is a pivotal circadian gene [15, 16]. When BMAL1 is silenced, it triggers an upswing in transcriptional programs linked to inflammation and stress responses [17]. Additionally, BMAL1’s transcriptional network shows connections to human sleep deprivation, major depressive disorder, and aging [18]. Thus, unravelling the mechanisms governing BMAL1-related circadian rhythm regulation could illuminate novel treatment pathways for CSD and various mental disorders. Nobiletin (NOB) is known for its positive influence on the biological clock’s maintenance [19]. Administering NOB in a dose-dependent method can counteract the reduction of BMAL1 and retinoic-acid receptor-related orphan receptors (RORs) expression in the brain and muscle prompted by laparotomy [20]. This interaction subsequently suppresses inflammation, thus providing relief from postoperative cognitive dysfunction [20]. In addition to this, phenotypic screening studies identified NOB as an RORs agonist capable of enhancing circadian rhythm amplitudes. It effectively escalates the expression of clock and clock-controlled genes in diabetic or obese mouse models, facilitating blood glucose and lipid equilibrium, thus preserving metabolic function [21]. However, despite these findings, the role of NOB nanoparticles on BMAL1 in chronic paradoxical sleep deprivation (PSD) and the underlying mechanisms remain to be fully understood.

Microglial cells, crucial immune cells within the nervous system, operate as tissue-specific macrophages. They play a pivotal role in brain development, ensuring neural environment sustainability and facilitating responses to and repair of injuries [22]. The role of BMAL1 in glioblastoma stem cells is crucial for immunosuppression in glioblastoma conditions [23]. Furthermore, the expression of BMAL1 is linked to the M2 polarization of microglial cells [24]. The enhancement of melatonin expression contributes to heightened levels of BMAL1 and CD206, which decreases oxidative stress and alleviates cognitive impairments associated with cortical spreading depression [25]. An interesting observation is the role of the small molecule antagonist SR8278, which has been shown to increase the uptake of Aβ1-42 in microglial cells and amplify BMAL1 transcription, leading to the polarization of these cells towards an M2 phenotype [26]. Hence, it can be deduced that the BMAL1 may potentially play a significant role in the polarization of microglial cells. However, the exact influence of this pathway on chronic PSD-induced cognitive impairment in rats needs further investigation.

Therefore, the aim of this study was to investigate the role and mechanism of NOB nanoparticles on BMAL1 in chronic PSD-induced cognitive impairment in rats, with a view to providing new insights into cognitive-behavioral therapies for chronic PSD-induced cognitive impairment in rats.

## Results

### NOB nanoparticles affect LPS-induced microglia inflammation and BMAL1 expression

**Fig. 1:**
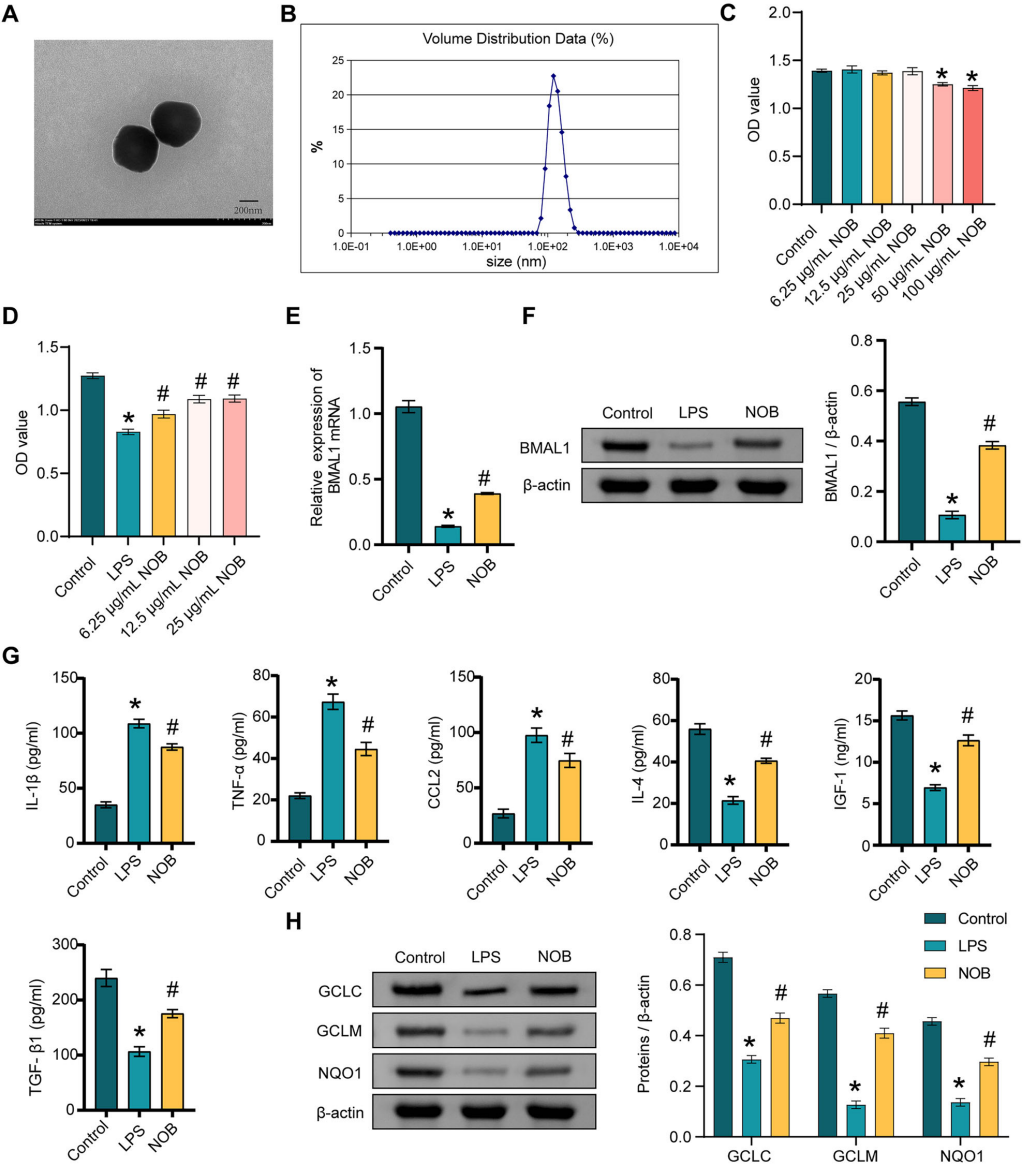
NOB nanoparticles affect LPS-induced microglia inflammation and BMAL1 expression. **A** The morphology of NOB nanoparticles was shown via TEM. **B** The diameter of NOB nanoparticles was evaluated by NTA. **C** The effects of different concentrations of NOB nanoparticles on HMC3 cell viability were assessed by CCK8. **D** The effects of different concentrations of NOB nanoparticles on 100 ng/mL LPS-induced HMC3 cells were assessed by CCK8. **E**, **F** BMAL1 expression was detected by qRT-PCR and western blot. **G** The levels of IL-1β, TNF-α, CCL2, IL-4, IGF-1, and TGF-β1 were detected by ELISA. **H** The expression levels of GCLC, GCLM, and NQO1 were detected by western blot. ^*^P < 0.05, vs the Control; ^#^*P* < 0.05, vs the LPS. *n* = 3/group.

First, NOB nanoparticles were prepared. Figure 1A shows transmission electron microscopy (TEM) image of NOB nanoparticles. The average particle size of NOB nanoparticles was about 161.4 nm (Fig. 1B). Next, different concentrations (6.25, 12.5, 25, 50, 100 μg/mL) of NOB nanoparticles were used to treat HMC3 cells. The results showed that NOB nanoparticles (6.25, 12.5, and 25 μg/mL) had no significant effect on HMC3 cell viability, whereas NOB nanoparticles (50 and 100 μg/mL) inhibited the viability of HMC3 cells (Fig. 1C). Treatment with NOB nanoparticles (6.25, 12.5, and 25 μg/mL) significantly reversed the LPS-induced decrease in cell viability, and the concentrations of 12.5 and 25 μg/mL were the most effective. NOB nanoparticles at concentrations of 12.5 and 25 μg/mL had similar effects on LPS-induced cell viability. (Fig. 1D). Therefore, the concentration of 12.5 μg/mL was chosen for the follow-up study. The expression of BMAL1 was significantly increased in the NOB group compared to the LPS group (Fig. 1E, F). NOB nanoparticle treatment reversed the LPS-induced increase in IL-1β, CCL2, and TNF-α levels and the decrease in IL-4, IGF-1, and TGF-β1 levels (Fig. 1G). In addition, NOB nanoparticles reversed the LPS-induced decrease in GCLC, GCLM, and NQO1 expression (Fig. 1H). The above results reveal that NOB nanoparticles alleviate LPS-induced inflammation and BMAL1 expression in microglia.

### NOB nanoparticles affect cognitive deficits, the BMAL1/SIRT1/E2F1 axis and microglia polarization in chronic PSD-induced rats

**Fig. 2:**
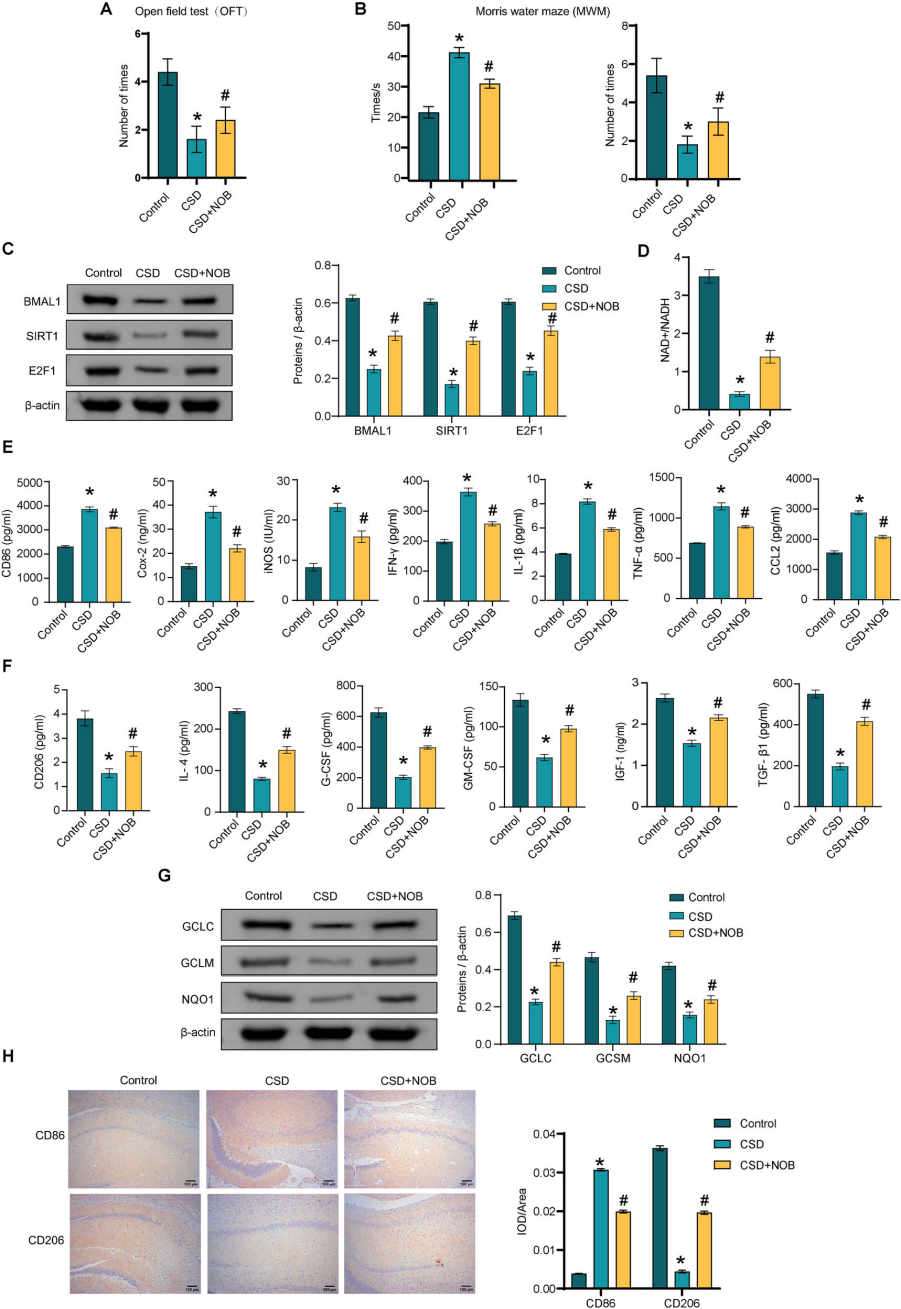
NOB nanoparticles affect cognitive deficits, the BMAL1/SIRT1/E2F1 axis and microglia polarization in chronic PSD-induced rats. **A** The OFT was utilized to record the frequency of entries made by the rats into the central zone. *n* = 6. **B** The time to find the platform (escape latency) and the number of times the rat crossed the original platform within 2 min were counted by MWM. *n* = 6. **C** The expression levels of BMAL1, SIRT1 and E2F1 were detected by western blot. *n* = 3. **D** The NAD^+^/NADH levels were assessed by an NAD+/NADH assay kit. *n* = 3. **E** The levels of M1 microglia markers CD86, Cox-2, iNOS, IFN-γ, IL-1β, TNF-α, and CCL2 were detected by ELISA. *n* = 3. **F** The levels of M2 microglia markers CD206, IL-4, G-CSF, GM-CSF, IGF-1, and TGF-β1 were assessed by ELISA. *n* = 3. **G** The expressions of GCLC, GCLM and NQO1 were detected in hippocampal tissues by western blot. *n* = 3. **H** The expressions of CD86 and CD206 were assessed by IHC in hippocampal tissue. *n* = 3. ^*^P < 0.05, vs the control; ^#^*P* < 0.05, vs the LPS.

Next, NOB nanoparticles were injected intravenously into the animals to explore their effects on the animal model. Treatment with NOB nanoparticles resulted in an increased number of rats in the model group successfully entering the central zone and crossing the platform within 2 min. Additionally, it decreased the time it took the model rats to locate the platform (Fig. 2A, B). SIRT1 is a NAD^+^-dependent protein deacetylase that is required for high-amplitude circadian transcription of several core clock genes [27]. SIRT1/BMAL1 activation ameliorates cerebral ischemia-reperfusion injury in diabetic mice [28]. SIRT1 binds to E2F1 and affects neonatal brain injury [29]. Therefore, the effect of NOB nanoparticle treatment on SIRT1, E2F1, and NAD^+^/NADH levels was explored. The levels of BMAL1, SIRT1, E2F1, and NAD^+^/NADH were in hippocampal tissue higher in the Model+NOB group than in the Model group (Fig. 2C, D). NOB nanoparticle treatment reduced the chronic PSD-induced rise in M1-type microglial cell makers, including CD86, Cox-2, iNOS, IFN-γ, IL-1β, TNF-α, and CCL2 levels (Fig. 2E, H). NOB nanoparticle treatment reversed the chronic PSD-induced decrease in M2-type microglia makers, including CD206, IL-4, G-CSF, GM-CSF, IGF-1, and TGF-β1 levels (Fig. 2F, H). In addition, NOB nanoparticles reversed the chronic PSD-induced decrease in GCLC, GCLM, and NQO1 expression (Fig. 2G). The above results suggest that NOB nanoparticles ameliorate cognitive deficits in model rats and affect the BMAL1/SIRT1/E2F1 axis and microglia polarization.

### NAD^+^ metabolism is involved in the regulation of NOB nanoparticles-mediated inflammation, oxidative stress, and BMAL1/SIRT1/E2F1 pathway in the presence of LPS

**Fig. 3:**
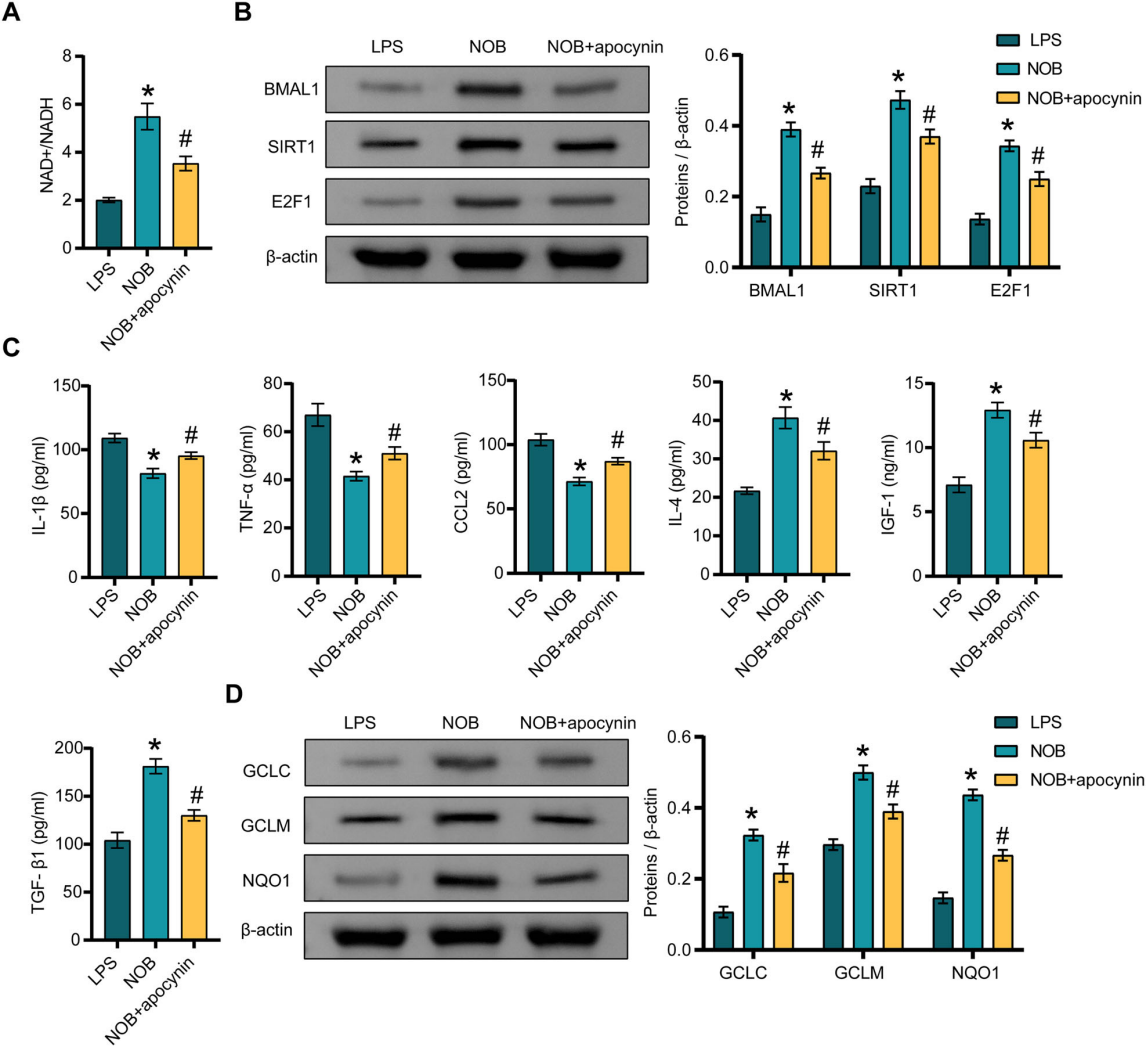
NAD+ metabolism is involved in the regulation of NOB nanoparticles-mediated inflammation, oxidative stress, and BMAL1/SIRT1/E2F1 pathway in the presence of LPS. **A** The NAD^+^/NADH levels were assessed by the NAD+/NADH assay kit. **B** The expressions of BMAL1, SIRT1, and E2F1 were detected by western blot. **C** The levels of IL-1β, TNF-α, CCL2, IL-4, IGF-1, and TGF-β1 were detected by ELISA. **D** The expressions of GCLC, GCLM, and NQO1 were detected by western blot. ^*^P < 0.05, vs the LPS; ^#^*P* < 0.05, vs the NOB. *n* = 3/group.

To further investigate whether NAD^+^ metabolism mediates the effects of NOB nanoparticles on LPS-induced inflammation and oxidative stress, we treated cells with the NAD^+^ inhibitor apocynin. The results showed that apocynin inhibited NOB nanoparticle-induced elevation of NAD^+^/NADH levels in the presence of LPS (Fig. 3A). Apocynin treatment significantly reversed the increase in the levels of BMAL1, SIRT1, E2F1, IL-4, IGF-1, TGF-β1, GCLC, GCLM, and NQO1 and the decrease in the levels of IL-1β, CCL2, and TNF-α in the cells of the model group after treatment with NOB nanoparticles (Fig. 3B–D), suggesting that NAD^+^ metabolism mediated the effects of NOB nanoparticles on LPS-induced microglia M1 polarization and the BMAL1/SIRT1/E2F1 axis.

### Silencing of BMAL1 inhibits the effect of NOB nanoparticles on LPS-induced microglia M1 polarization and the SIRT1/E2F1 axis

**Fig. 4:**
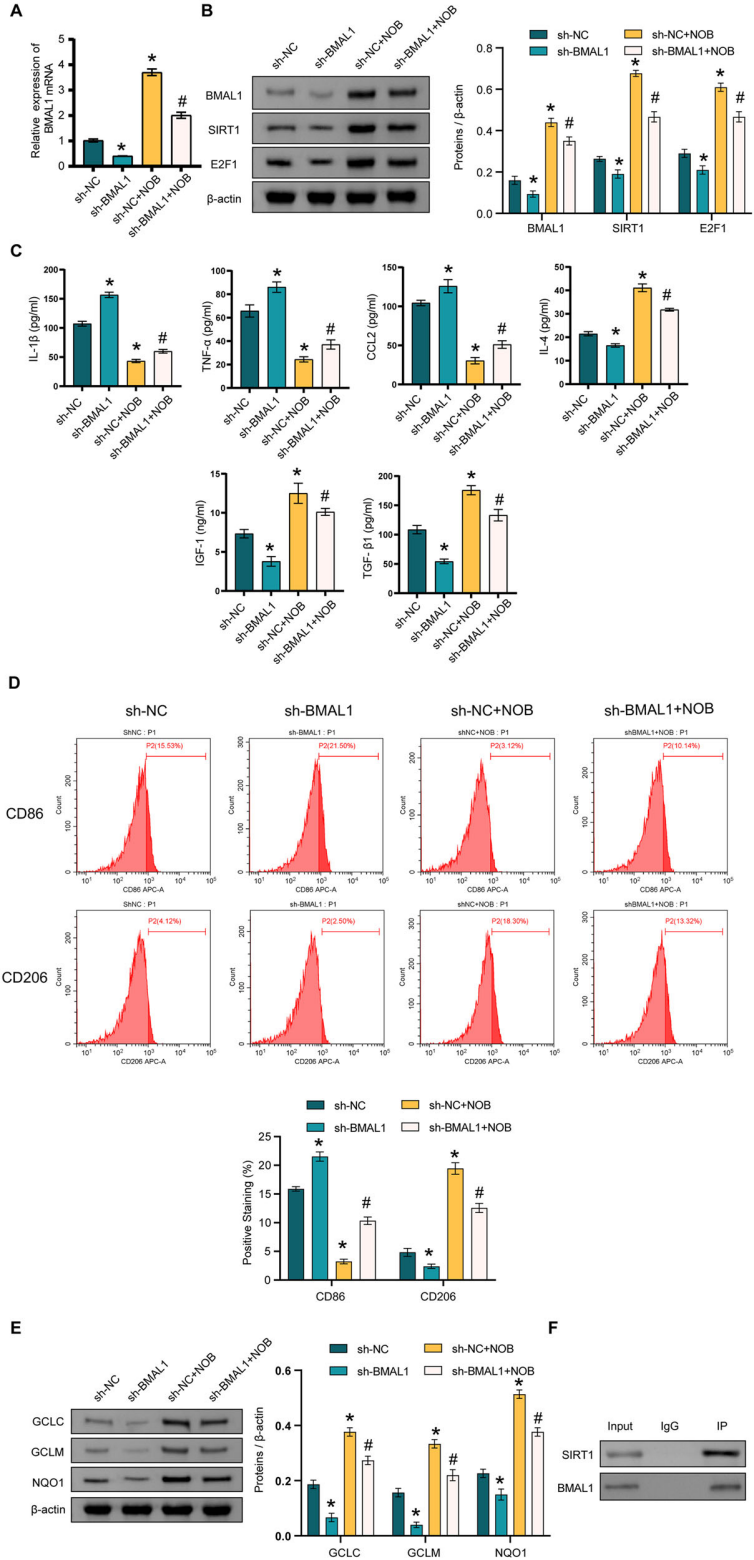
Silencing of BMAL1 inhibits the effect of NOB nanoparticles on LPS-induced microglia M1 polarization and SIRT1/E2F1 axis. **A** The BMAL1 expression was detected by qRT-PCR. **B** The BMAL1, SIRT1, and E2F1 expressions were detected by western blot. **C** The levels of IL-1β, TNF-α, CCL2, IL-4, IGF-1, and TGF-β1 were detected by ELISA. **D** The CD86 and CD206 expressions were detected by FCM. **E** The GCLC, GCLM, and NQO1 expressions were detected by western blot. **F** The interaction of SIRT1 and BMAL1 was assessed by coIP. ^*^P < 0.05, vs the sh-NC; ^#^*P* < 0.05, vs the sh-NC + NOB. *n* = 3/group.

To further investigate the effect of BMAL1 on LPS-induced microglia M1 polarization by NOB nanoparticles, we transfected the sh-BMAL1 lentiviral. sh-BMAL1 treatment significantly inhibited the expression of BMAL1 in cells of the model group. However, NOB nanoparticles promoted BMAL1 expression (Fig. 4A). The expression of BMAL1, SIRT1, and E2F1 was significantly decreased in the sh-BMAL1 + NOB group compared with that in the sh-NC + NOB group (Fig. 4B). sh-BMAL1 + NOB group exhibited lower levels of IL-4, IGF-1, TGF-β1, and CD206 than that in the sh-NC + NOB group; whereas sh-BMAL1 + NOB group showed higher levels of IL-1β, CCL2, TNF-α, and CD86 than that in the sh-NC + NOB group (Fig. 4C, D). The expressions of GCLC, GCLM, and NQO1 were significantly decreased in the sh-BMAL1 + NOB group compared with that in the sh-NC + NOB group (Fig. 4E). BMAL1 protein could bind to SIRT1 protein (Fig. 4F). In a word, silencing BMAL1 inhibits the effect of NOB nanoparticles on LPS-induced M1 polarization and SIRT1/E2F1 signaling in microglia.

### BMAL1 mediated LPS-induced microglial M1/M2 polarization via the SIRT1/E2F1 axis

**Fig. 5:**
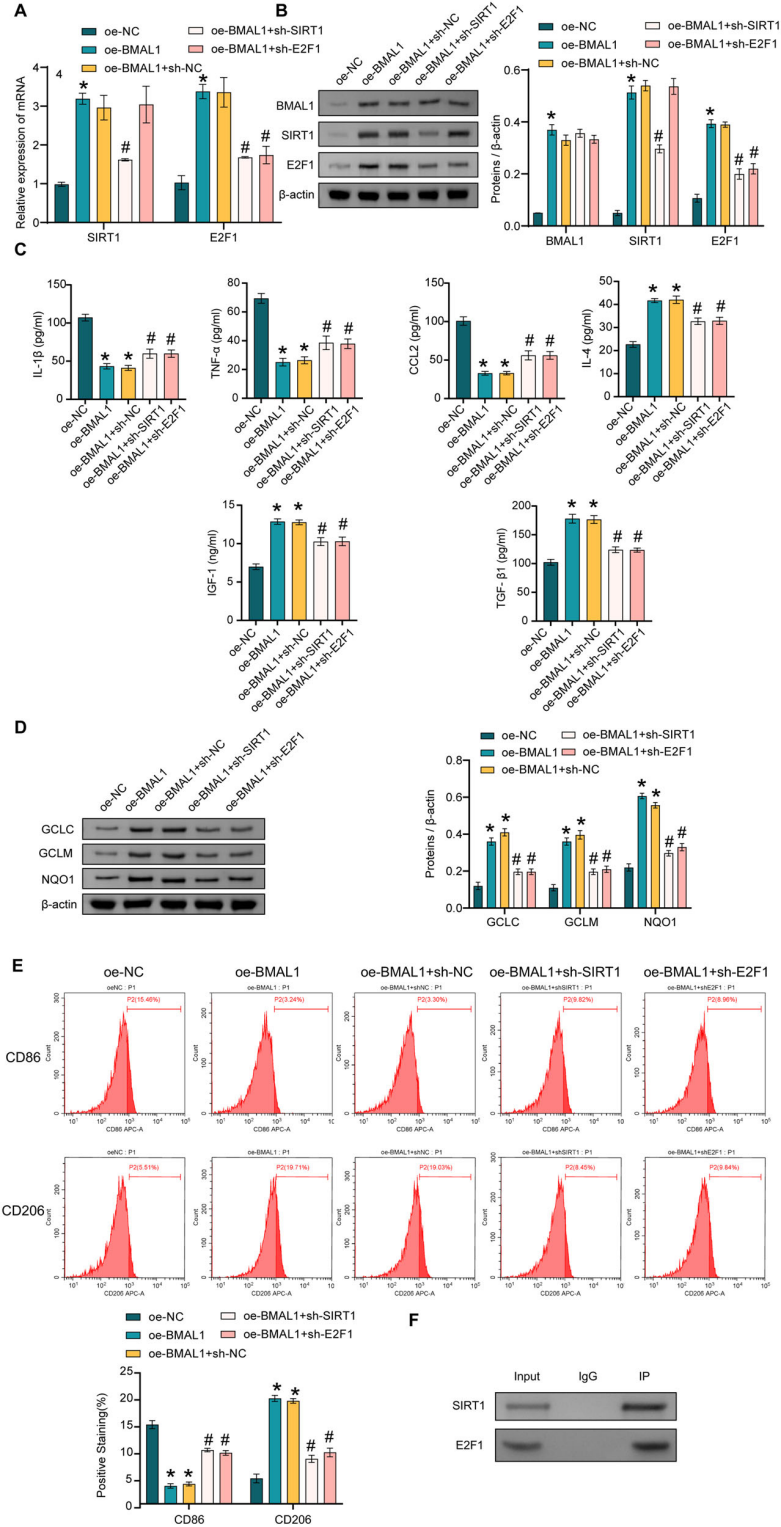
BMAL1 mediated LPS-induced microglial M1/M2 polarization via the SIRT1/E2F1 axis. **A** The expressions of SIRT1 and E2F1 were detected by qRT-PCR. **B** The BMAL1, SIRT1, and E2F1 expressions were detected by western blot. **C** The levels of IL-1β, TNF-α, CCL2, IL-4, IGF-1, and TGF-β1 were detected by ELISA. **D** The GCLC, GCLM, and NQO1 expressions were detected by western blot. **E** The CD86 and CD206 expressions were detected by FCM. **F** The interaction of SIRT1 and E2F1 was assessed by coIP. ^*^P < 0.05, vs the oe-NC; ^#^*P* < 0.05, vs the oe-BMAL1 + sh-NC. *n* = 3/group.

Next, we explored the role of the BMAL1/SIRT1/E2F1 axis on LPS-induced M1 polarization in microglia. The expressions of BMAL1, SIRT1, and E2F1 were significantly increased in the oe-BMAL1 group compared to the oe-NC group. When comparing the oe-BMAL1+sh-NC group with the oe-BMAL1+sh-SIRT1 group, a significant decrease in the expression of SIRT1 and E2F1 was observed. However, the expression levels of BMAL1 did not exhibit any notable difference. E2F1 expression was lower in the oe-BMAL1+sh-E2F1 group than in the oe-BMAL1+sh-NC group; however, BMAL1 and SIRT1 expression was not significantly different between these two groups (Fig. 5A, B). oe-BMAL1 group had higher levels of IL-4, IGF-1, and TGF-β1 compared to the oe-NC group, while oe-BMAL1 group had lower levels of IL-1β, CCL2, and TNF-α than that in the oe-NC group (Fig. 5C). The expressions of GCLC, GCLM, and NQO1 were significantly increased in the oe-BMAL1 group compared with the oe-NC group (Fig. 5D). oe-BMAL1 promoted the expressions of CD206 and inhibited the expressions of CD86 (Fig. 5E). There was an interaction between SIRT1 and E2F1 proteins (Fig. 5F). sh-SIRT1 and sh-E2F1 reversed the above results in oe-BMAL1+sh-NC group (Fig. 5A–F). The above results revealed that overexpression of BMAL1 could mediate LPS-induced microglia polarization through activation of the SIRT1/E2F1 axis.

### NOB nanoparticles ameliorate chronic PSD-induced cognitive deficits and microglia polarization in rats through activation of SIRT1

**Fig. 6:**
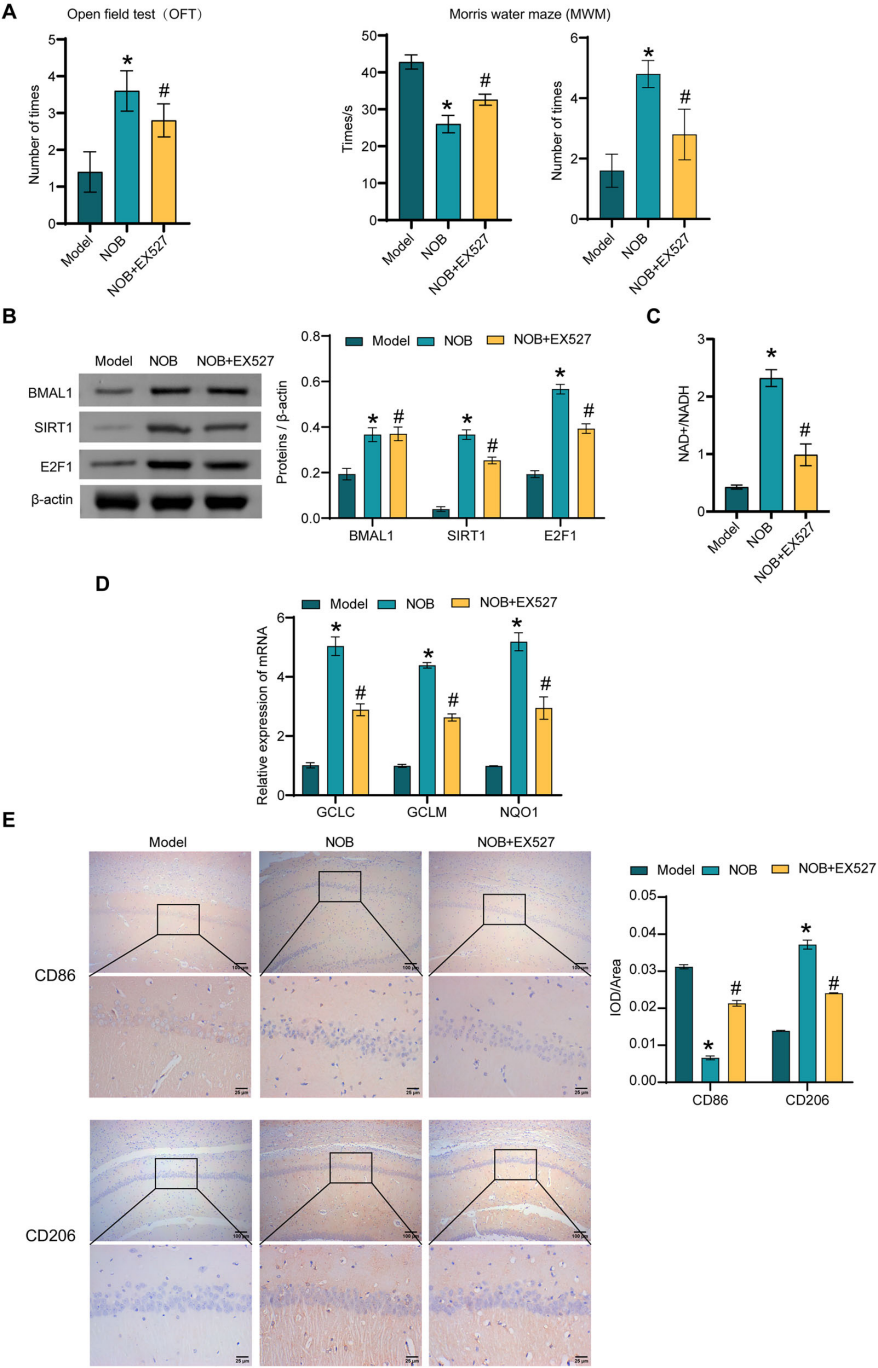
NOB nanoparticles ameliorate chronic PSD-induced cognitive deficits and microglia polarization in rats through activation of SIRT1. **A** The number of times the rats entered the central zone was recorded by the OFT. The time to find the platform (escape latency) and the number of times the rat crossed the original platform within 2 min were counted by the MWM. *n* = 6. **B** The expression levels of BMAL1, SIRT1 and E2F1 were detected by western blot. *n* = 3. **C** The NAD^+^/NADH levels were assessed by kit. *n* = 3. **D** The expressions of GCLC, GCLM and NQO1 were detected in hippocampal tissues by qRT-PCR. *n* = 3. **E** The expressions of CD86 and CD206 were assessed by IHC in hippocampal tissue. *n* = 3. ^*^P < 0.05, vs the Model; ^#^*P* < 0.05, vs the NOB.

It was found that EX527 treatment decreased the number of times rats in the NOB group crossed the original platform within 2 min and increased the time the model rats to find the platform (Fig. 6A). The levels of BMAL1, SIRT1, E2F1, and NAD^+^/NADH in hippocampal tissues were lower in the NOB + EX527 group than in the NOB group (Fig. 6B, C). EX527 reversed the increase of GCLC, GCLM, and NQO1 expressions in the NOB group (Fig. 6D). In addition, the EX527 treatment promoted the expressions of the M1-type microglia marker CD86 in the NOB group. EX527 treatment reversed the rise in the expressions of the M2-type microglia marker CD206 in the NOB group (Fig. 6E). The above results suggested that NOB nanoparticles promoted microglia M2 polarization and inhibited M1 polarization through activation of SIRT1 to ameliorate chronic PSD-induced cognitive impairment in rats.

## Discussion

Plant-derived nanoparticle cocc 30c can improve motor ability in rats [30]. Armodafinil nanocrystal nasal hydrogel may alleviate cognitive dysfunction in rats through a protective effect on cortical area 1 hippocampal neurons, which is closely related to cognitive function [31]. There is limited research on nanoparticle use in animal models induced by chronic PSD. This study reports, for the first time, that NOB nanoparticles could alleviate inflammation and cognitive impairment induced by chronic PSD in rats. The study reported for the first time that NOB nanoparticles could alleviate inflammation and cognitive impairment induced by chronic PSD in rats.

NOB can regulate the expression of the core clock gene BMAL1 and serve as a nutritional preventive strategy for restoring metabolic disorders related to circadian rhythms [32]. Similarly, this study found that NOB nanoparticles could upregulate BMAL1 and affect inflammation and cognitive impairments in the model. NOB can mitigate cell inflammation induced by palmitic acid through SIRT1 modulation [33]. BMAL1 mediates SIRT1 regulation in renal ischemia-reperfusion injury [34]. BMAL1 regulates muscle insulin sensitivity in male mice through SIRT1 [35]. Hydrogen sulfide reduces hippocampal damage by upregulating hippocampal SIRT1 in the CSD model [36]. Consistent with these studies, the current study found that SIRT1 was a downstream target of the BMAL1-mediated effects of NOB nanoparticles in the model. BMAL1 protein directly could bind to SIRT1 protein in microglial cells to activate SIRT1 expression, thereby regulating downstream signaling. E2F1 mediates brain damage induced by pneumococcus [37]. SIRT1 is an important regulatory factor in postnatal brain injury for promoting oligodendrocyte precursor cell proliferation, and its mechanism is related to mediating E2F1 regulation [29]. SIRT1 can bind to E2F1 to regulate DNA damage-induced apoptosis [38]. This study revealed the alleviating effects of NOB nanoparticles on inflammation and cognitive impairments induced by chronic PSD through the BMAL1/SIRT1/E2F1 axis. Moreover, NAD^+^ metabolism mediated the effects of NOB nanoparticles on LPS-induced microglial M1 polarization and the BMAL1/SIRT1/E2F1 axis.

M1 microglial cell polarization promotes diabetes-related cognitive dysfunction [39]. Hippocampal TREM2 plays an important role in improving high-fat diet-induced cognitive dysfunction and promoting the polarization of microglial cells towards the M2 anti-inflammatory phenotype [40]. Melatonin upregulates BMAL1 and CD206, inhibits CD86 to alleviate oxidative stress, and thereby reduces CSD-related cognitive impairments [25]. Similar to the aforementioned studies, our research found that NOB nanoparticles could reverse the decrease in BMAL1, SIRT1, E2F1, and CD206 expression induced by chronic PSD, as well as the increase in CD86 expression, thereby alleviating cognitive impairments. Many endogenous antioxidants are involved in maintaining redox balance and preventing and treating central nervous system diseases, including GCLC, GCLM, and NQO-1 [41]. Quercetin increases the expression levels of M2 marker IL-10 and GCLC, GCLM, and NQO1 [42]. Similar to the aforementioned studies, our research found that in an animal model induced by chronic PSD, NOB nanoparticles increased the expression of GCLC, GCLM, and NQO1.

Cognitive impairment is an important phenotype of the CSD model [43, 44]. Genistein and soy isoflavones may improve cognitive deficits in the CSD model, including in cognitive behavior tests such as the MWM, possibly through their antioxidant properties [45, 46]. Melatonin treatment attenuates hippocampal-dependent spatial learning and memory deficits induced by chronic rapid eye movement sleep deprivation, and this mechanism may be associated with the BMAL1/Clock pathway [47]. Long-term caffeine treatment can prevent cognitive deficits, including hippocampal-dependent learning and short-term memory in the CSD models [48]. In the current study, it was found that treatment with NOB nanoparticles increased the number of times the model rats entered the central zone and crossed the original platform within 2 min while reducing the time it took for the model rats to find the platform, revealing the potential of NOB nanoparticles to alleviate the decline in exploratory and spatial learning and memory abilities in chronic PSD-induced rats. This mechanism is associated with the activation of the BMAL1/SIRT1/E2F1 axis.

Our in vitro experiments only demonstrated the anti-inflammatory effects of NOB nanoparticles, BMAL1, and the regulation of SIRT1/E2F1 pathway under LPS-induced microglia M1 polarization, which were confirmed in two animal experiments. However, we did not delve into the interaction of microglia with other cells, such as neurons, which is a limitation of this study and a new direction for subsequent research. Finally, this study confirmed that NOB nanoparticles upregulated BMAL1 to inhibit microglia M1 polarization through SIRT1/E2F1 pathway to improve cognitive impairment in chronic insomnia rats, providing a new theoretical basis for the treatment of this disease.

## Conclusion

NOB nanoparticles alleviate chronic PSD-induced microglia M1 polarization, inflammation, and cognitive deficits in rats by a mechanism that may be related to the BMAL1/SIRT1/E2F1 axis. Our study provides a new and novel direction for the therapeutic approach to CSD-associated cognitive impairment.

## Materials and methods

### Preparation of NOB nanoparticles from amorphous solid dispersions

Wet-milled NOB nanoparticles were prepared as described previously [14]. Briefly, 100 mg of NOB was weighed into the vessel of a rotary/spinning mixer (NP-100; Thinky Company Ltd, Tokyo, Japan). Zirconia spheres with a diameter of 0.1 mm (Nikko Osaka, Japan) were placed into the vessel, and the indicated volume of hydroxypropyl cellulose SSL (HPC-SSL) solution (10 mg/mL) was added. The NOB suspension was nanosized by a two-step wet pulverization under the following pulverization conditions. In the first step, the NOB suspension dissolved in 0.6 mL of HPC-SSL solution was spun for 2 min at 2000 rpm; in the second step, the suspension was spun for 1 min at 400 rpm after the addition of 9.4 mL of HPC-SSL solution (total volume 10 mL). After nanosizing by wet milling, NOB suspensions in 20 mL vials containing 100 mg of milled NOB and 100 mg of HPCSSL were frozen with liquid nitrogen and freeze-dried using an FD-81 freeze-dryer (Tokyo Rikakikai, Tokyo, Japan). We examined the morphology and particle size of the prepared nanoparticles using TEM (JEM-1230, JEOL, Japan) and nanoparticle tracking analysis (NTA, NanoSight Ns300, Malvern Panalytical, UK), respectively, with reference to the previous studies [49, 50].

### Animals

**Fig. 7:**
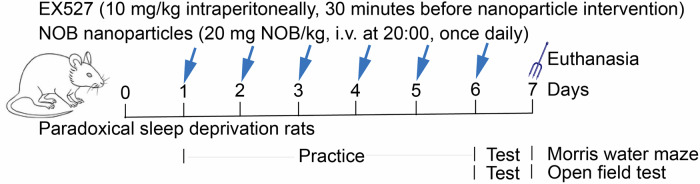
Flow chart of animal experiment. PSD rats were treated with NOB nanoparticles or EX527.

Based on variance analysis degrees of freedom and expected modeling success, we used 6 rats per group. Sprague-Dawley male rats (weighing 200–220 g, 6–8 weeks) were purchased from Hunan Slake Jinda Laboratory Animal Co. The model was constructed by applying a multi-platform method for 7 days of chronic PSD procedure [51]. The rats were individually placed in a water tank (170 × 44 × 44 cm) with a circular platform (6.5 cm in diameter in the Model group, 16 cm in diameter in the Control group, and 10 cm in height in both groups) that was 1 cm above the water surface. A platform with a diameter of 6.5 cm was small enough to completely eliminate paradoxical sleep. Loss of muscle tone would have awakened the animals from the water. Water and food were provided in a temperature-controlled room (24 ± 1 °C, 55 ± 15%) on a 12-h light/dark schedule (Lighting hours were 07:00-19:00) through a free passage suspended above the tank. Rats were randomized to Control, Model, Model+NOB nanoparticles (20 mg NOB/kg, i.v. at 20:00, once daily [14]) for the exploration of NOB nanoparticles treatment (Fig. 7).

After that, rats were randomly divided into Model, Model+NOB nanoparticles, and NOB nanoparticles+EX527 (EX-527, a SIRT1 inhibitor, 10 mg/kg intraperitoneally 30 min before nanoparticle intervention [52]) to explore whether NOB nanoparticles mediate the development of model through SIRT1 (Fig. 7). NOB nanoparticles and EX527 were administered for 7 days. On the seventh day following the establishment of the model, open field tests (OFT) were carried out from 17:00 to 19:00 pm. Morris water maze (MWM) were performed on days 2–7 after rat modeling. Specifically, rats were trained on days 2–6 during modeling and then tested on day 7. Finally, rats were euthanized by intraperitoneal injection of 150 mg/kg barbiturate and brain tissues were collected for subsequent experiments.

### Behavioral testing

#### OFT

According to previous studies [53], an open-field box measuring 40 × 40 × 40 cm was used to record the number of times the rats entered the central zone using smart junior software 3.0 (Panlab, Cambridge, USA).

#### MWM

Spatial learning and memory were assessed by the MWM, as described previously [54]. Briefly, the MWM was performed in a circular dark pool (50 cm high, 180 cm diameter) filled with white water (25 ± 1 °C). The rats were tasked with locating a hidden platform positioned ~1 cm beneath the water’s surface at the center of a specific quadrant in the pool. Each group of rats performed four consecutive training trials per day. Probe trials were then repeated without the platform. The time taken by rats to find the platform (escape latency) and the number of times they crossed the original platform within 2 min were recorded.

### Cell treatment

Human microglia cells (HMC3, AW-CNH003, Abiowell, China) were cultured in MEM medium containing 10% fetal bovine serum and placed in a thermostatic incubator (5% CO_2_, 37 °C). Cells were treated with lipopolysaccharide (LPS, 100 ng/mL) for 24 h [55]. Normal or LPS-induced cells were treated with different concentrations of NOB nanoparticles (6.25, 12.5, 25, 50, and 100 μg/mL) for 24 h to screen the optimal concentration of NOB nanoparticles for treating cells [56]. Cells were pretreated with apocynin (inhibitor of the rate-limiting enzyme of Nicotinamide adenine dinucleotide (NAD)^+^ biosynthesis, S2425, Selleck, USA) for 30 min before all drug interventions [57]. Lentiviral vectors encoding short hairpin RNA (shRNA) specific for BMAL1 (sh-BMAL1) and SIRT1 (sh-SIRT1) and the scrambled sequences as negative control (sh-NC) were used to transfect the cells prior to treatment with LPS. sh-BMAL1 lentiviral, sh-SIRT1 lentiviral, overexpression of BMAL1 (oe-BMAL1) plasmids and their scrambled sequences were purchased from Abiowell (China). All cell lines used in this study underwent rigorous quality control, including mycoplasma testing and STR-based authentication.

### Cell counting kit-8 (CCK-8)

CCK-8 kit (40203ES60, Yeasen, China) was utilized to assess cell proliferation. Cells were inoculated in 96-well plates at a density of 2000 cells/well. The next day, 10 μL of CCK-8 solution was added to each well. The cells were placed in an incubator at 37 °C and 5% CO_2_ and continued to incubate for 4 h. Afterward, an enzyme marker (MB-530, Shenzhen Huisong Technology Development Co., Ltd, China) was utilized to analyze the absorbance (OD) values at 450 nm.

### Quantitative real-time PCR (qRT-PCR)

The expressions of BMAL1, SIRT1, E2F transcription factor 1 (E2F1), glutamate-cysteine ligase catalytic subunit (GCLC), glutamate-cysteine ligase modifier subunit (GCLM), and NAD(P)H quinone oxidoreductase-1 (NQO1) genes were detected by qRT-PCR. Trizol (R0016, Beyotime, China) was used to extract total RNA from tissues and cells. Total RNA was reverse transcribed into cDNA after the concentration was determined. The Primer5 software is used to design primers, which are then synthesized by Tsingke Biotechnology Co., Ltd (China). Samples were subjected to qRT-PCR in a PCR instrument (PIKOREAL96, Thermo, USA) at 95 °C for 10 min, 40 cycles at 95 °C for 15 s and at 60 °C for 30 s, with β-actin as the internal reference. The primer sequences are shown in Table 1, and the relative expression was calculated by the 2^-ΔΔCT^ method.

**Table 1:** Primer sequences.

Organisms	Name	Primer sequences	Product length (bp)
Human	β-actin	F ACCCTGAAGTACCCCATCGAG	224
		R AGCACAGCCTGGATAGCAAC	
	BMAL1	F CAGGGCAGCAGATGGATT	187
		R TGCGGTGTCAGAGGAGGA	
	SIRT1	F ATTCCAAGTTCCATACCCCAT	145
		R TGGCATATTCACCACCTAACCT	
	E2F1	F TGCCCCACCCTCCAATCTGC	102
		R CAAAACCCGGCCCAAACGTCA	
Rat	β-actin	F ACATCCGTAAAGACCTCTATGCC	223
		R TACTCCTGCTTGCTGATCCAC	
	GCLC	F TGGAGACCAGAGTATGGGAGT	121
		R CTGGGAAATGAAGTTATCGTG	
	GCLM	F GAAAAAGTGTCCGTCCACGC	88
		R ATCAGGGCTGATTTGGGAGC	
	NQO1	F CTTGCTTTCAGTTTTCGCCTT	170
		R GCCCCTAATCTGACCTCGTTC	

### Western blot

Proteins were extracted from cells or tissues as described previously [25, 45]. Total proteins were separated using SDS-PAGE and then transferred to PVDF membranes (GE Healthcare Life, USA). Primary antibodies against BMAL1, GCLC, GCLM, NQO1, SIRT1, E2F1, and β-actin were added and incubated overnight at 4 °C, followed by the addition of a secondary antibody for 90 min. Finally, the protein levels were quantified by analyzing the bands using the Image J software (β-actin for internal reference). The antibody sequences are shown in Table 2. Original uncropped blot images are provided in Supplementary Fig. 1.

**Table 2:** Antibody information.

Name	Catalog Number	Source	Dilution ratio	Molecular weight (KDa)	Transfer time (minutes)	Company
BMAL1	AWA11417	Rabbit	1:5000	69–75	95	Abiowell (China)
GCLC	AWA11233	Rabbit	1:20,000	73	90	Abiowell
GCLM	AWA11593	Rabbit	1:1000	31	50	Abiowell
NQO1	AWA11105	Rabbit	1:50,000	31	50	Abiowell
SIRT1	AWA10058	Rabbit	1:3000	110–130	150	Abiowell
E2F1	AWA10977	Rabbit	1:1000	47	65	Abiowell
β-actin	AWA00028	Mouse	1:5000	42	60	Abiowell
Goat anti-Rabbit IgG	AWS0002a	–	1:5000	–	–	Abiowell
Goat anti-Mouse IgG	AWS0001a	–	1:5000	–	–	Abiowell

### Enzyme-linked immunosorbent assay (ELISA)

The levels of interleukin (IL)-1beta (IL-1β, CSB-E08053h or CSB-E08055r), tumor necrosis factor alpha (TNF-α, CSB-E04740h or CSB-E11987r), chemokine (C-C motif) ligand 2 (CCL2, CSB-E04655h or CSB-E07429r), IL-4 (CSB-E04633h or CSB-E04635r), insulin-like growth factor-1 (IGF-1, CSB-E04580h or CSB-E04582r), transforming growth factor beta1 (TGF-β1, KE00002 or CSB-E04727r), CD86 (CSB-E08543r), cyclooxygenase-2 (Cox-2, CSB-E13399r), inducible NOS (iNOS, CSB-E08325r), interferon-gamma (IFN-γ, CSB-E04579r), CD206 (SEB542Ra), granulocyte colony-stimulating factor (G-CSF, CSB-E07340r), and granulocyte-macrophage colony-stimulating factor (GM-CSF, CSB-E04570r) in the samples were detected using ELISA kits, according to the manufacturer’s instructions. The ELISA kit for TGF-β1 was obtained from Proteintech (USA), while the rest were purchased from CUSABIO (China).

### Measurement of NAD^+^/NADH

The NAD^+^/NADH ratio was measured via an NAD^+^/NADH assay kit (S0175, Beyotime, China), according to the manufacturer’s instructions.

### Immunohistochemistry (IHC)

IHC was performed according to the previous methods [58]. Sections treated with primary antibodies against CD86 (AWA45516, Abiowell, China) and CD206 (AWA44419, Abiowell, China) were incubated at 4 °C overnight. Subsequently, sections were treated with horseradish peroxidase-conjugated secondary antibodies (PV-9001, ZSGB-BIO, China) at 4 °C for 30 min. DAB substrate kit (ZLI-9018, ZSGB-BIO, China) was used for staining. All sections were observed using a microscope (BA410T, Motic, China).

### Flow cytometry (FCM)

Cells were incubated with 5 μL of CD86 antibody (17-0869-42, eBioscience, USA) and then incubated for 30 min at room temperature away from light. Membrane-broken cells were incubated with 5 μL of CD206 antibody (17-2069-42, eBioscience, USA) and incubated for 30 min at room temperature away from light. Cells were washed and resuspended and were placed in a flow cytometer (A00-1-1102, Beckman, USA) for detection.

### Co-immunoprecipitation (coIP)

Cell extracts were prepared as described above and divided into three aliquots. IP was performed using protein A/G beads according to the manufacturer’s instructions (Santa Cruz, USA). Supernatants were incubated with IgG, E2F1 or SIRT1 antibodies and protein A/G beads. After removing the unbound supernatant, the beads were rinsed 3 times. Then, the proteins were separated using SDS-PAGE, transferred to PVDF membranes, and detected with E2F1 or SIRT1 antibodies.

### Statistical analysis

To minimize bias, randomization and blinding were strictly maintained throughout the study. Data are expressed as mean ± standard deviation (SD). To assess data distribution patterns, the Shapiro–Wilk test was first performed on both normal and log-normal datasets. If the data were normally distributed, Student’s *t*-test was used for group comparisons. For analyses involving three or more groups, one-way ANOVA with Tukey’s HSD post hoc testing was applied to determine statistically significant differences. *p* < 0.05 was considered significant.

## Supplementary information

Supplementary Figure 1

## Data Availability

Data will be made available upon reasonable request.
