# Supplementary material for: Nobiletin (NOB) nanoparticles ameliorate chronic paradoxical sleep deprivation (PSD)-induced cognitive deficits in rats
Source: Cell Death Discov. 2025 Oct 13;11:458. doi: 10.1038/s41420-025-02738-9 (PMC12518861; doi:10.1038/s41420-025-02738-9)

Figure 2F

BMAL1

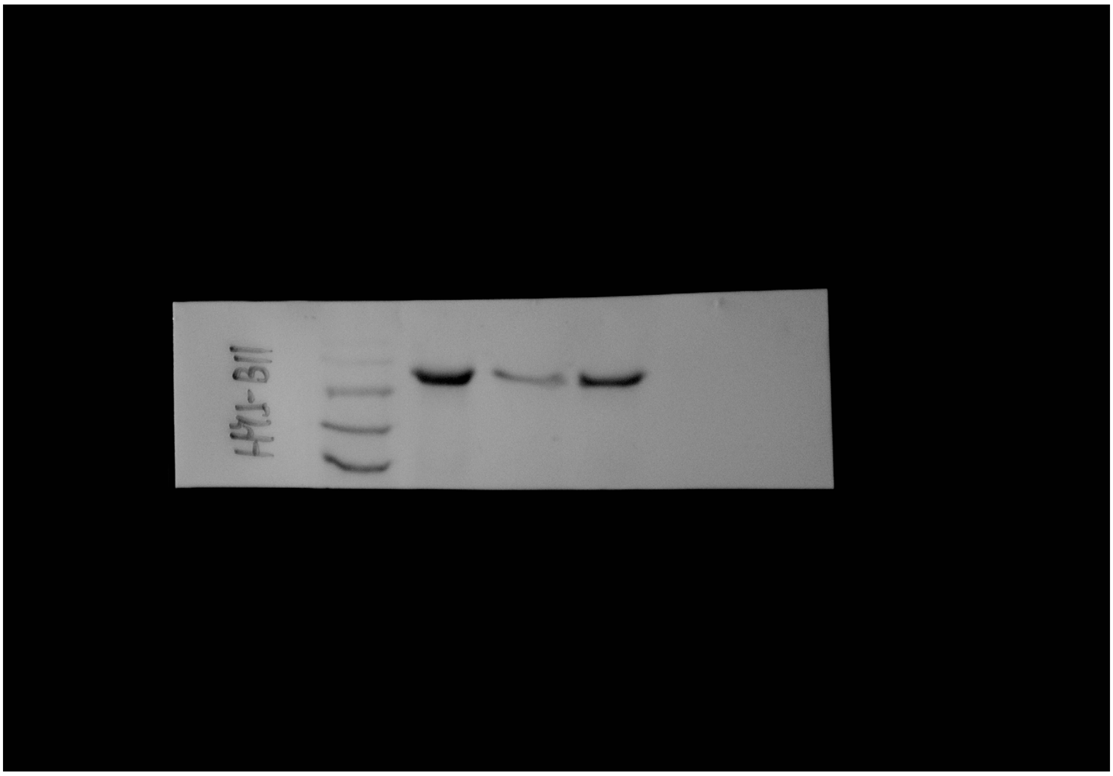

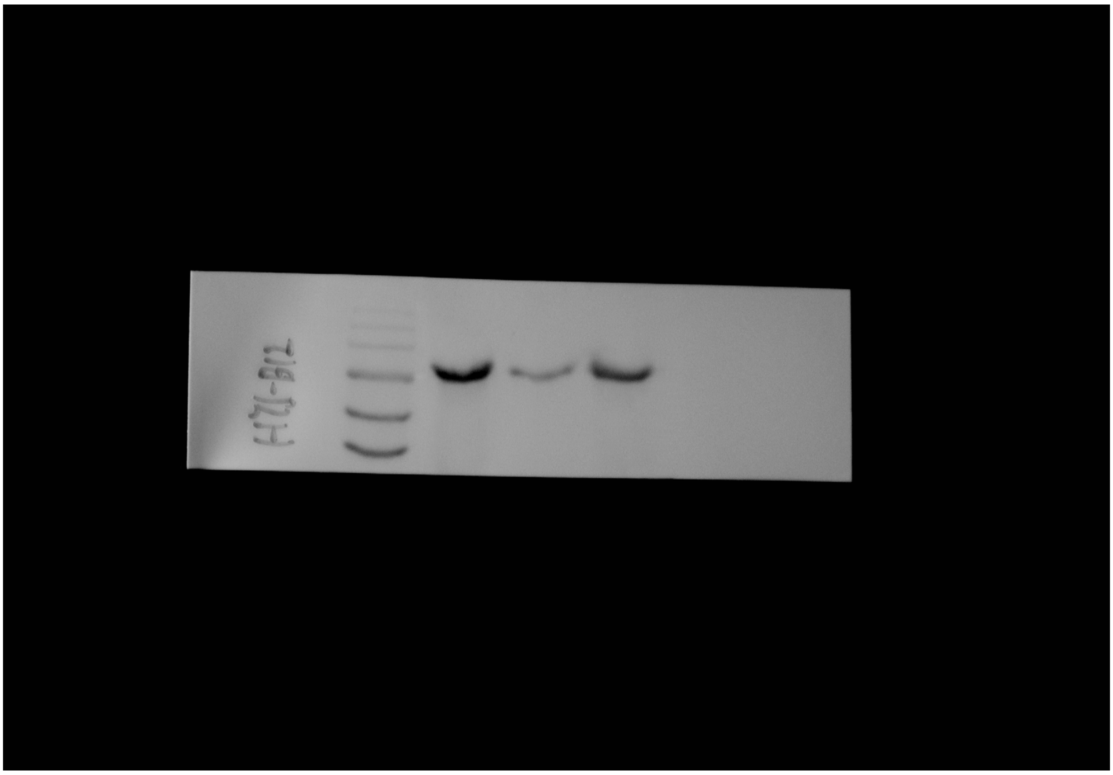

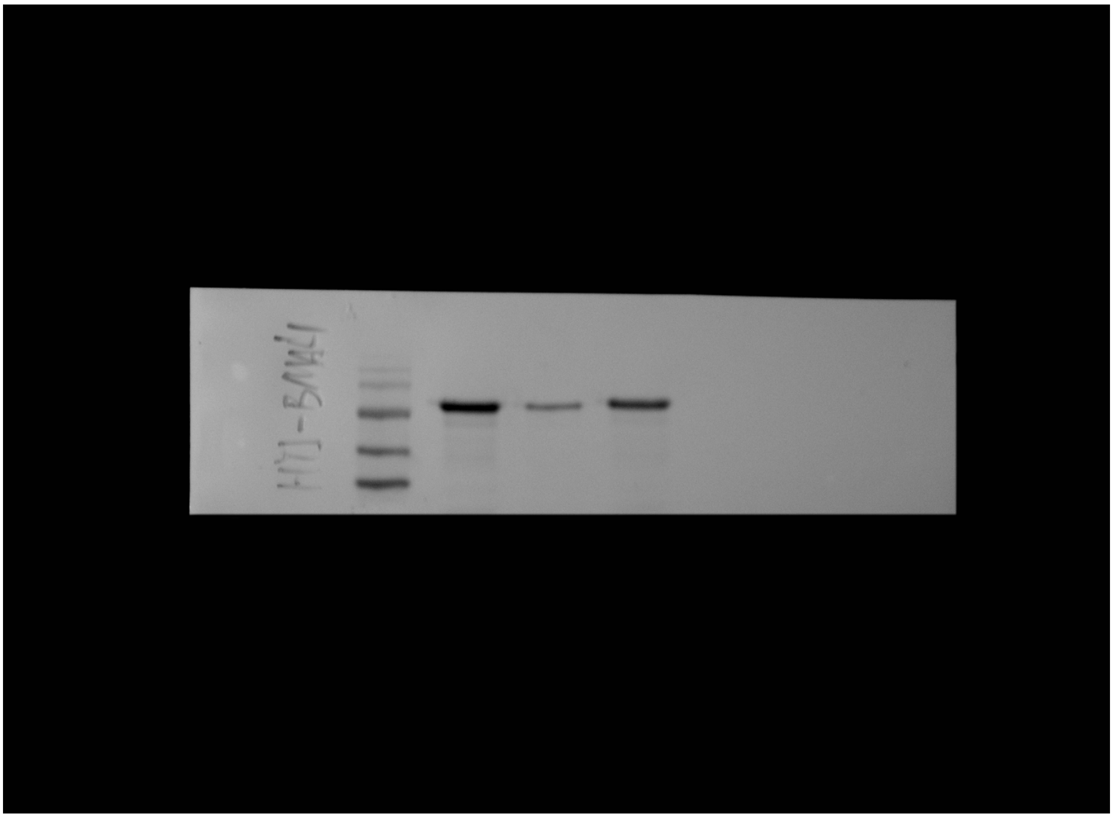

β-actin

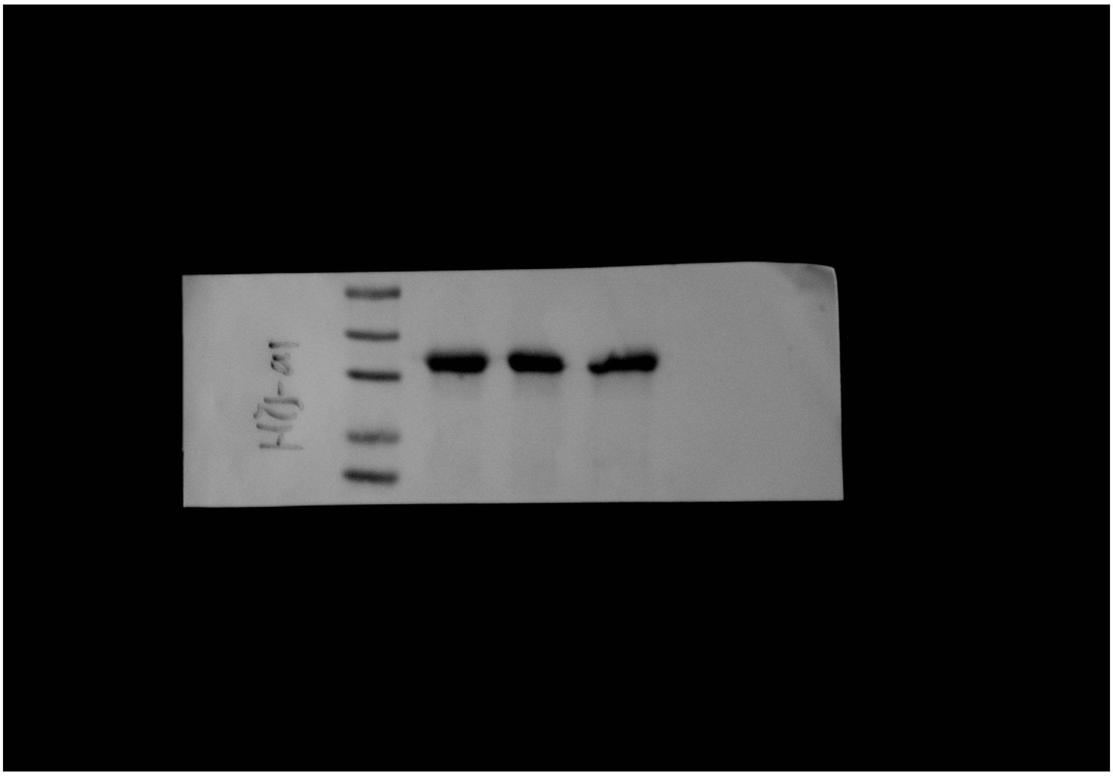

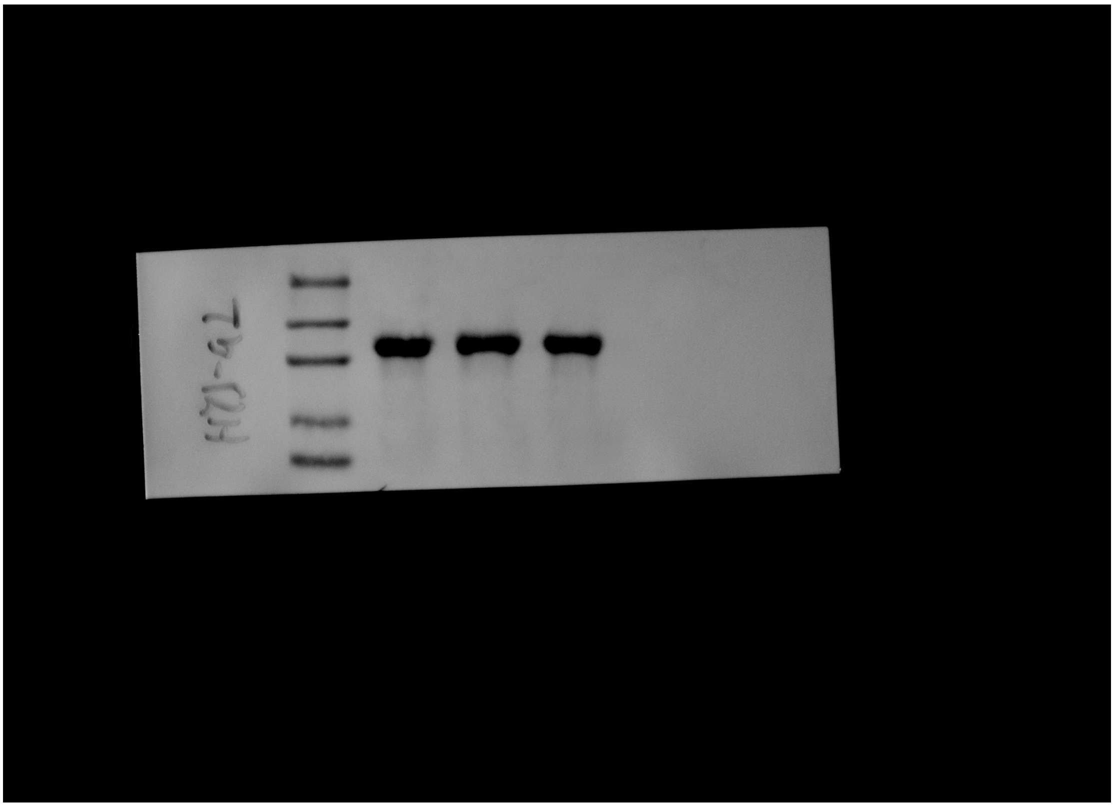

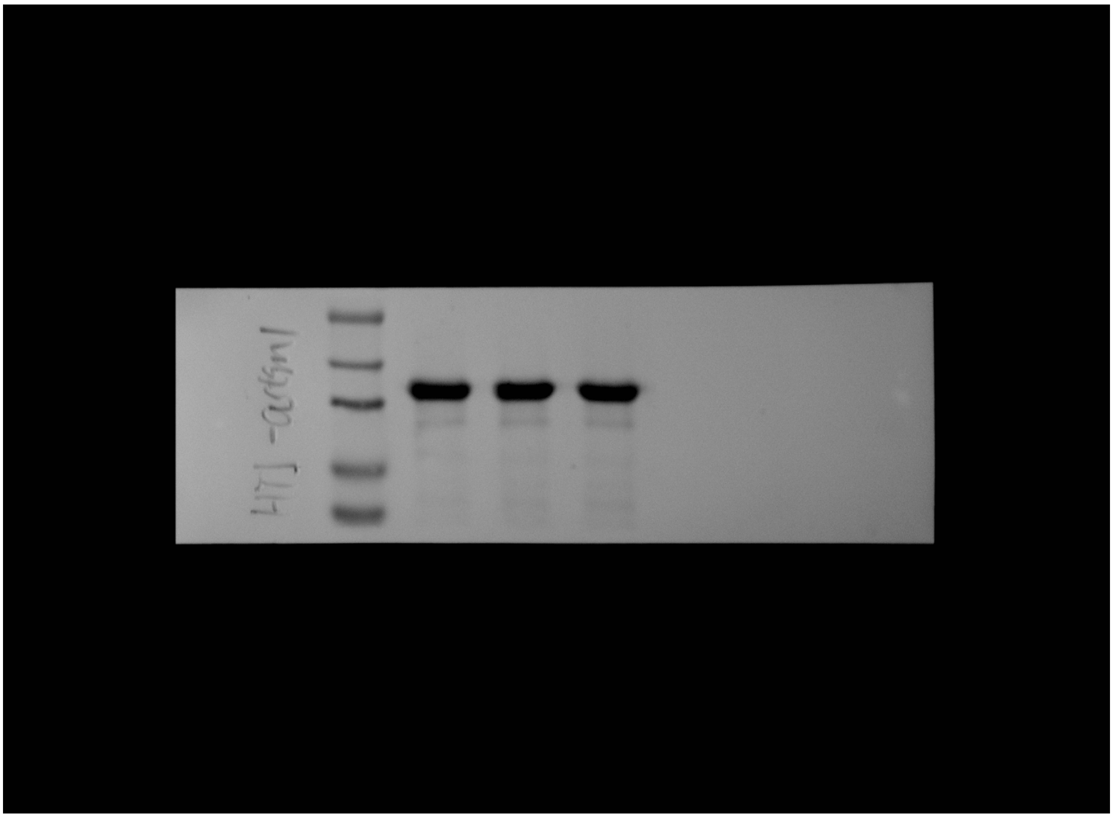

Figure 2H

GCLC

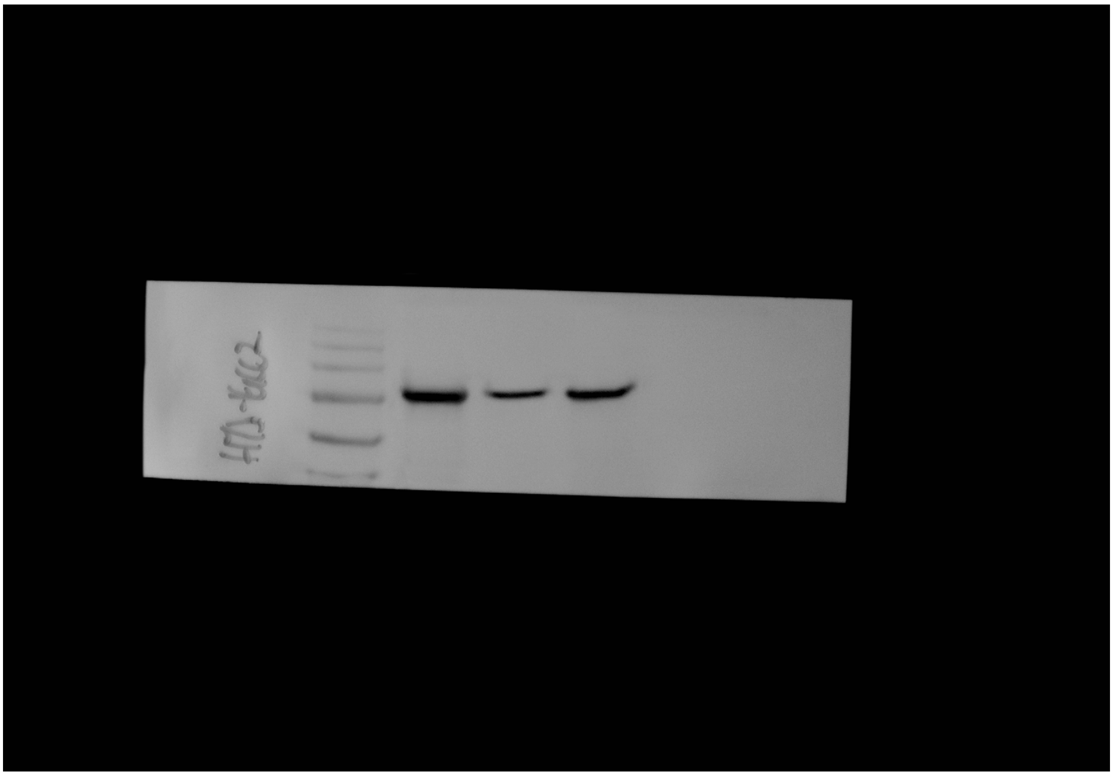

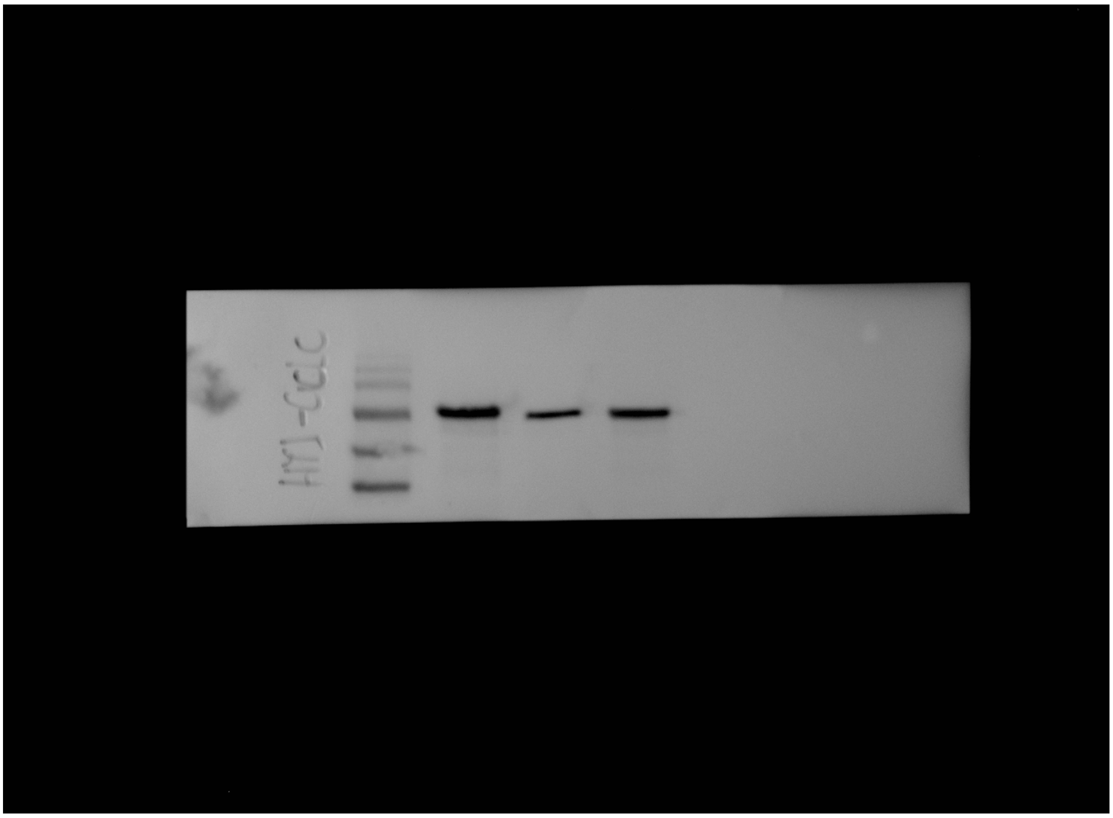

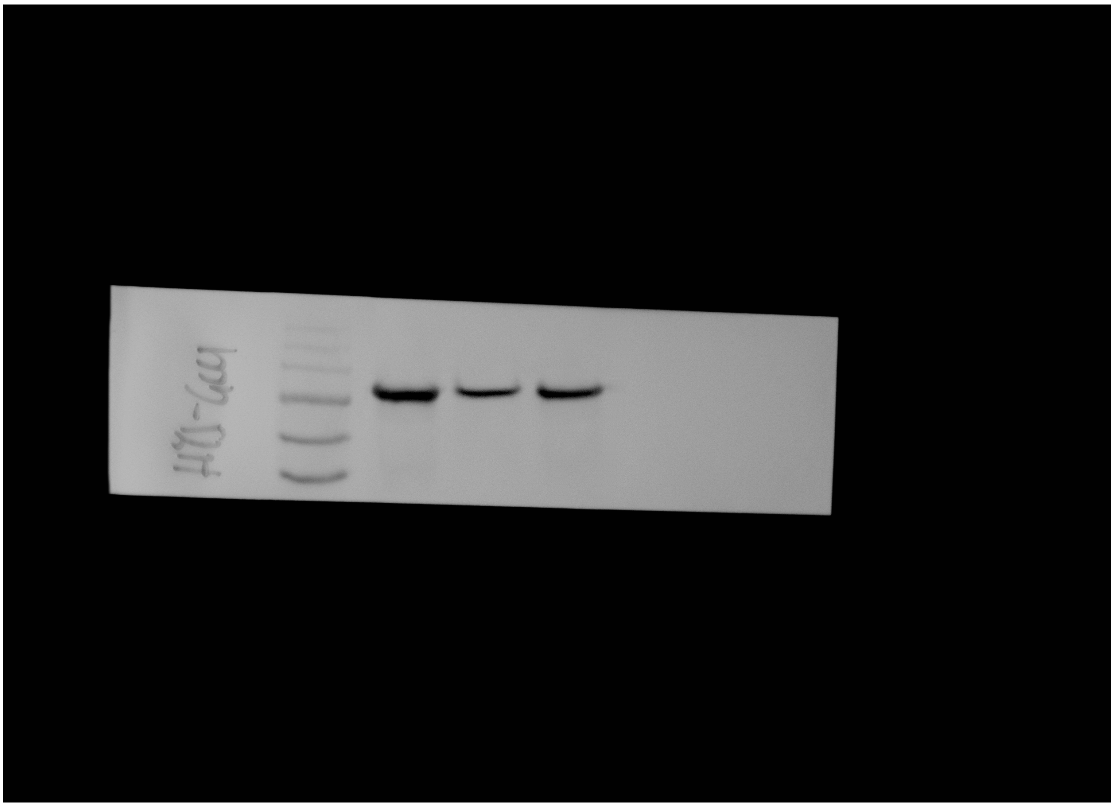

GCLM

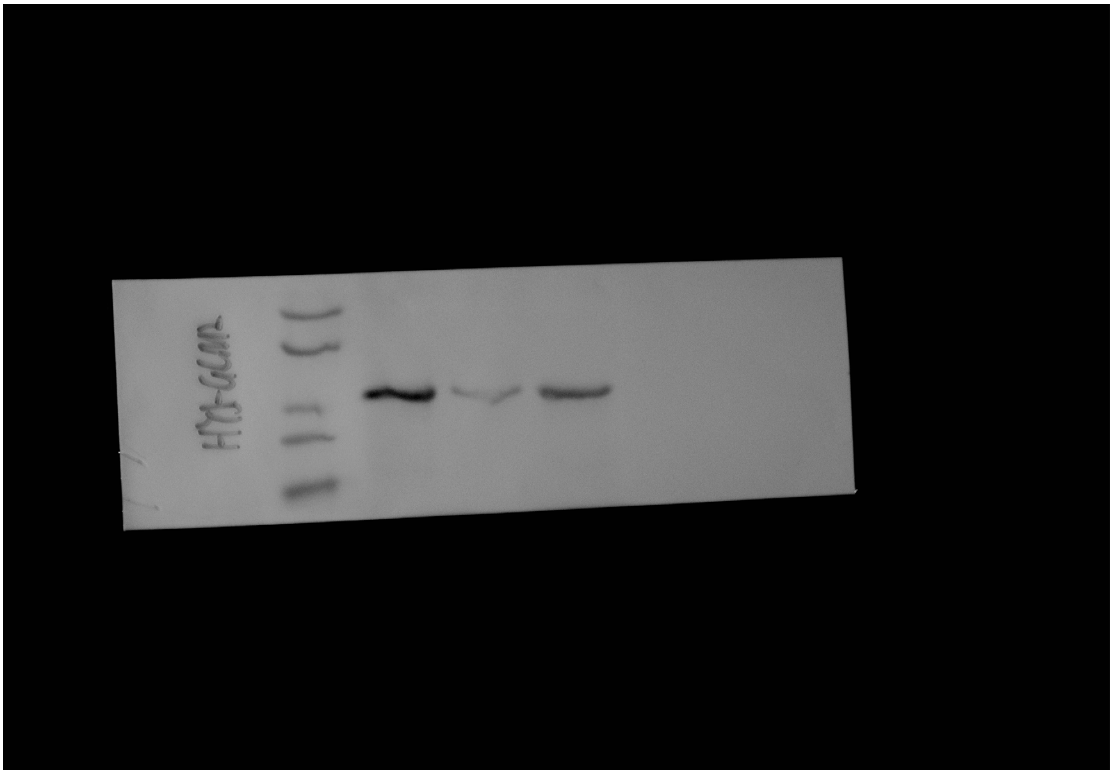

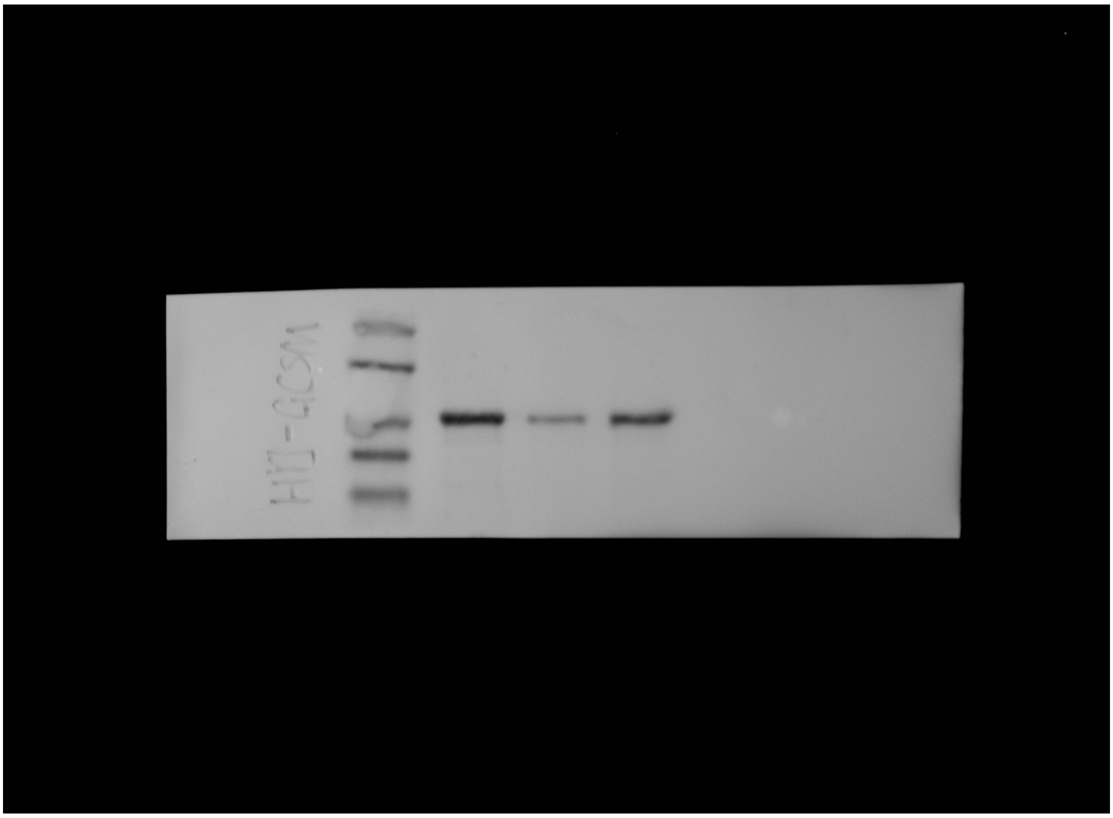

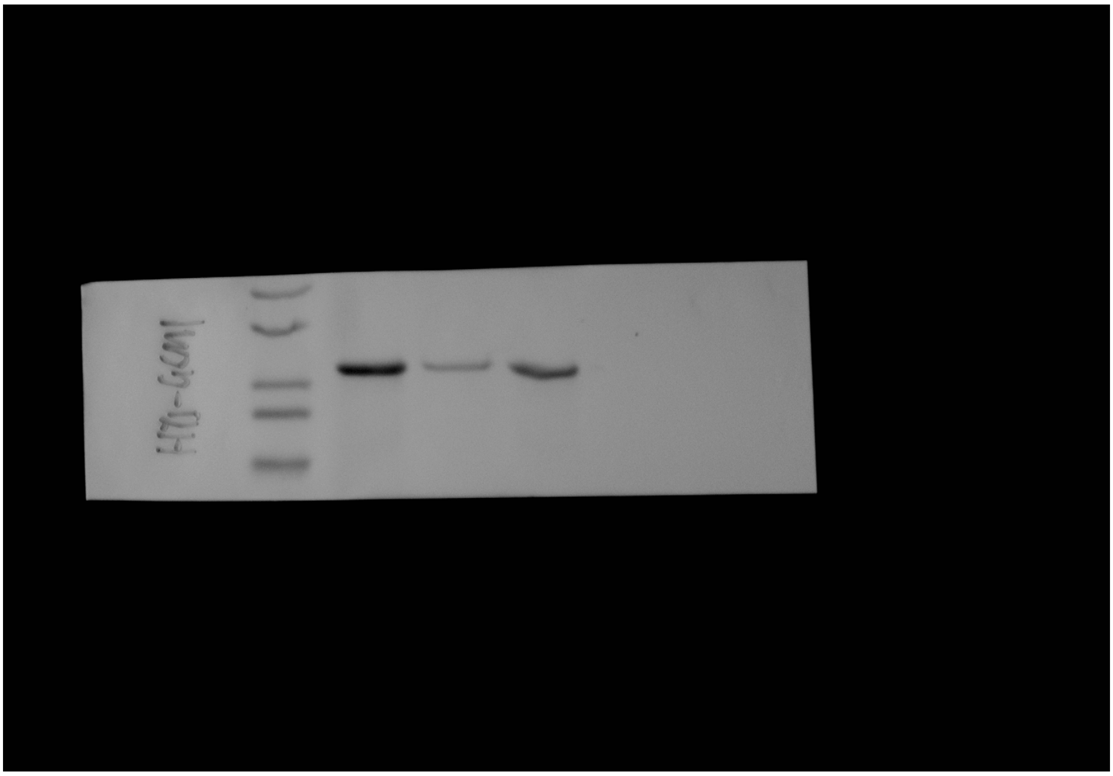

NQO1

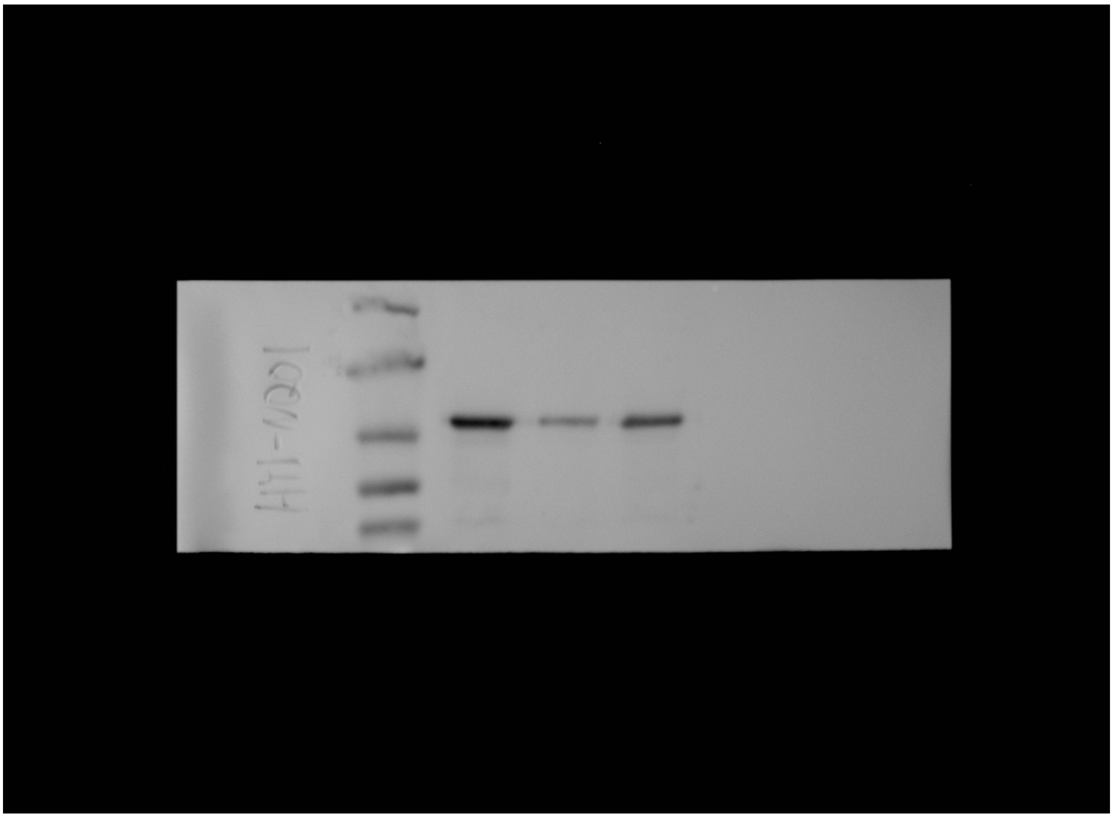

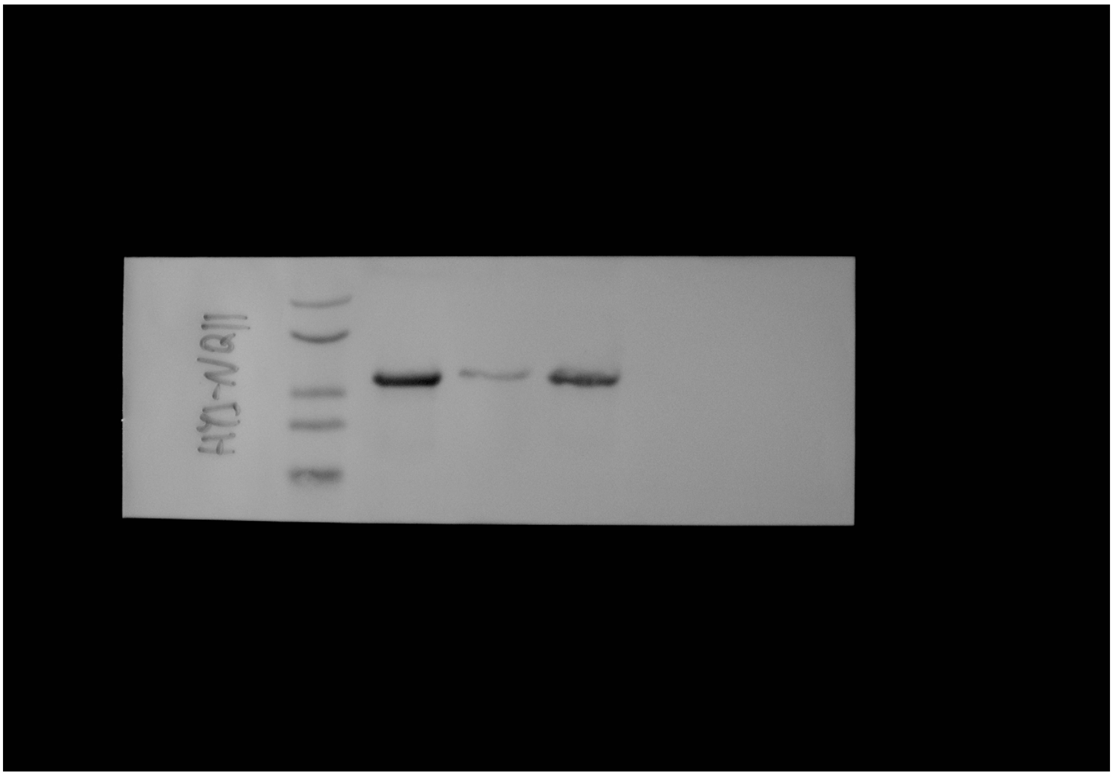

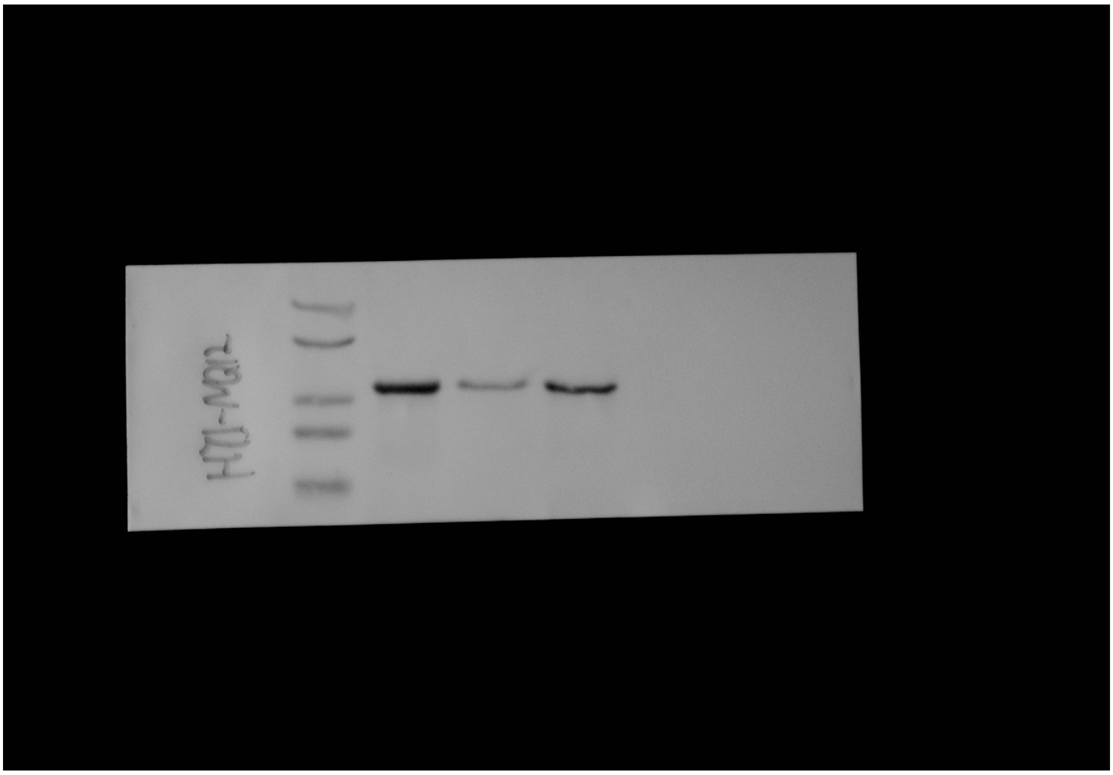

β-actin

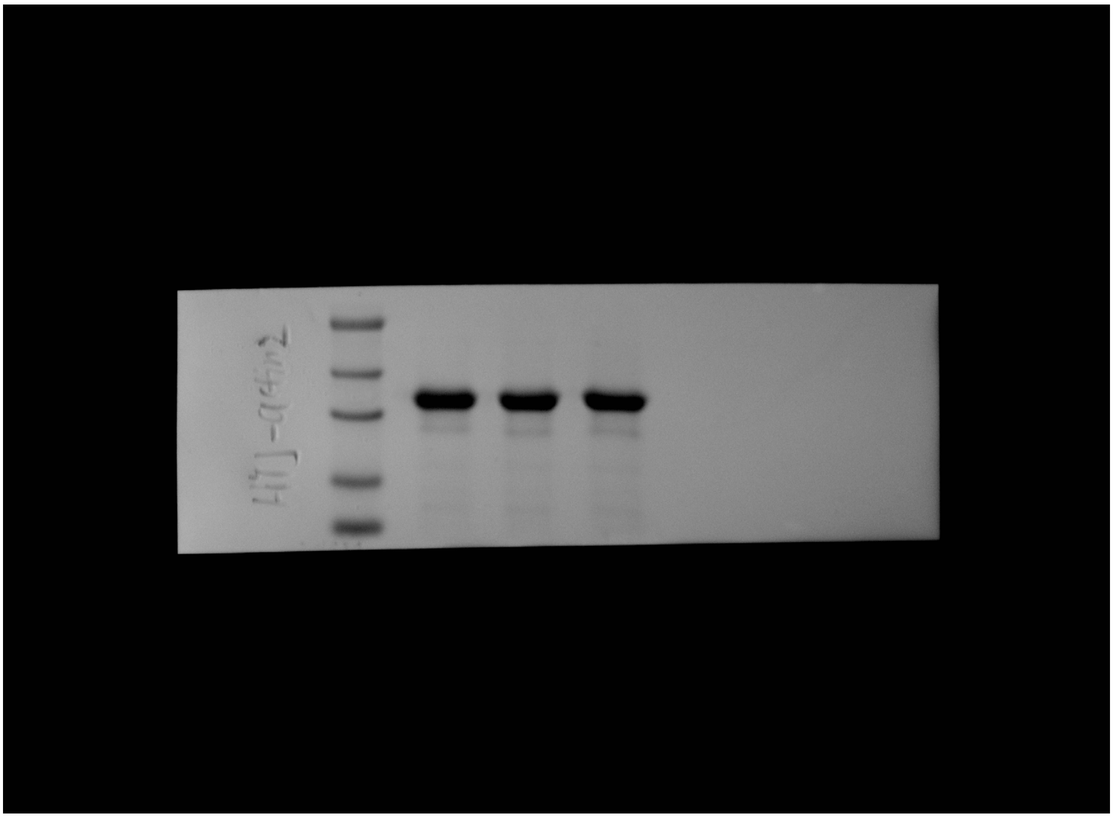

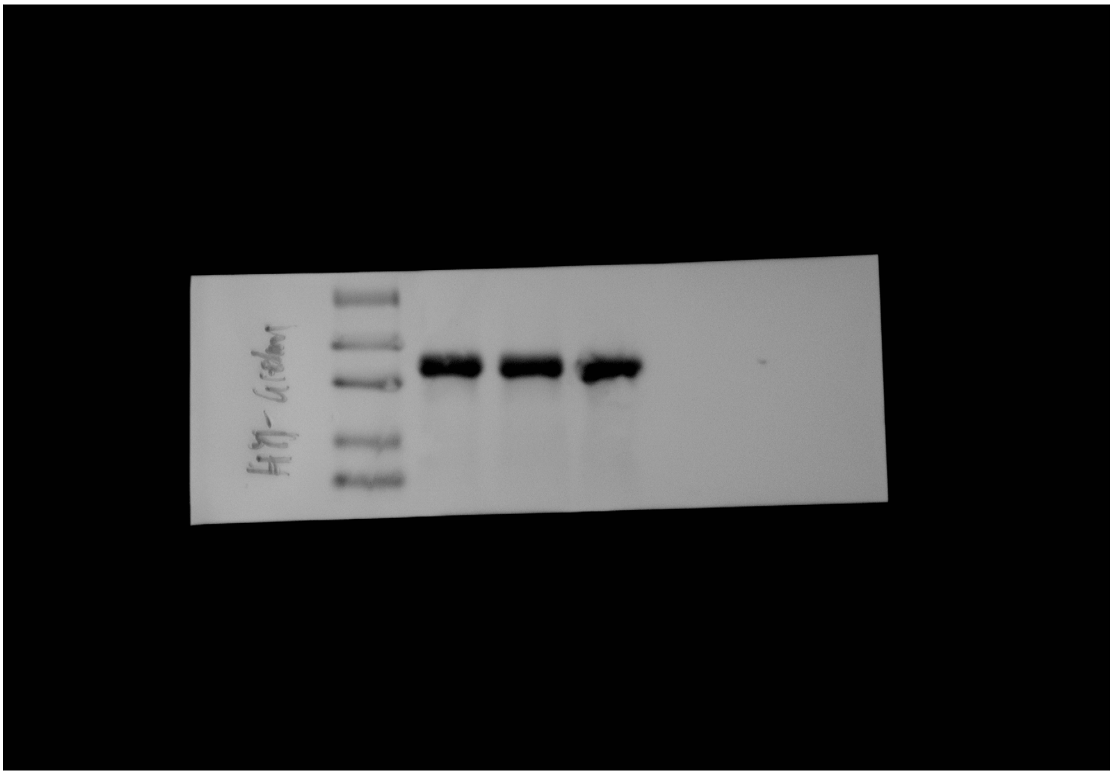

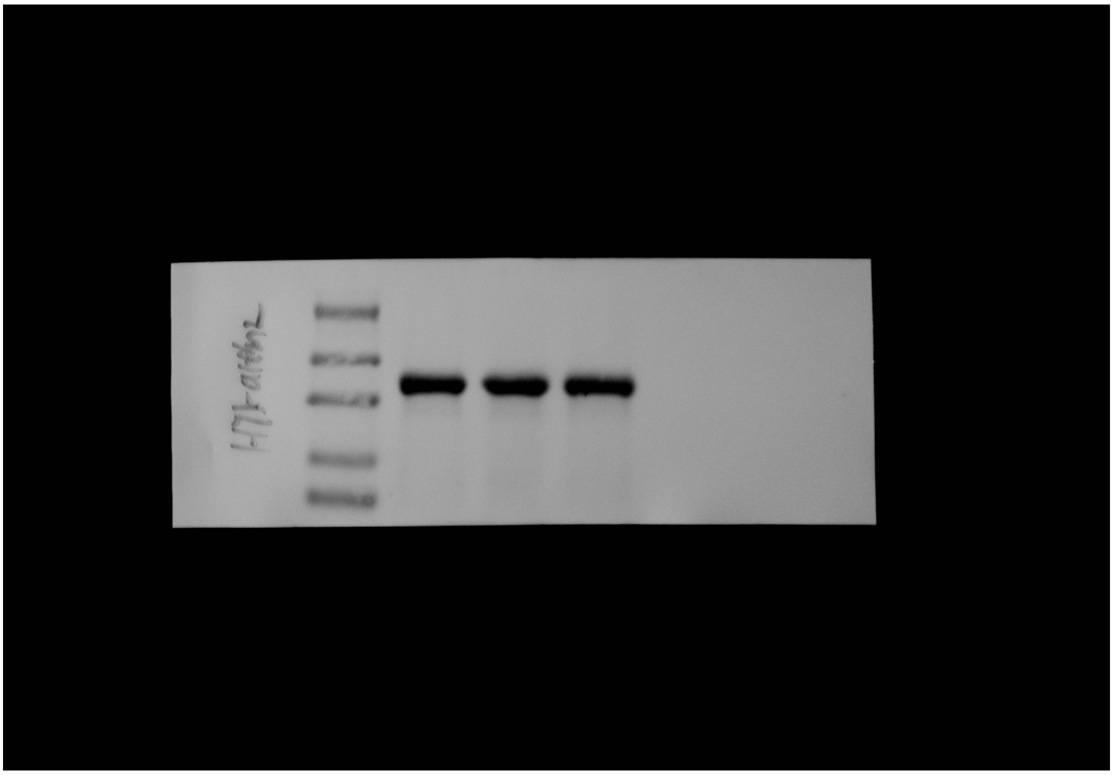

Figure 3C

BMAL1

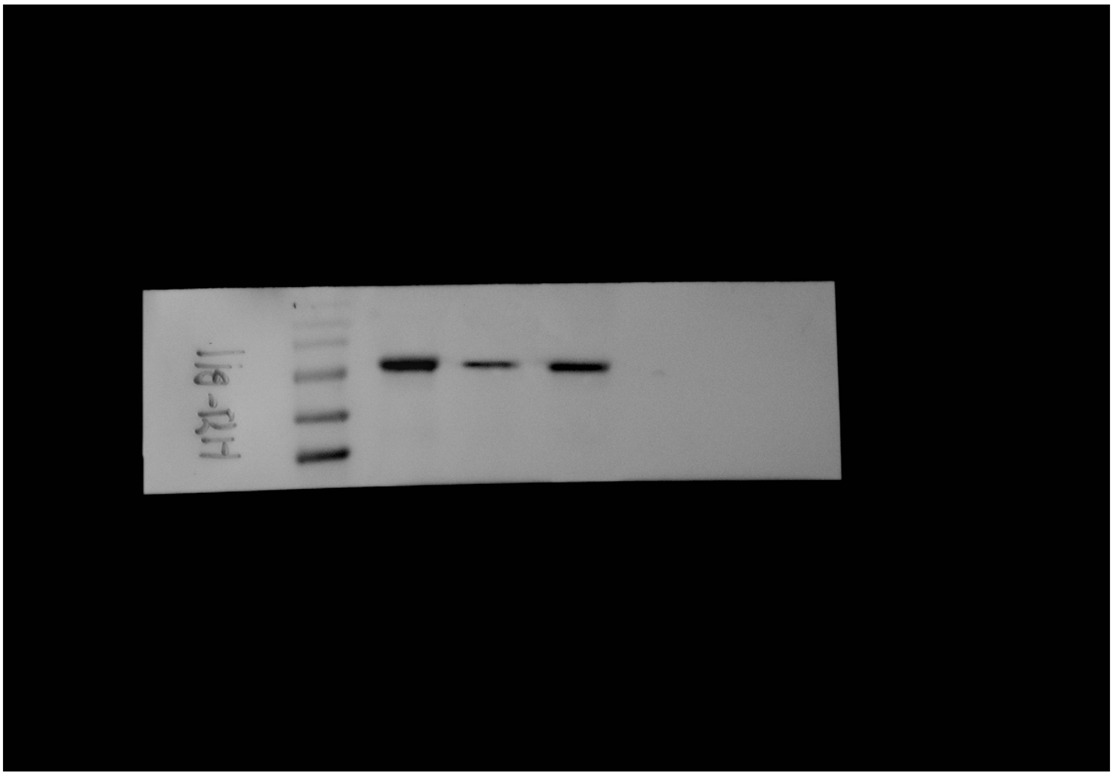

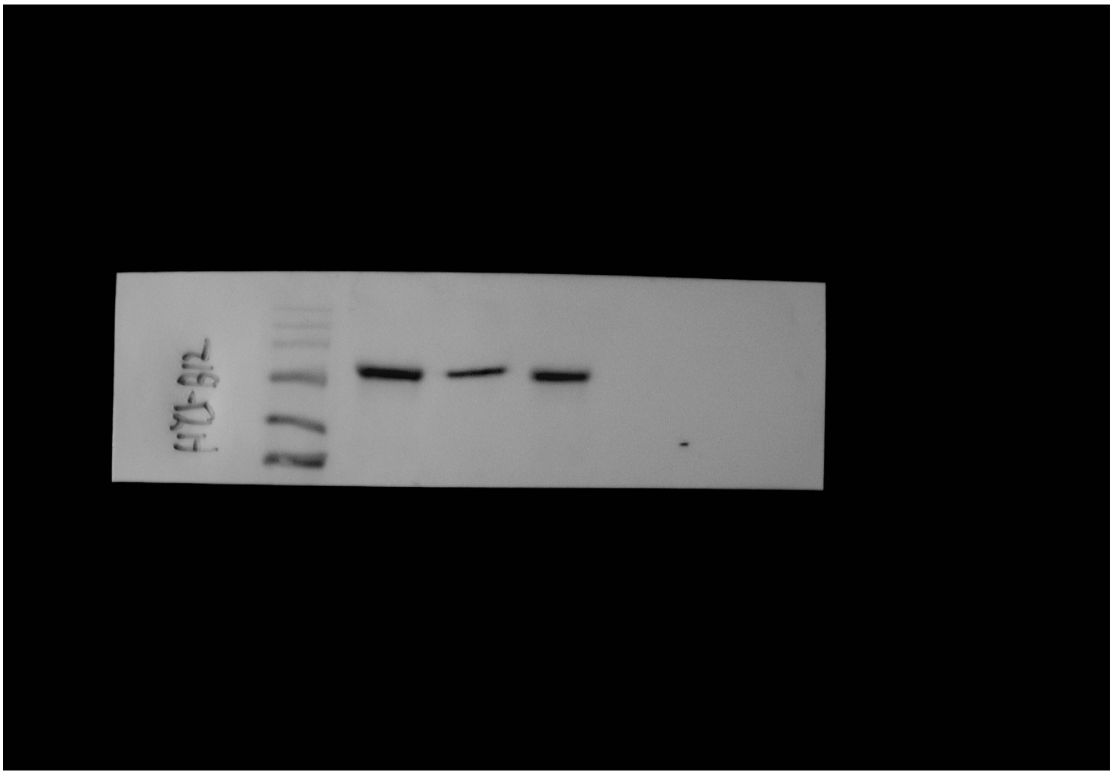

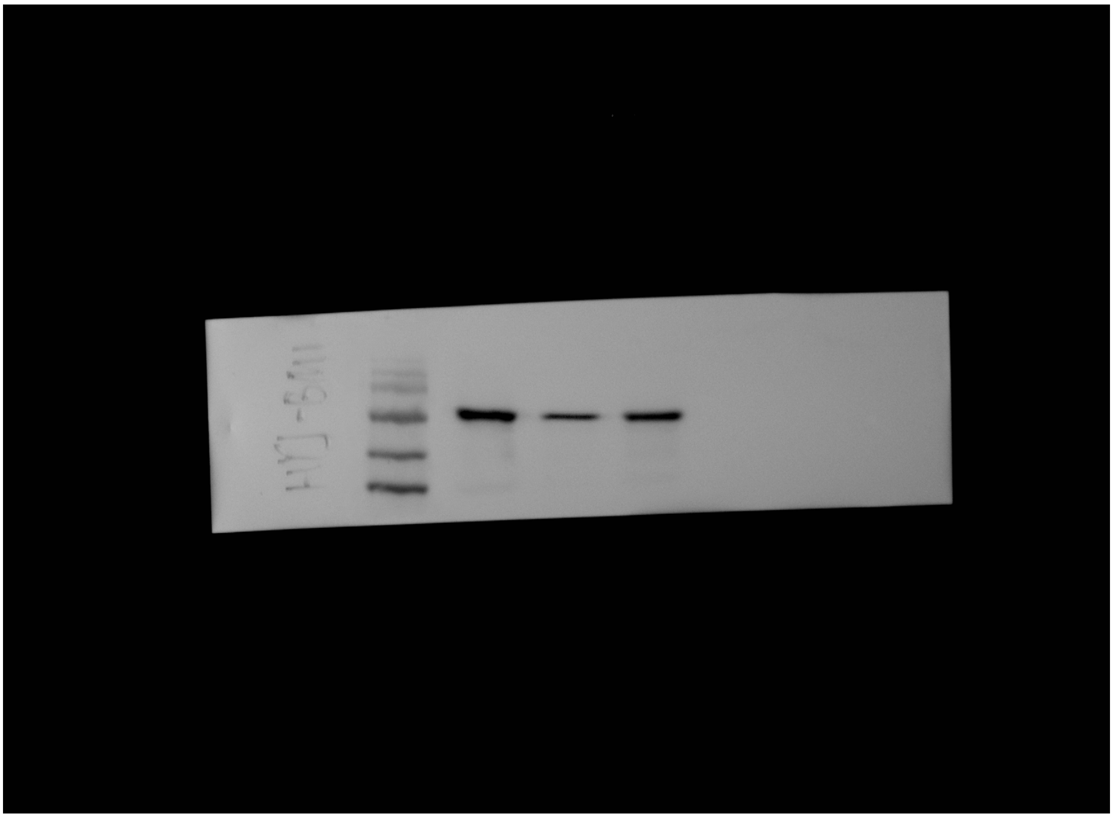

E2F1

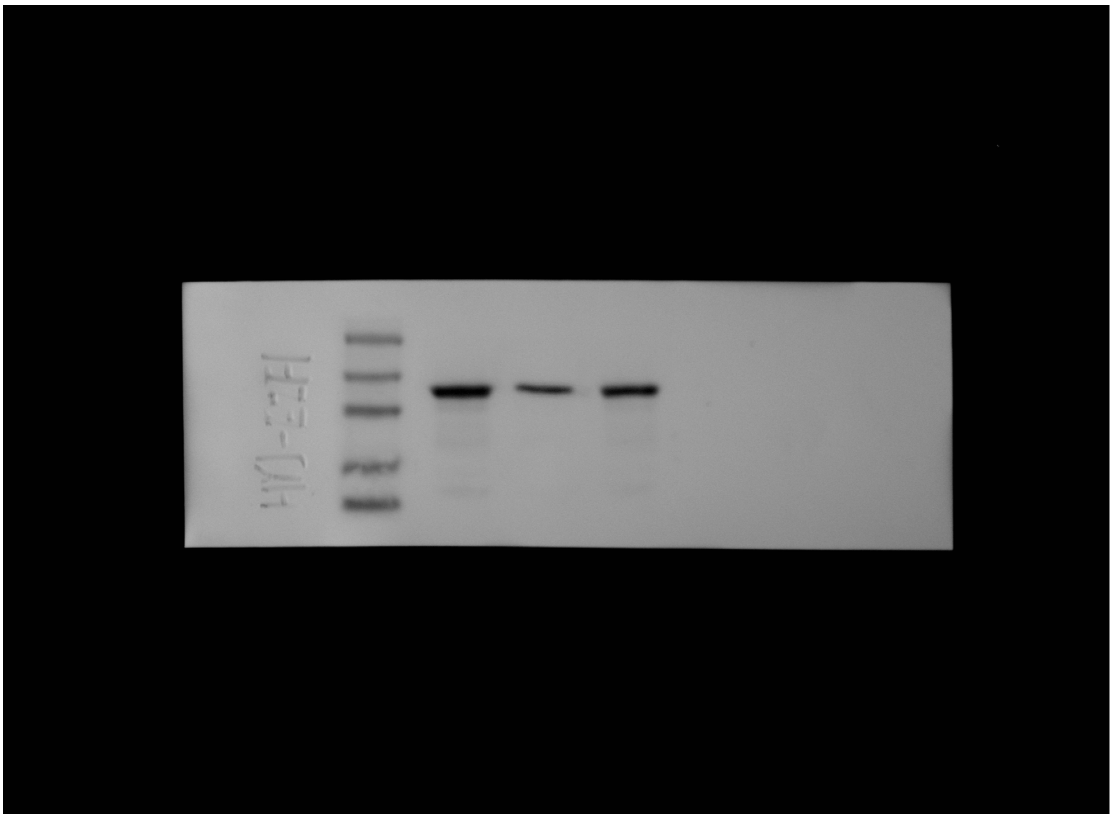

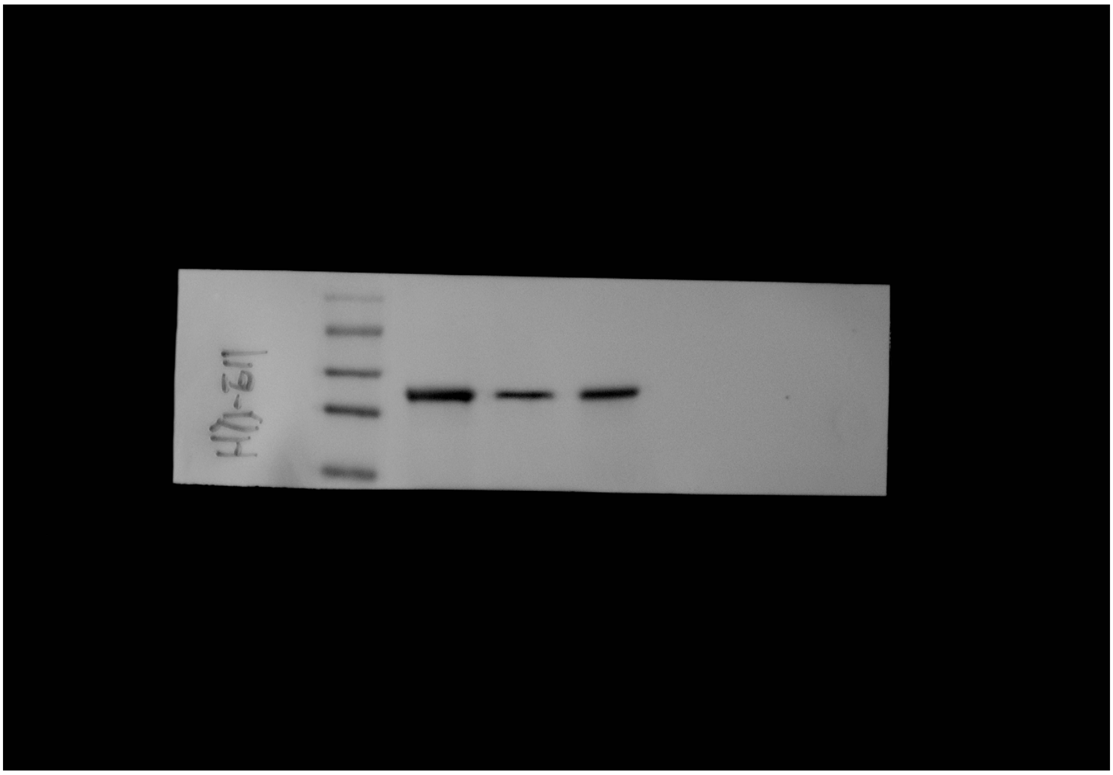

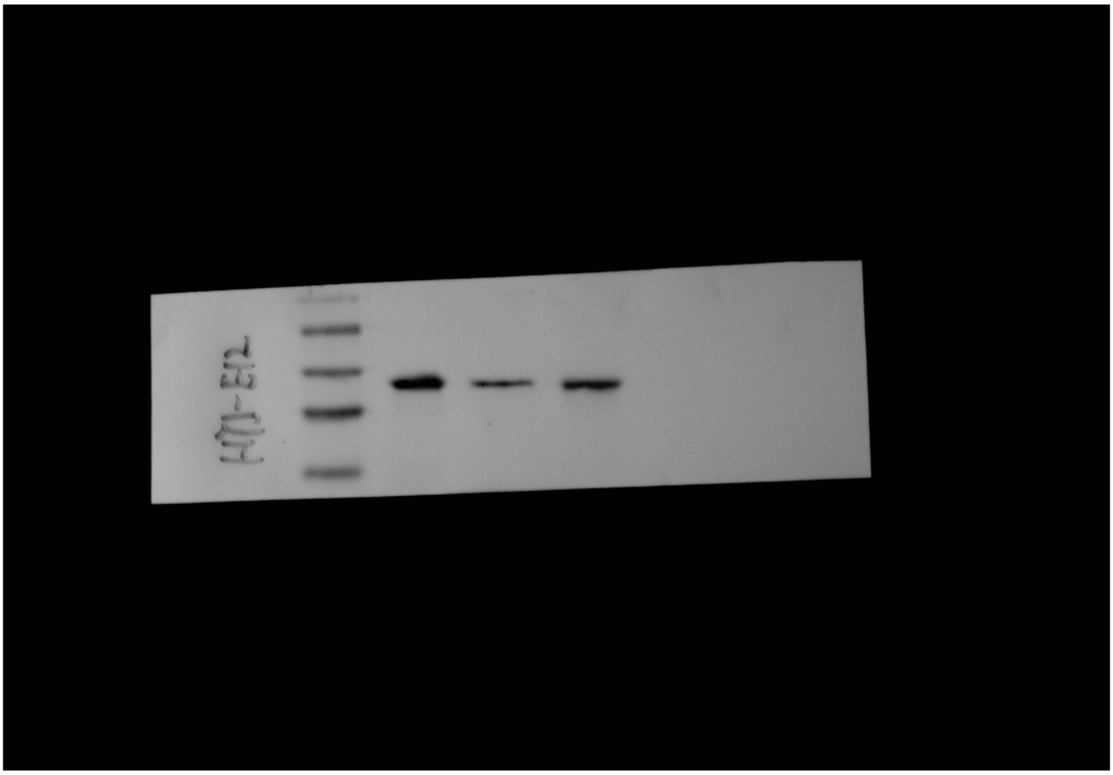

SIRT1

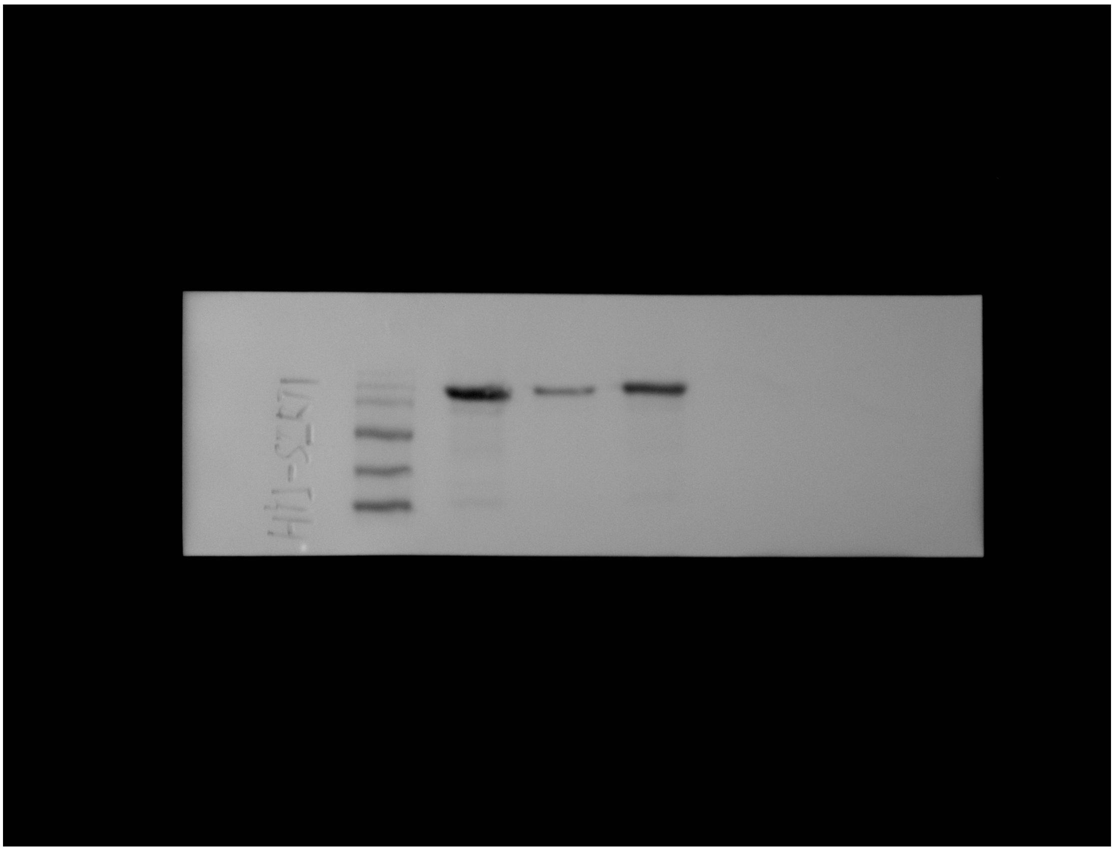

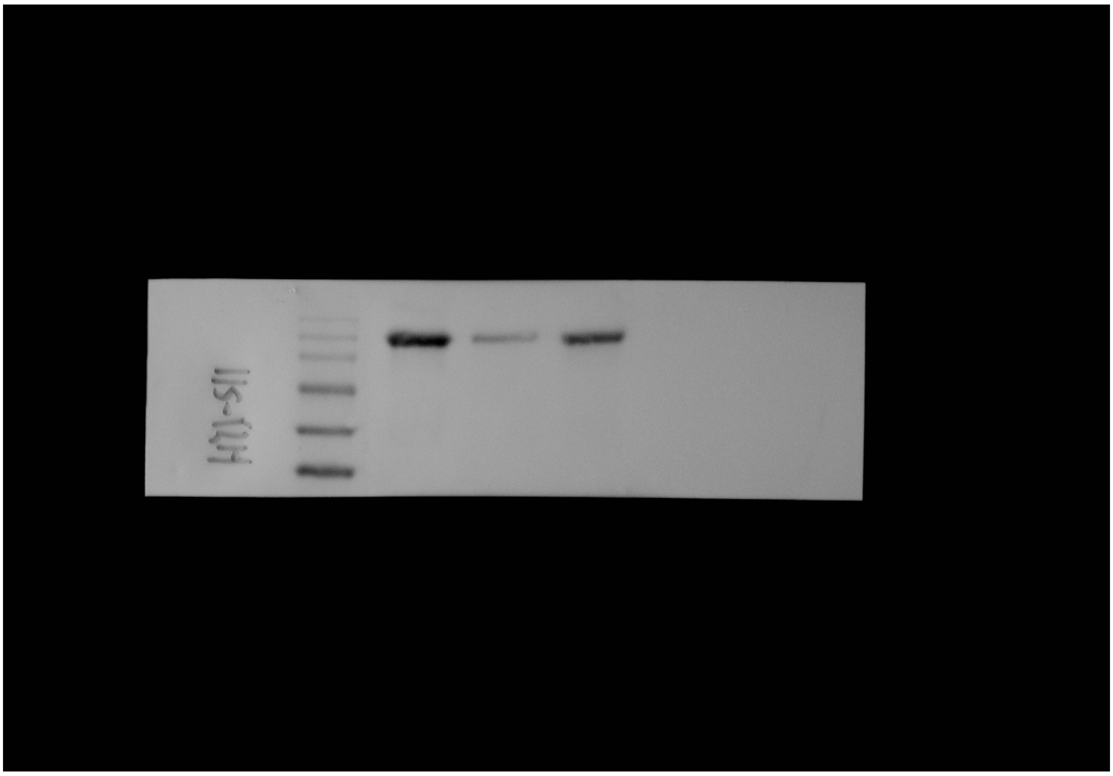

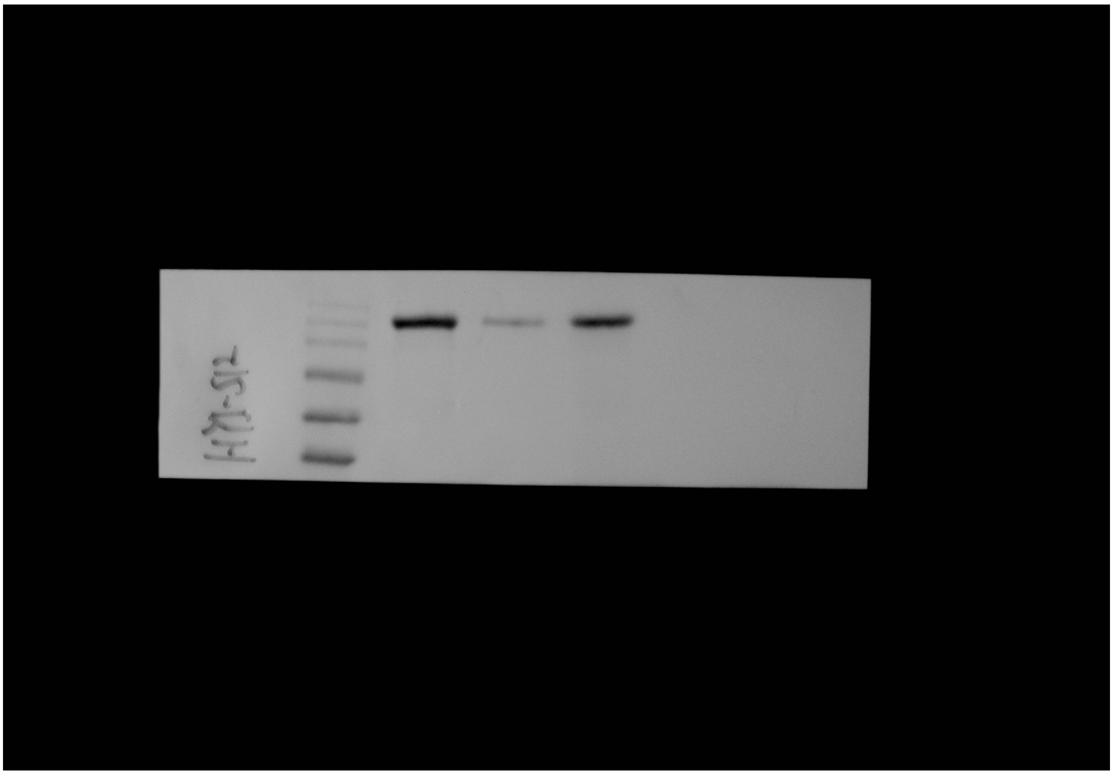

β-actin

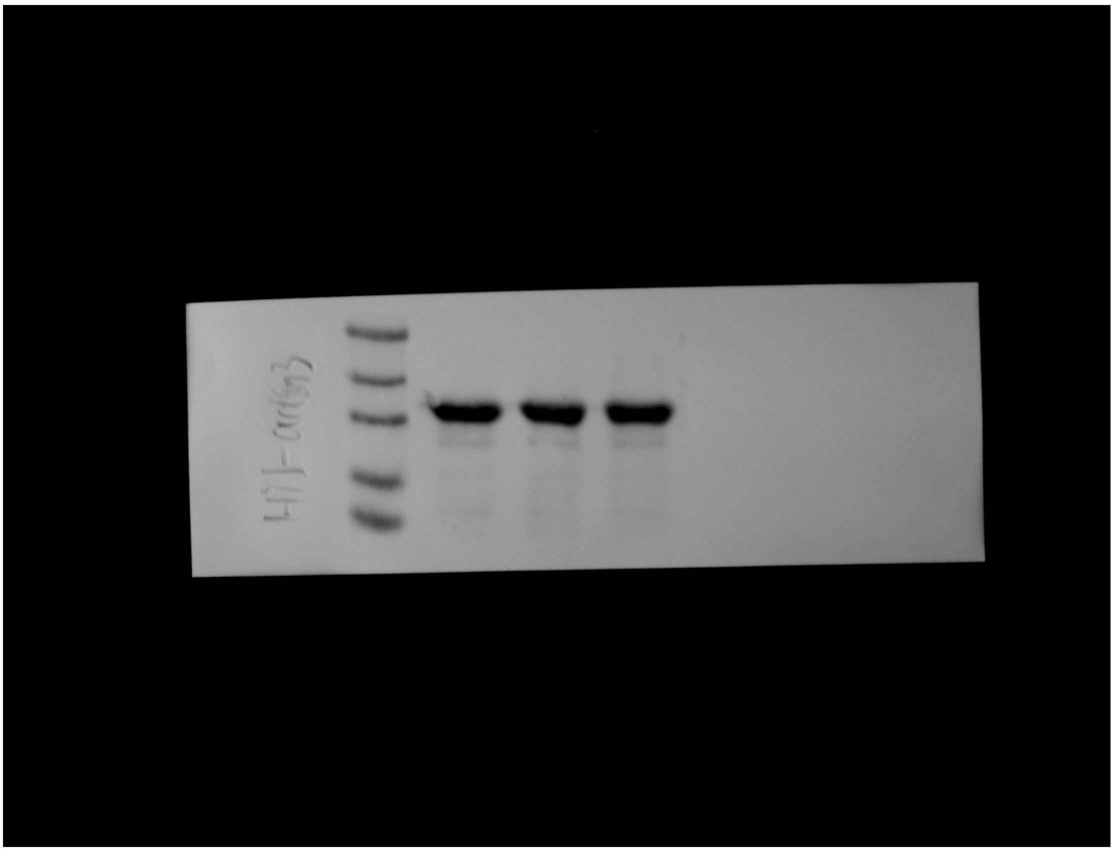

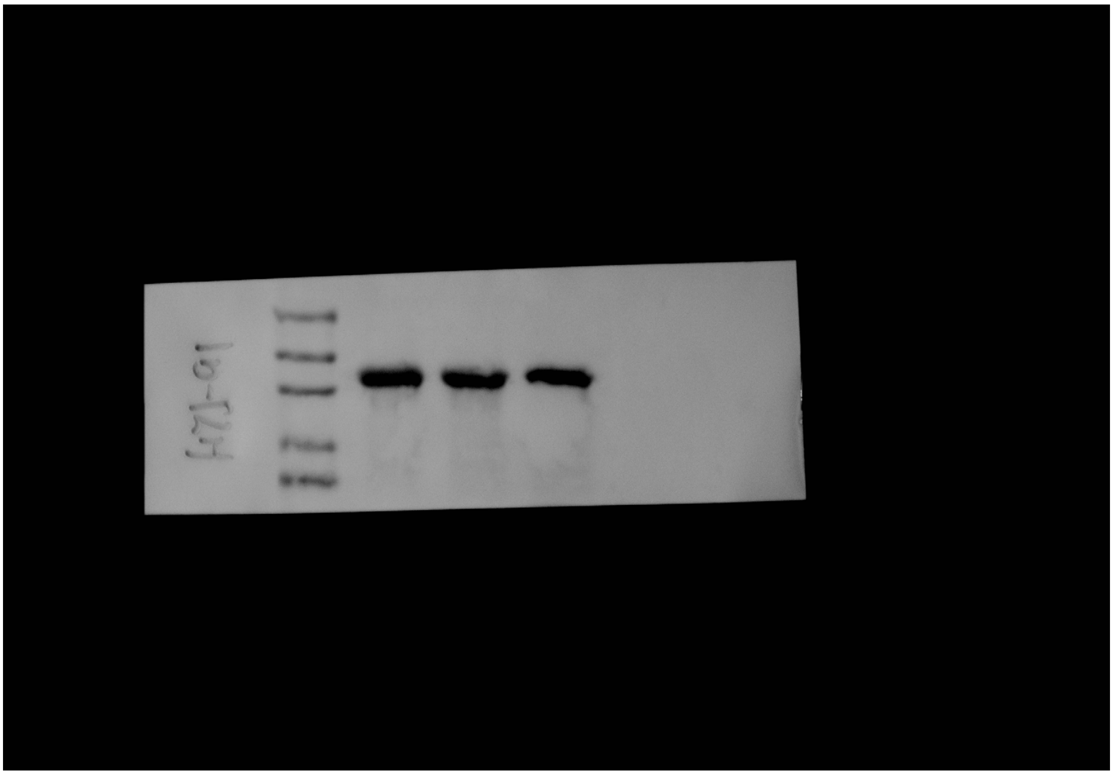

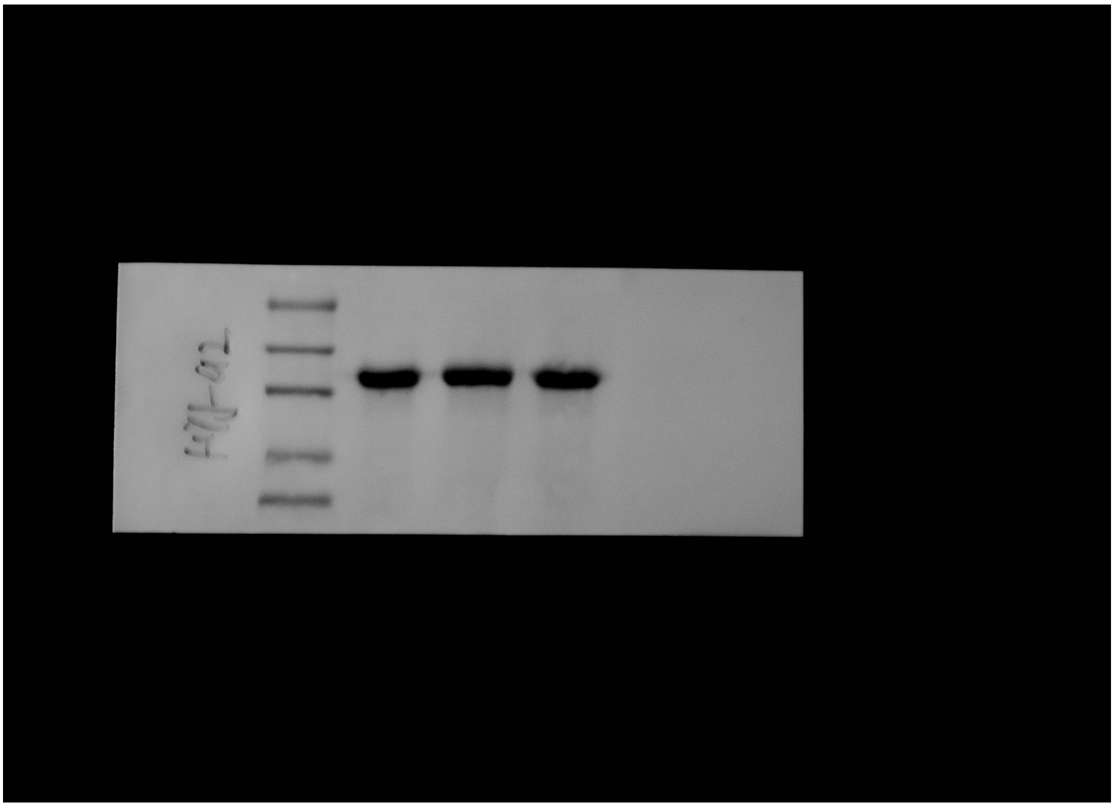

Figure 3G

GCLC

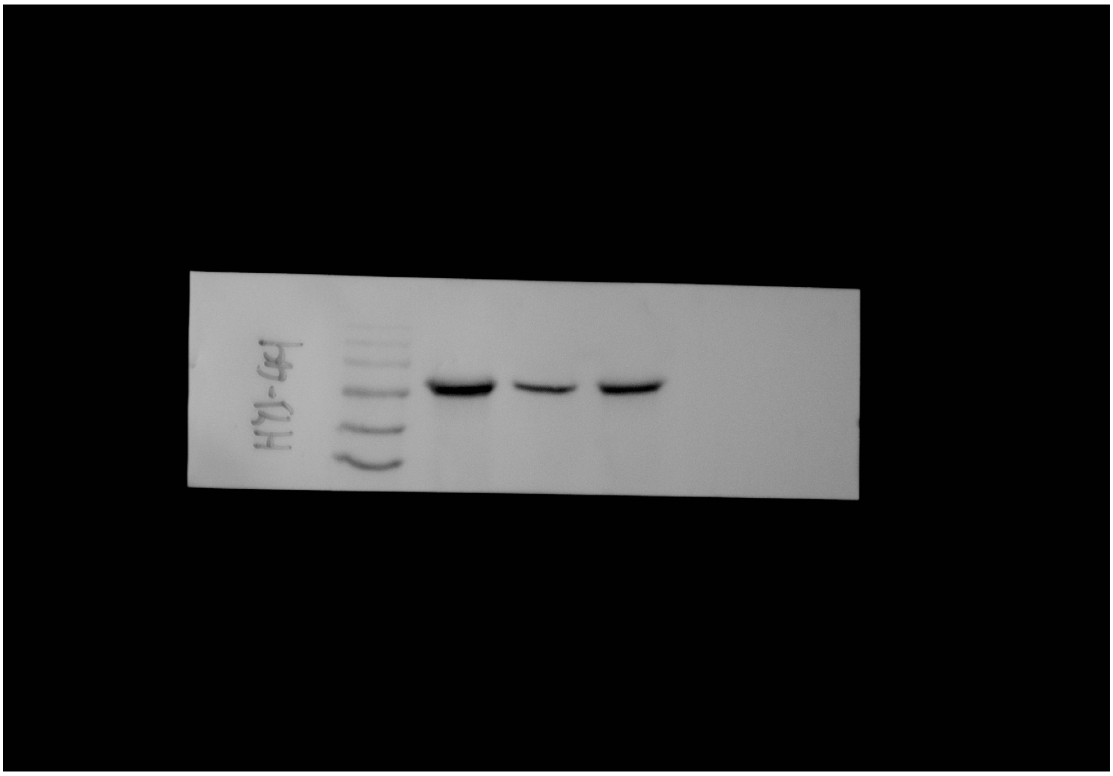

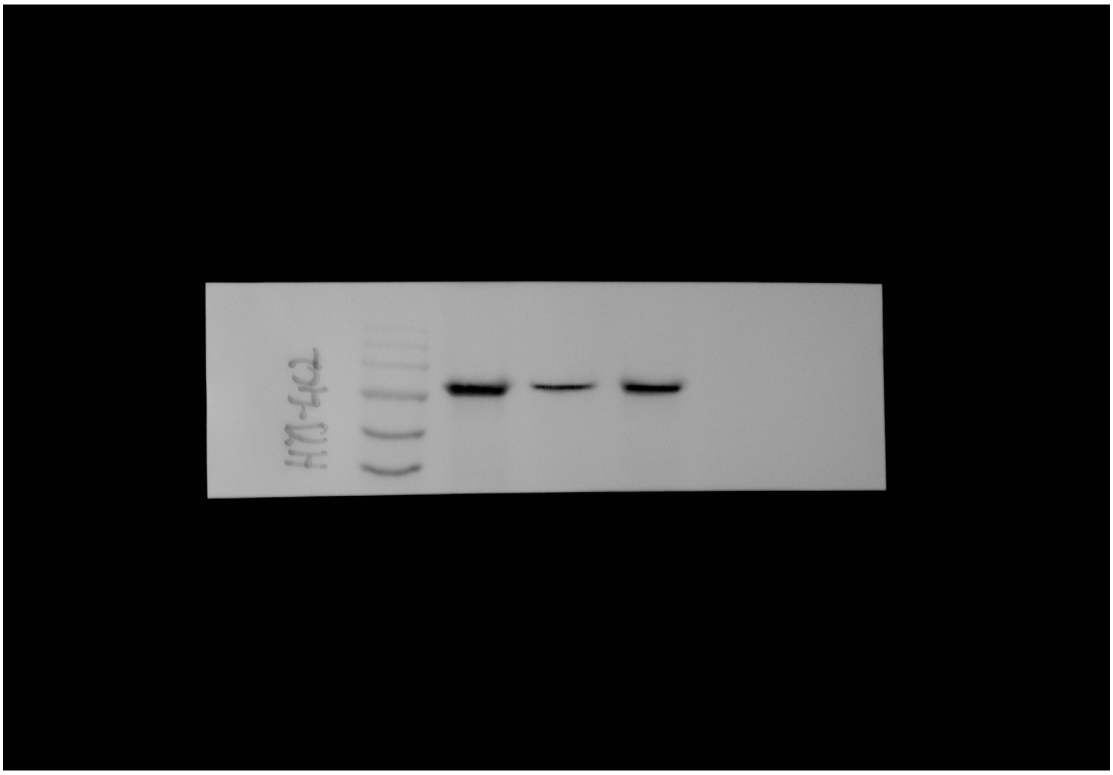

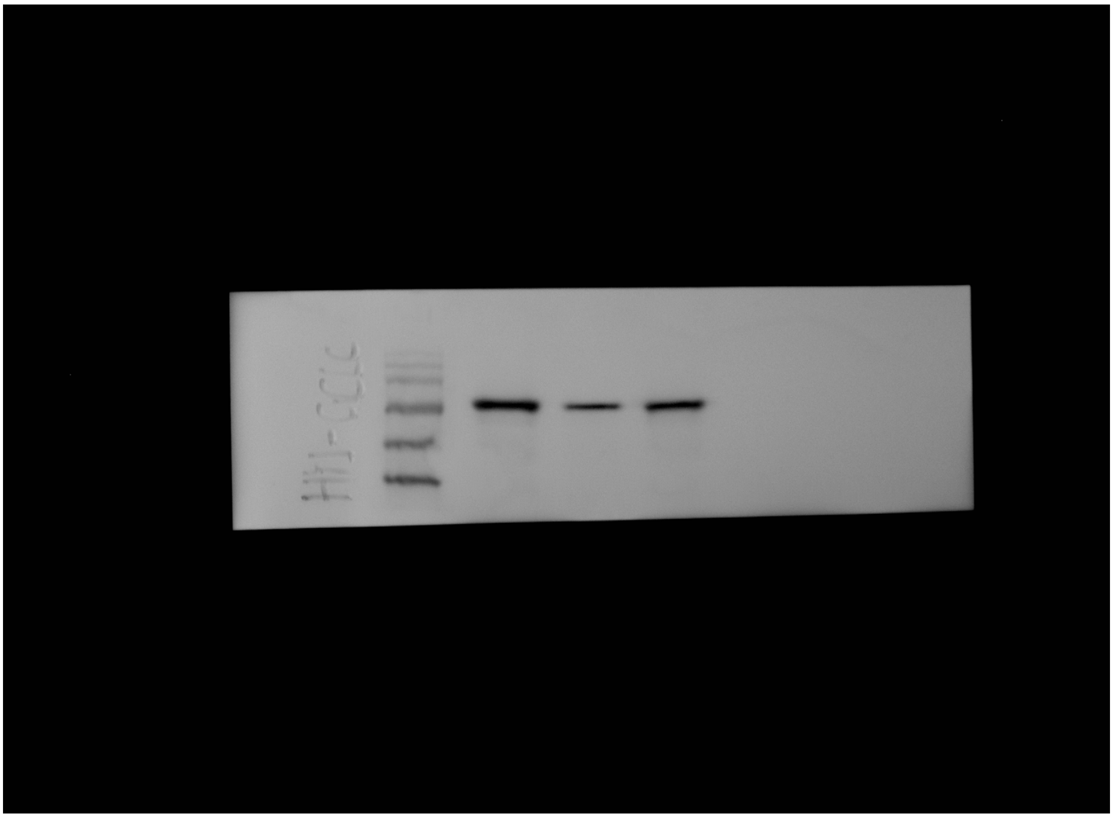

GCLM

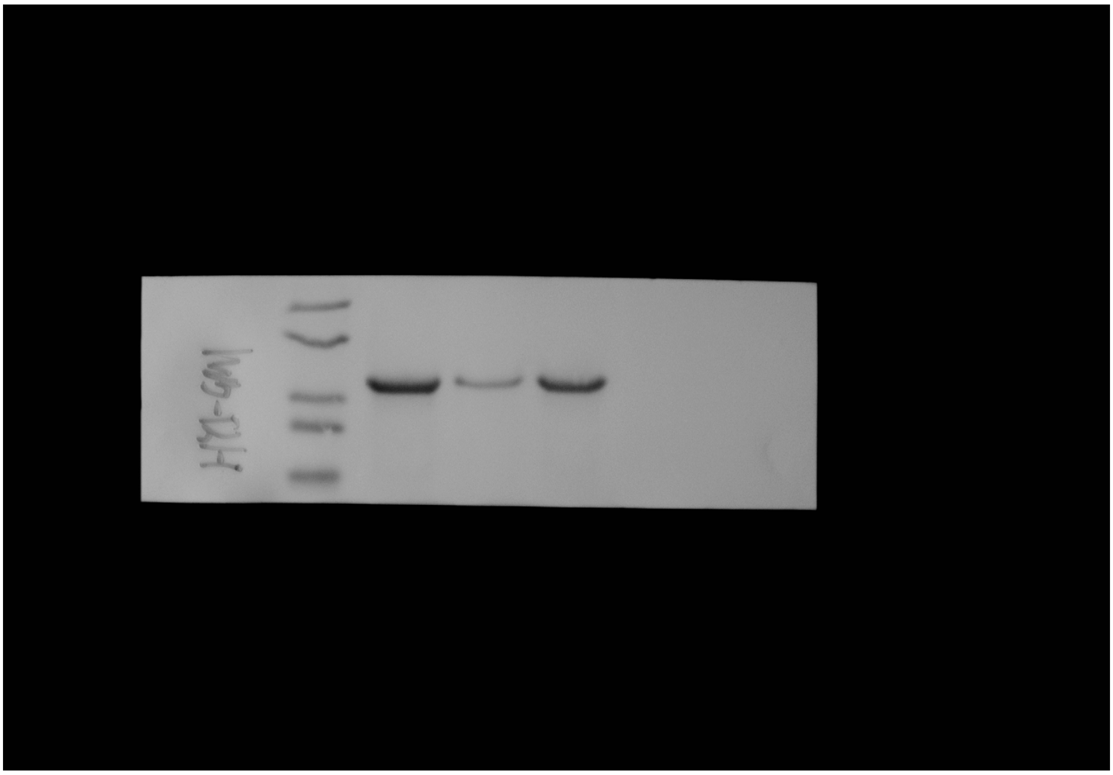

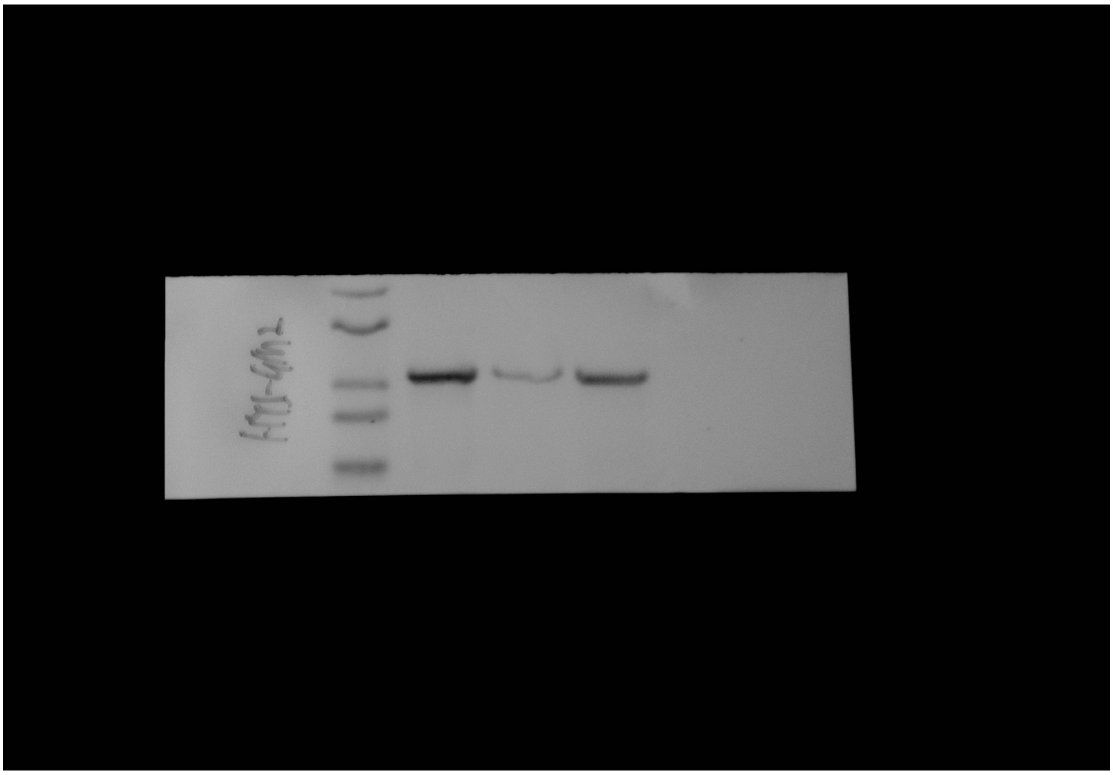

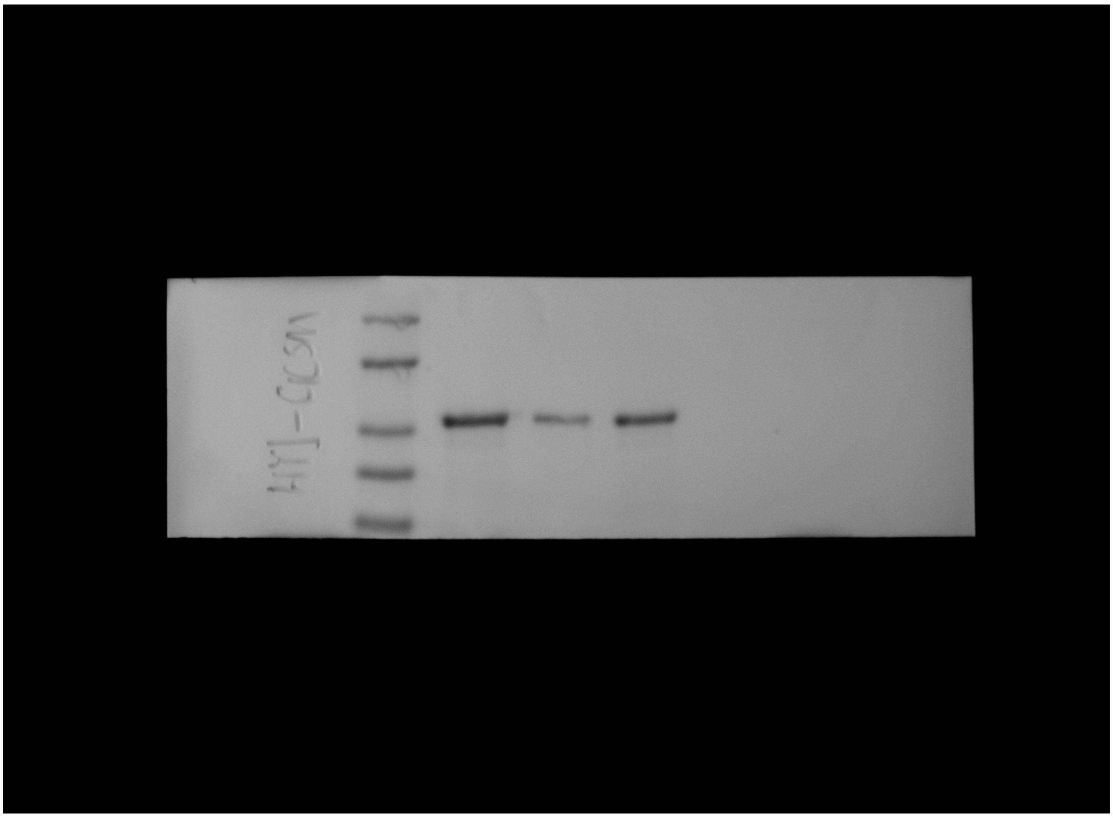

NQO1

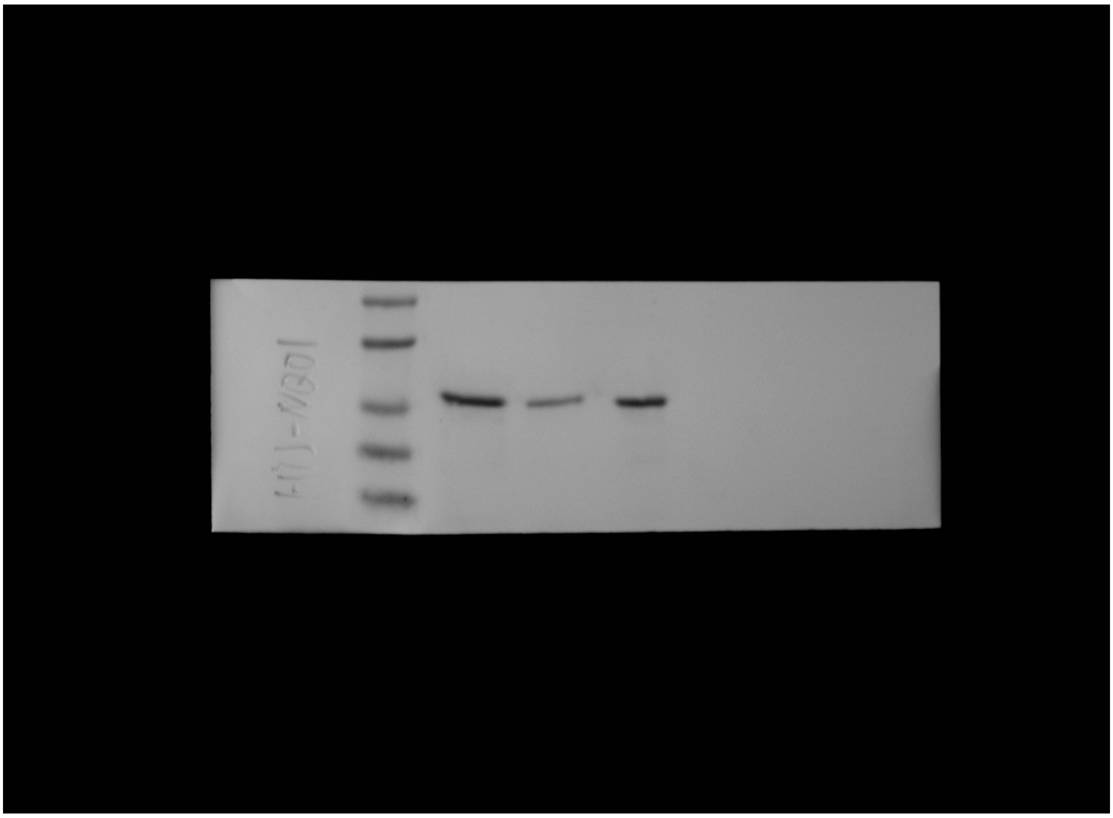

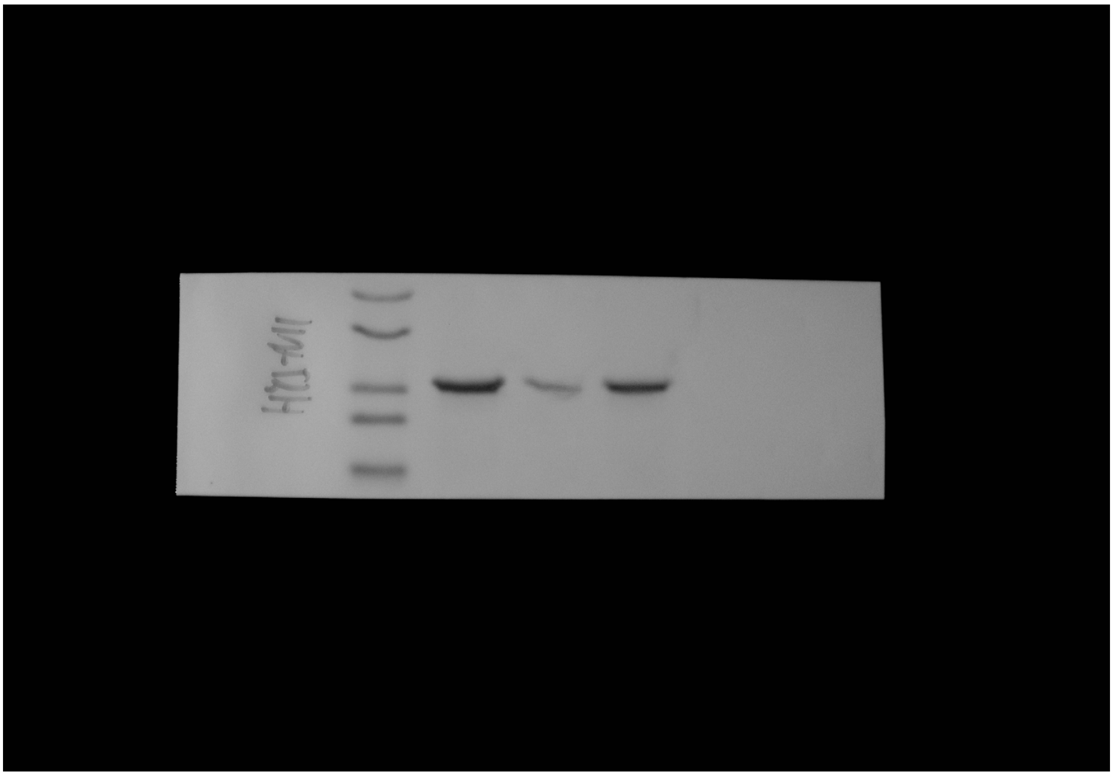

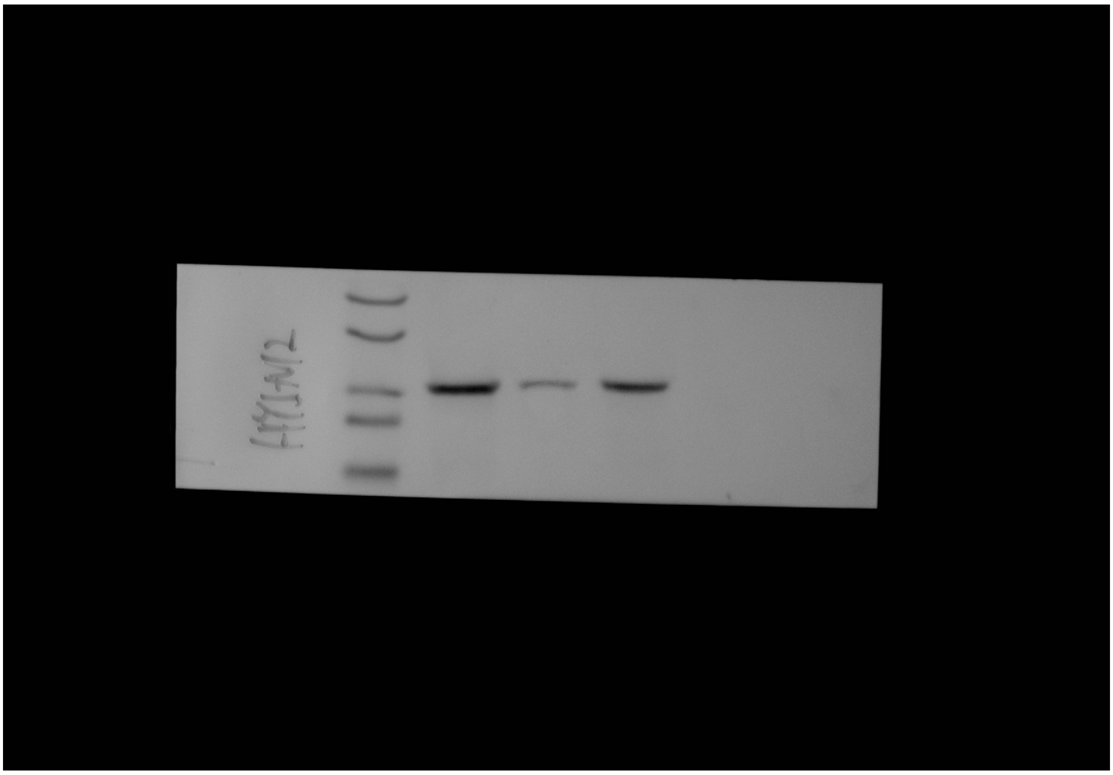

β-actin

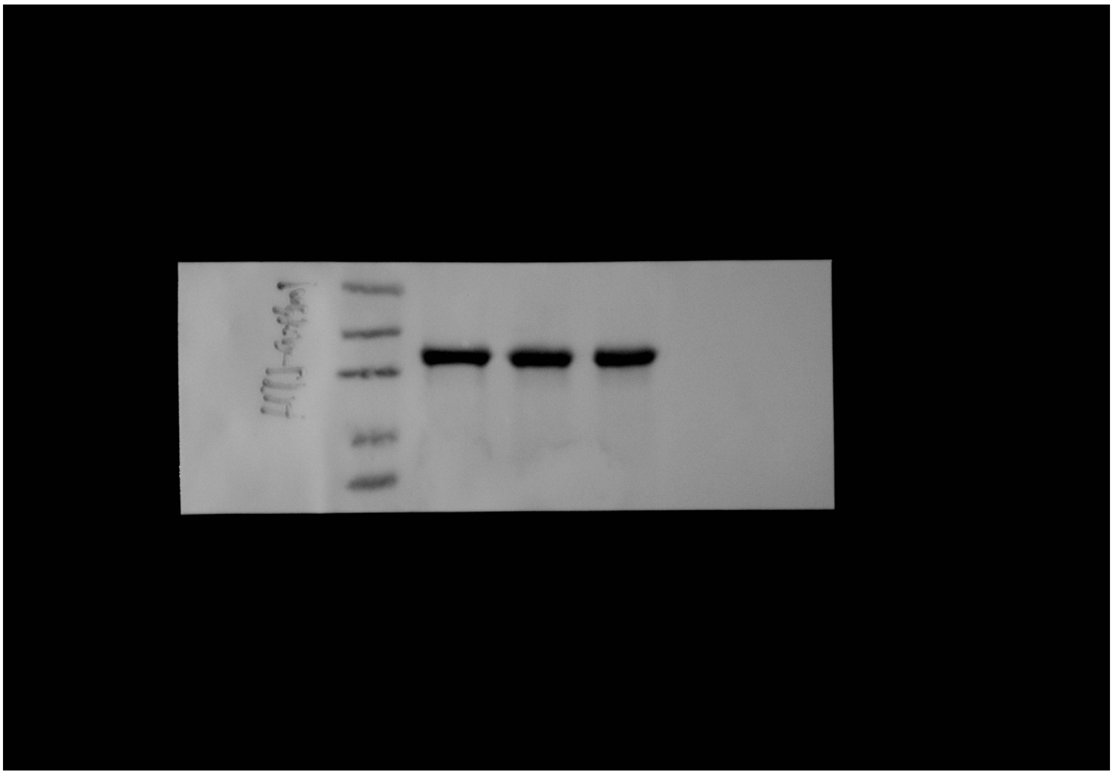

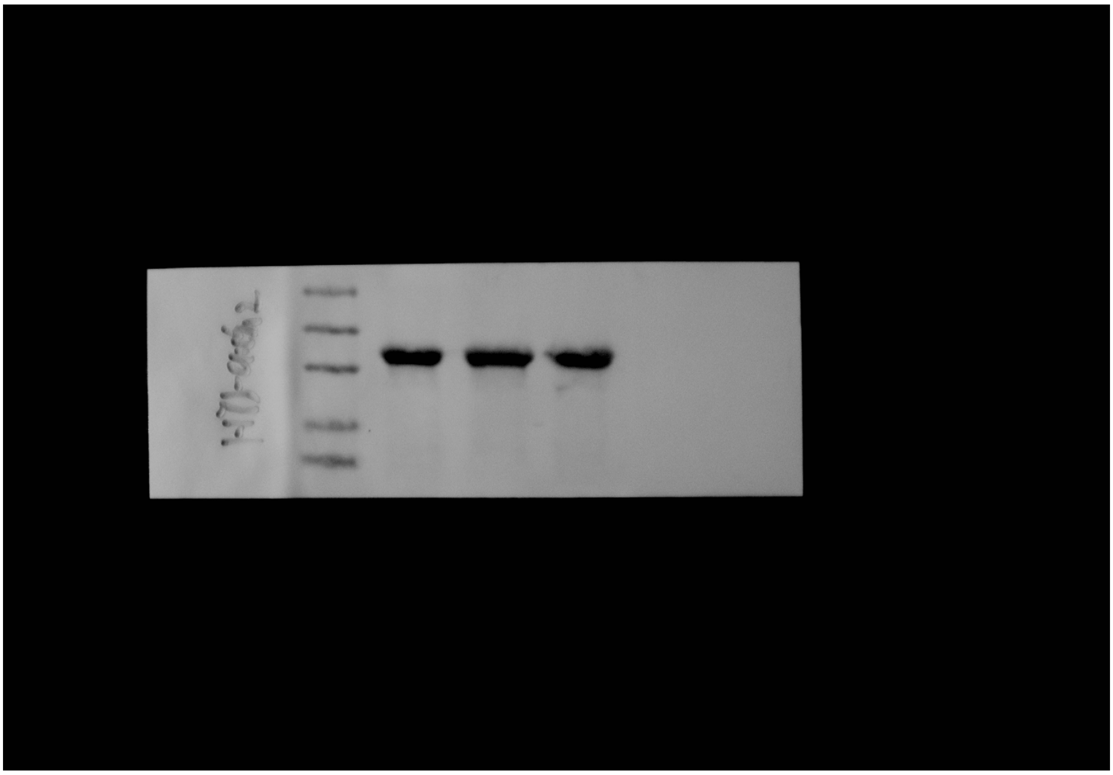

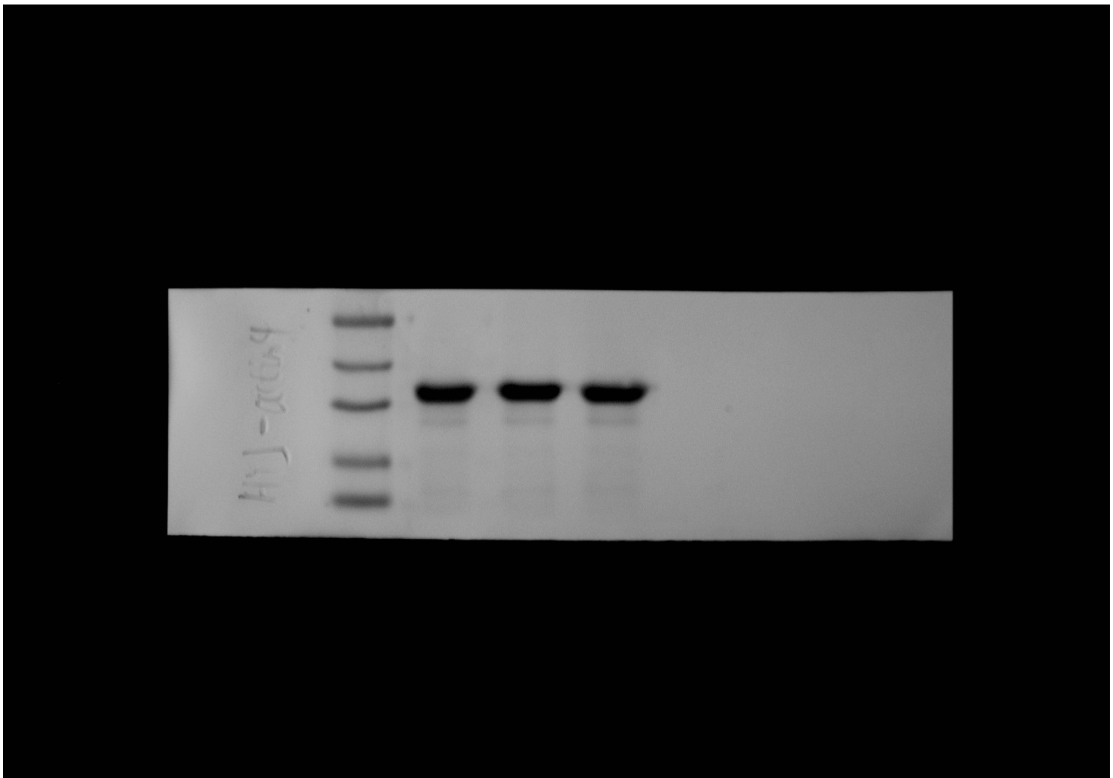

Figure 4B

BMAL1

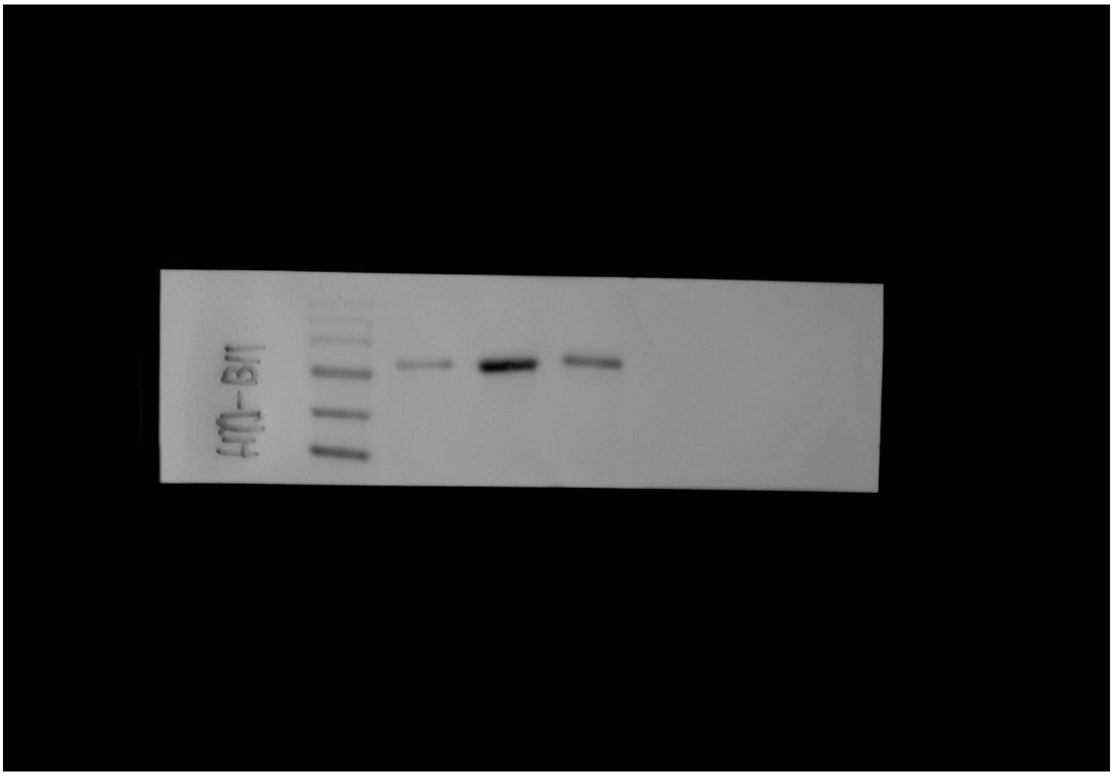

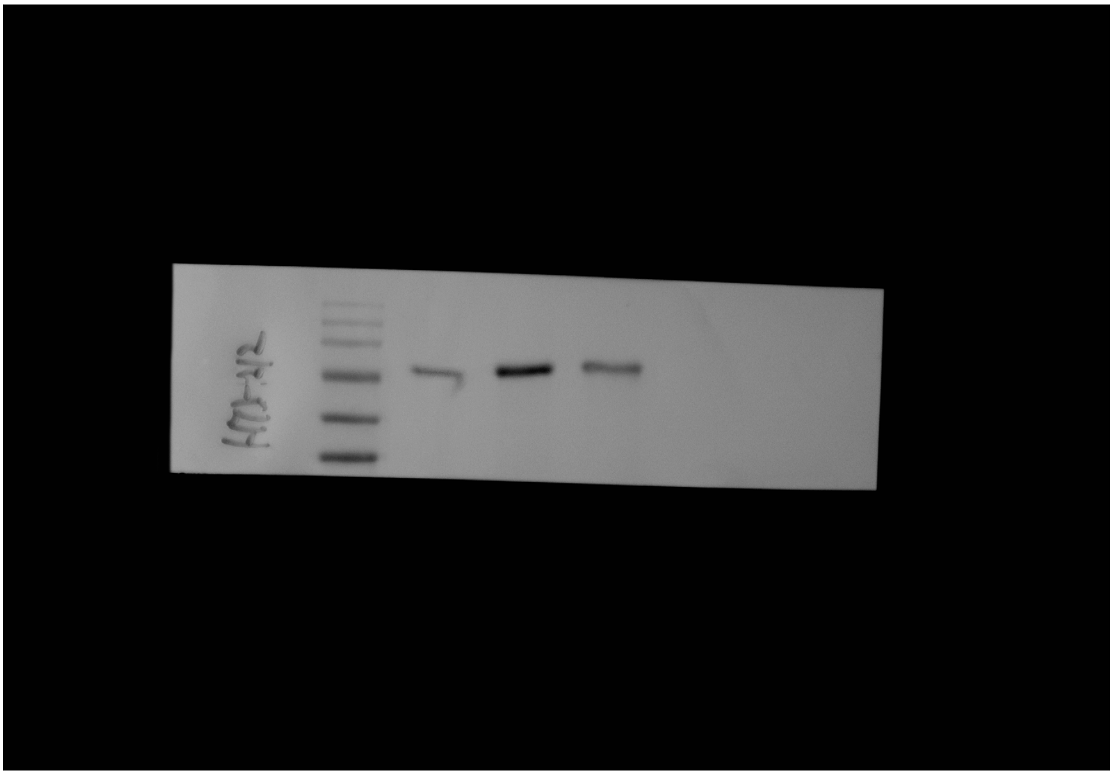

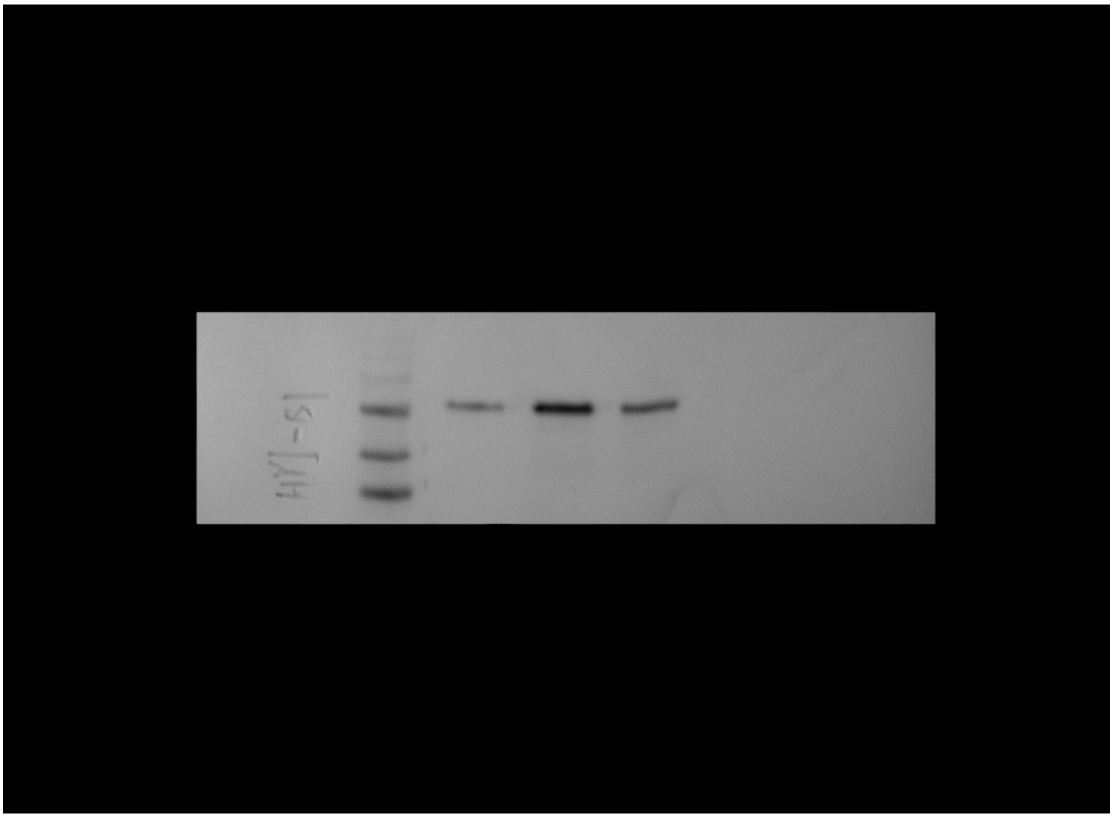

E2F1

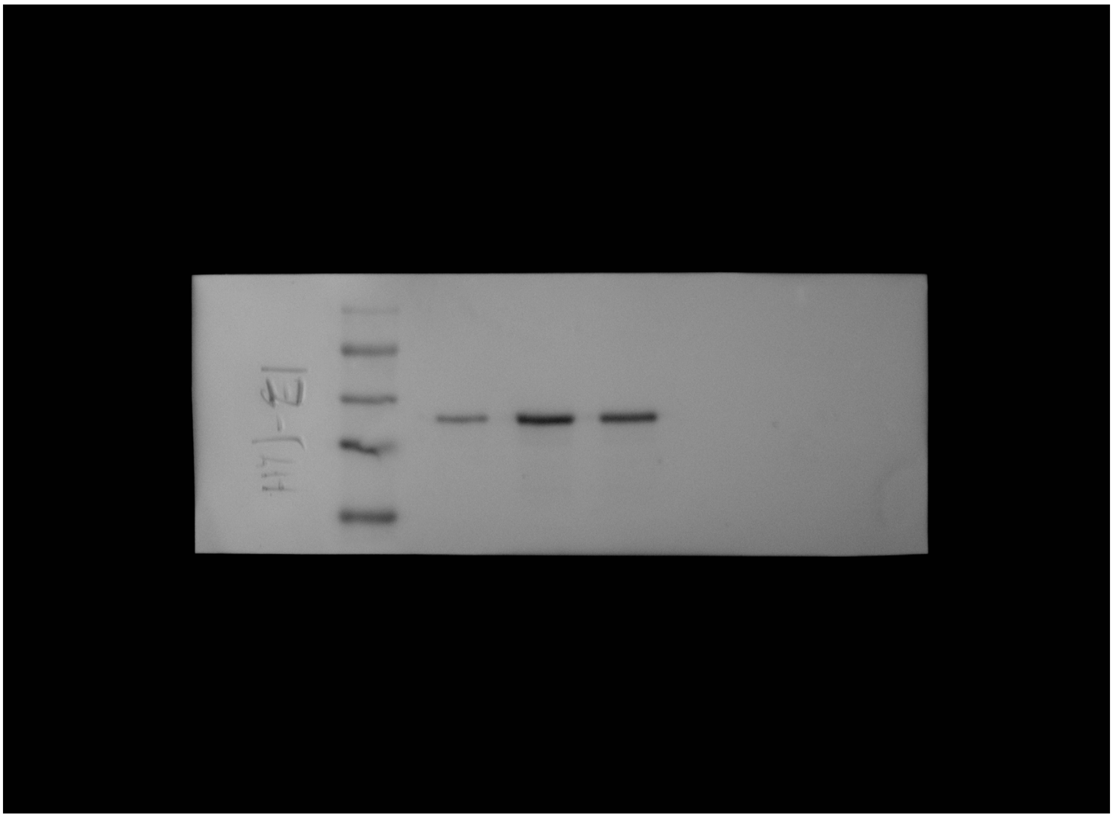

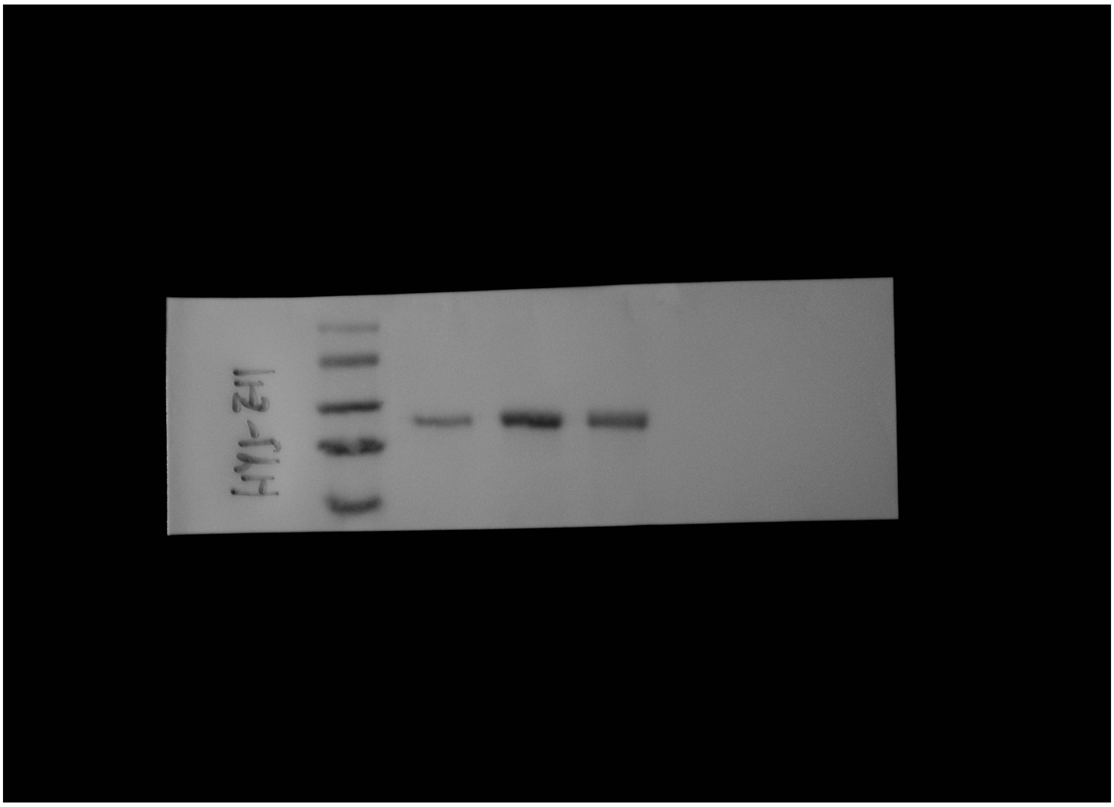

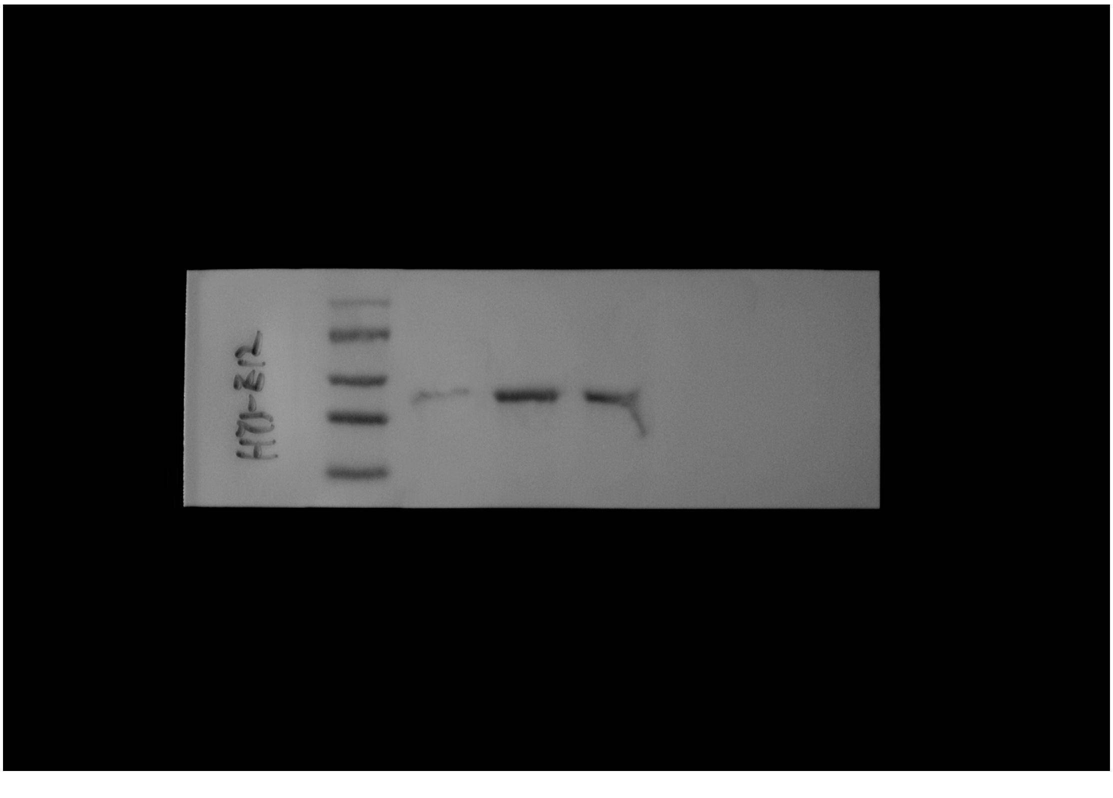

SIRT1

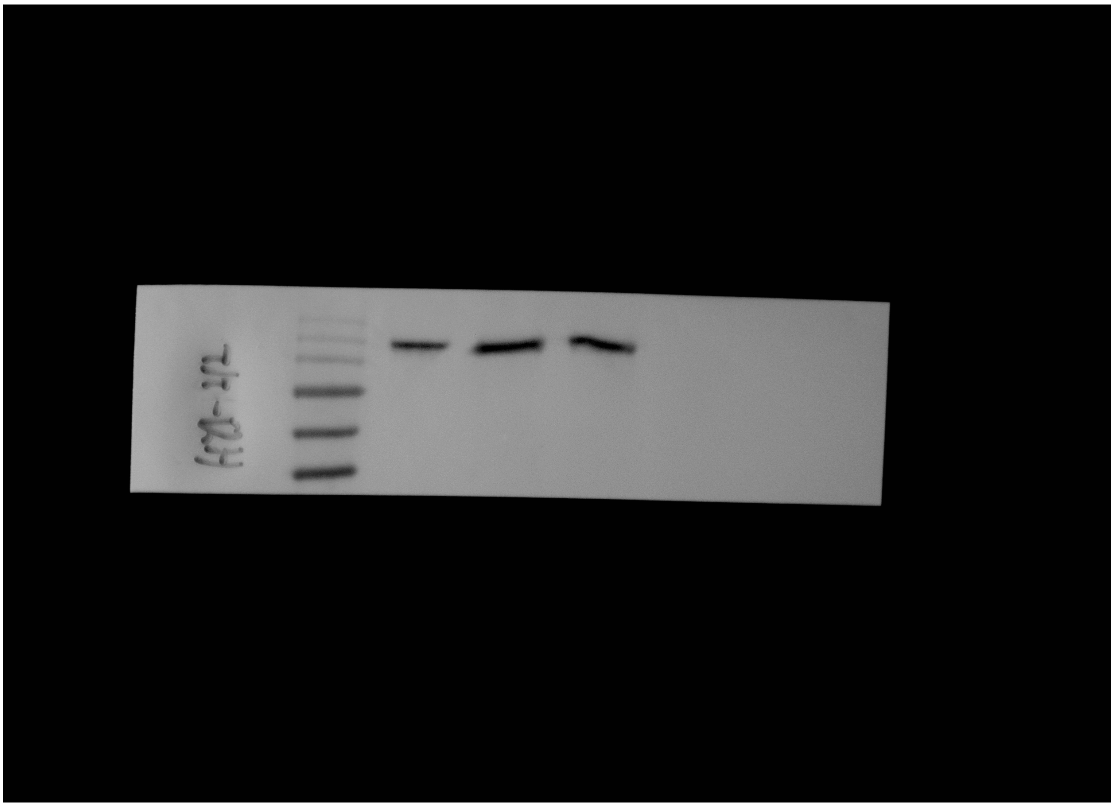

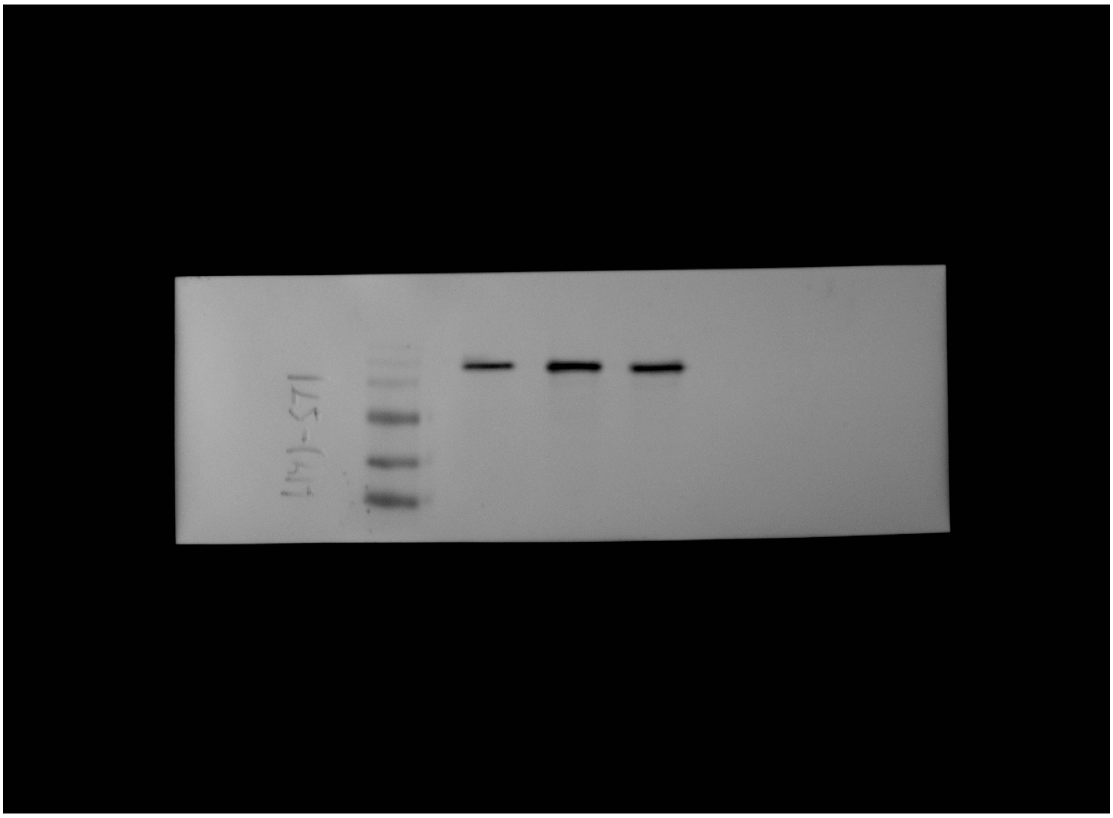

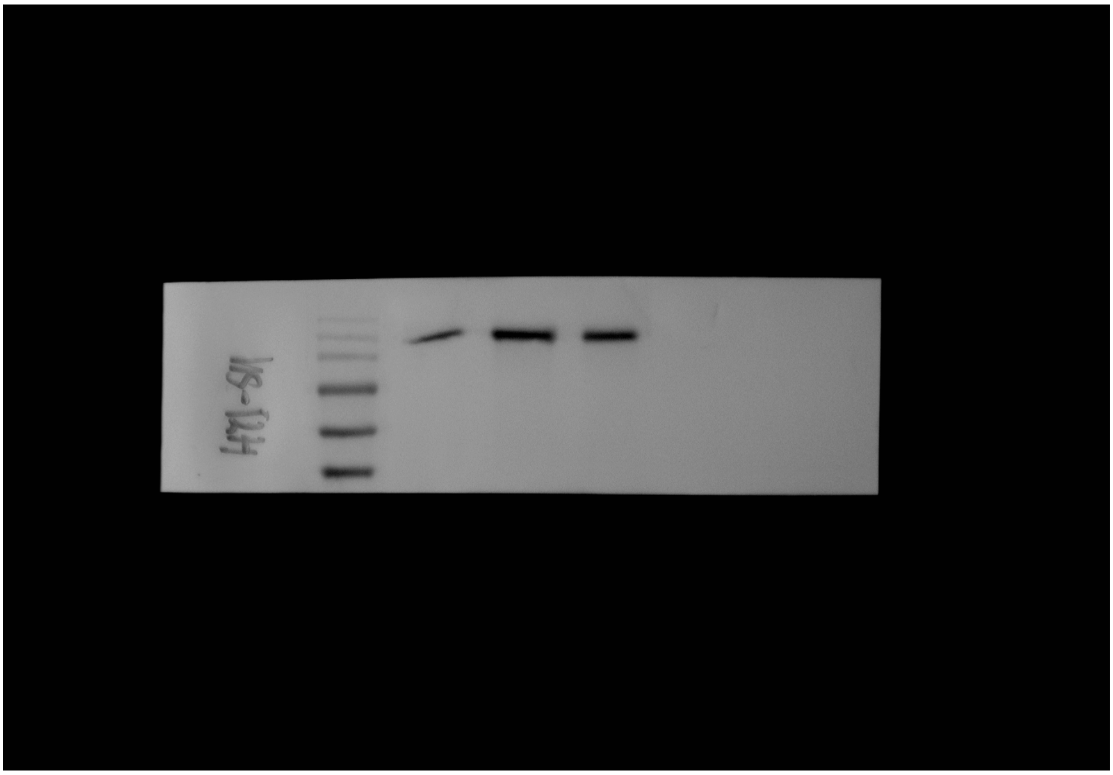

β-actin

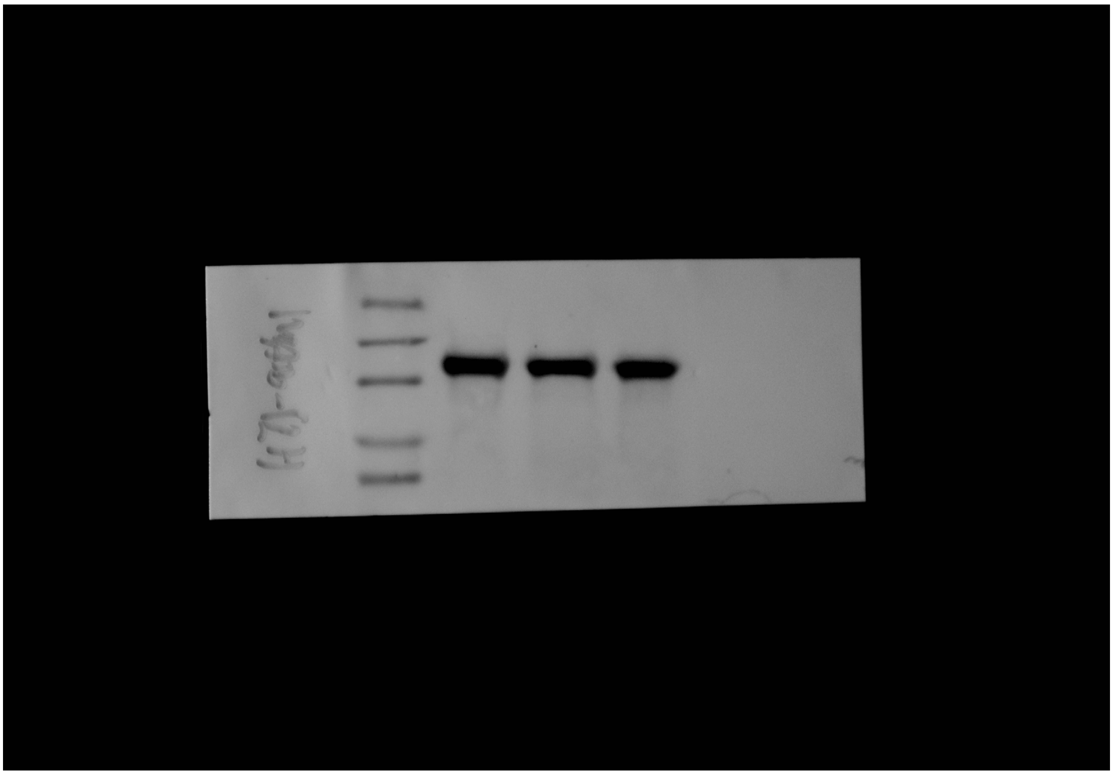

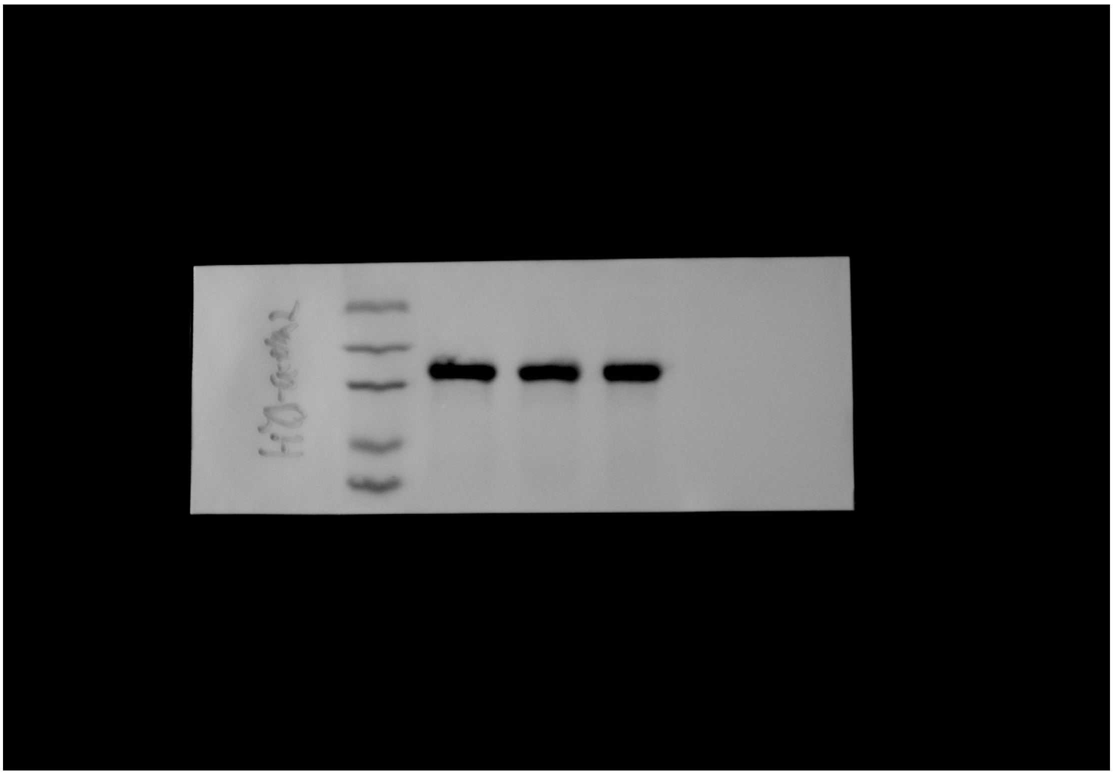

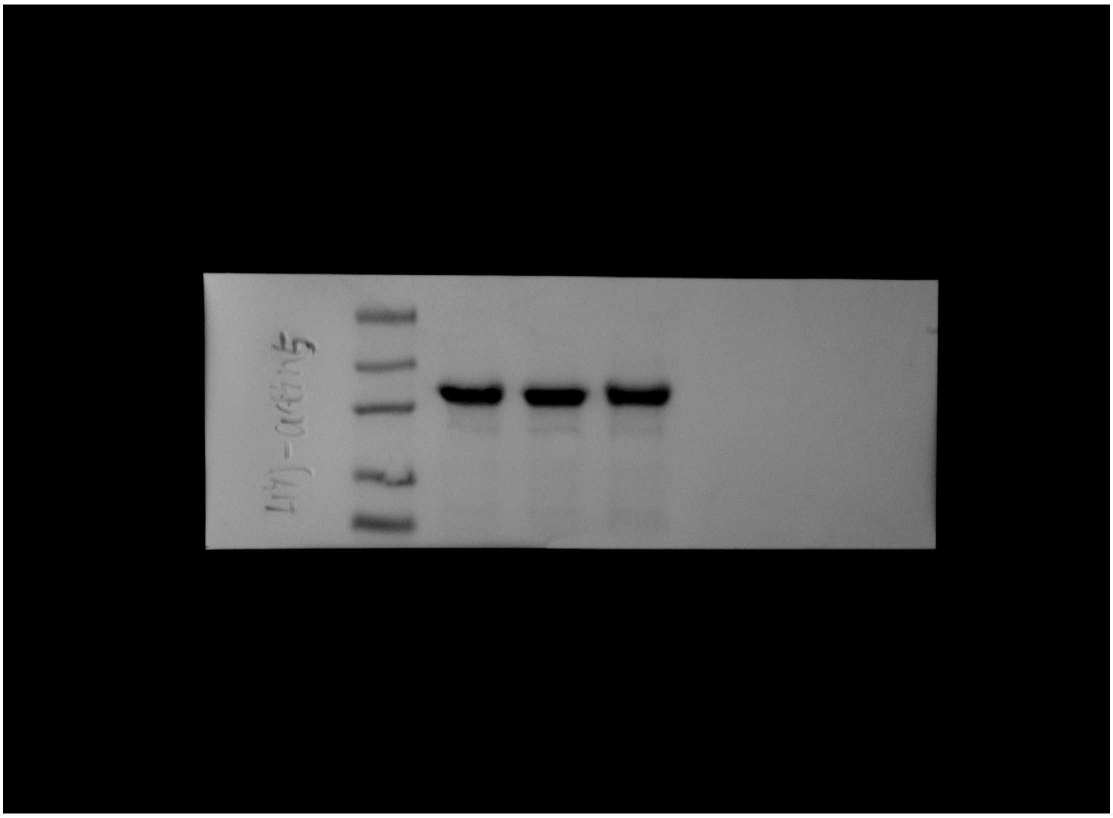

Figure 4D

GCLC

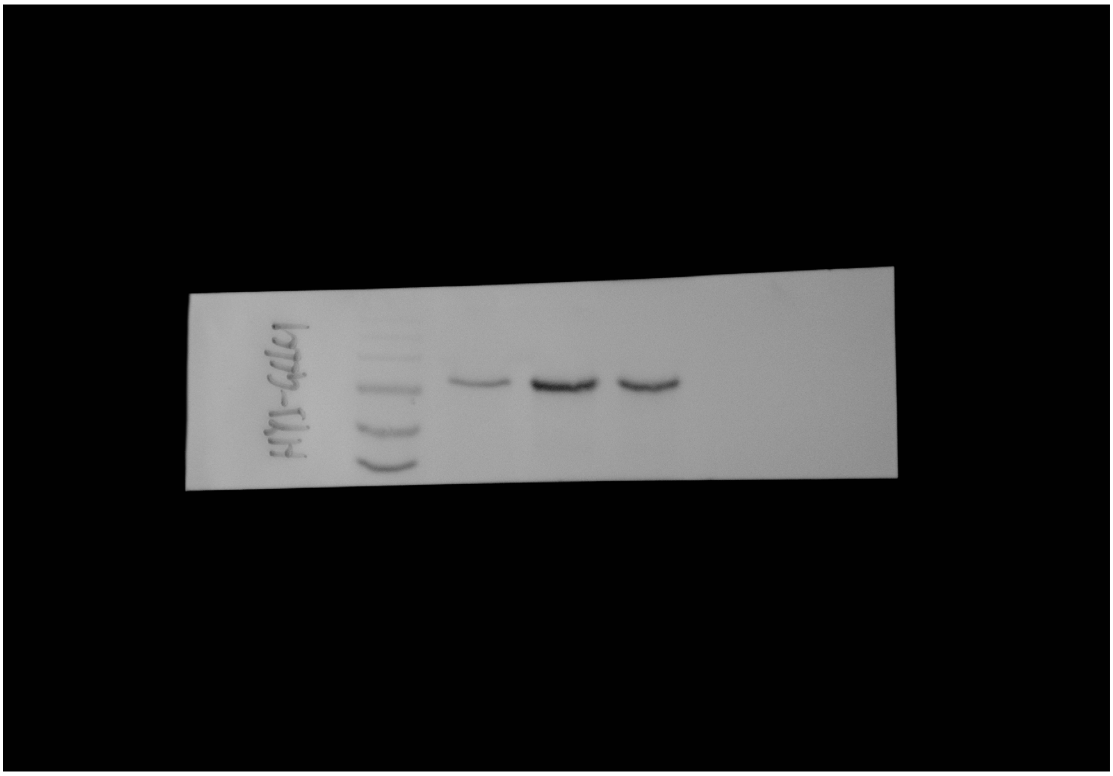

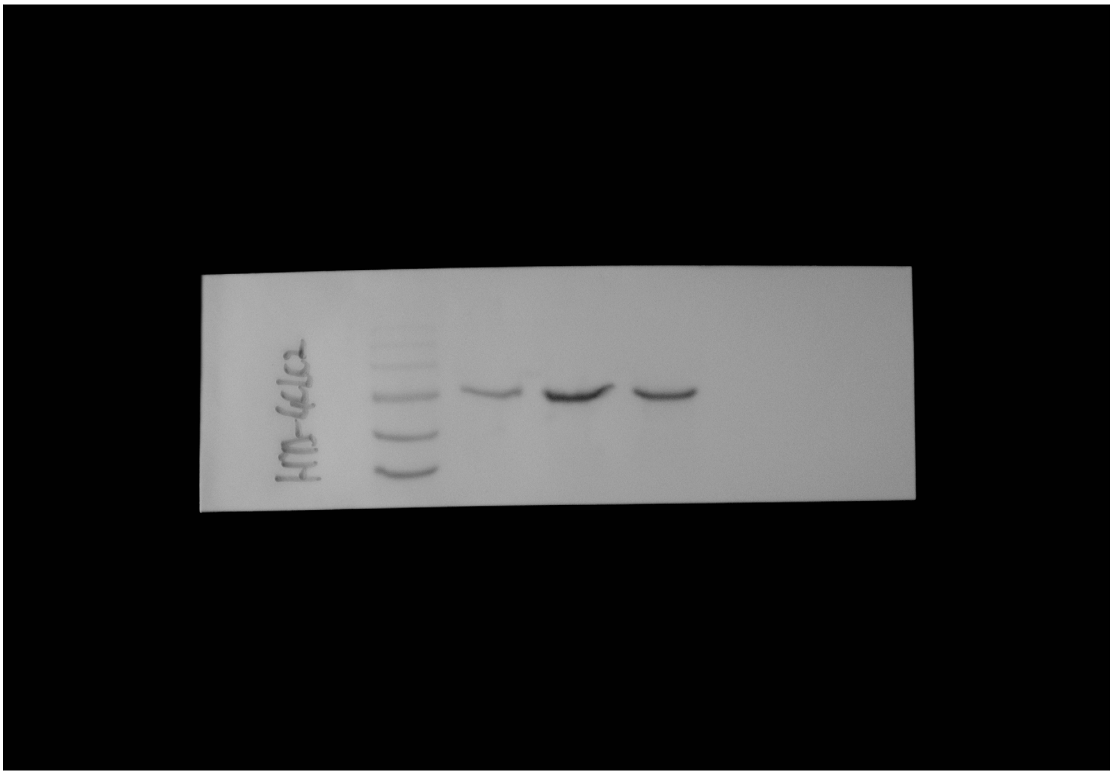

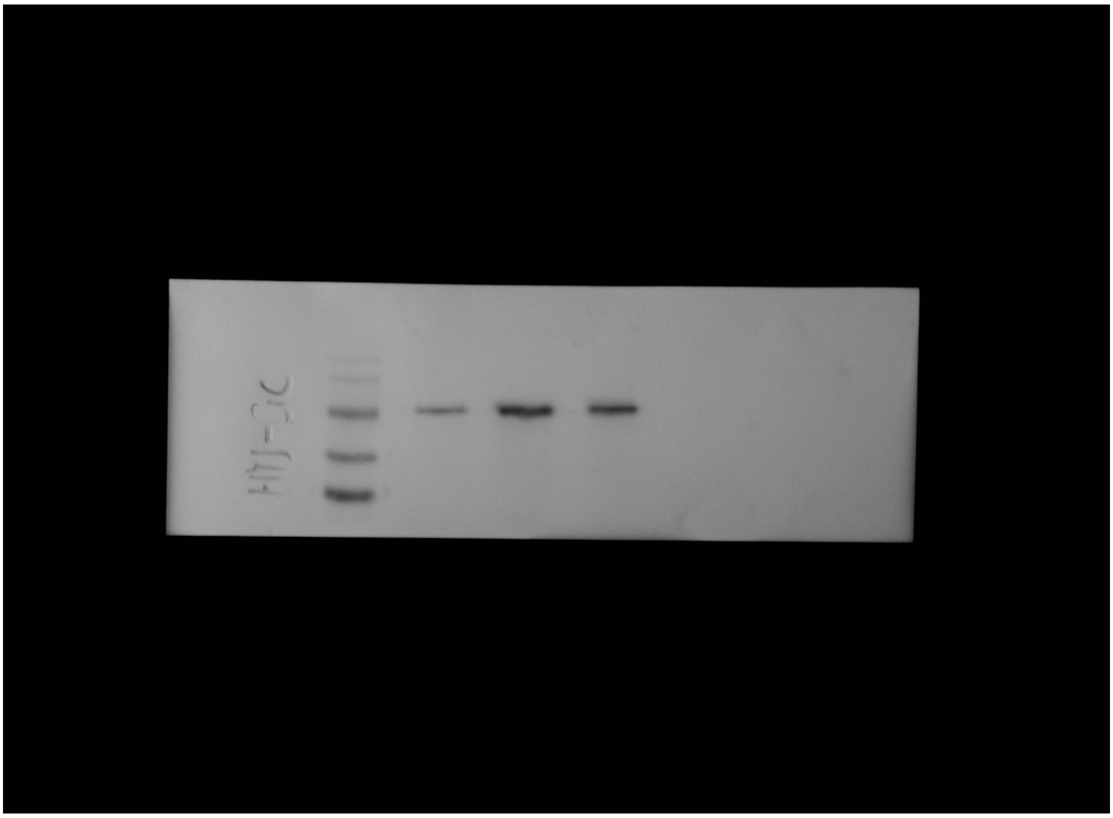

GCLM

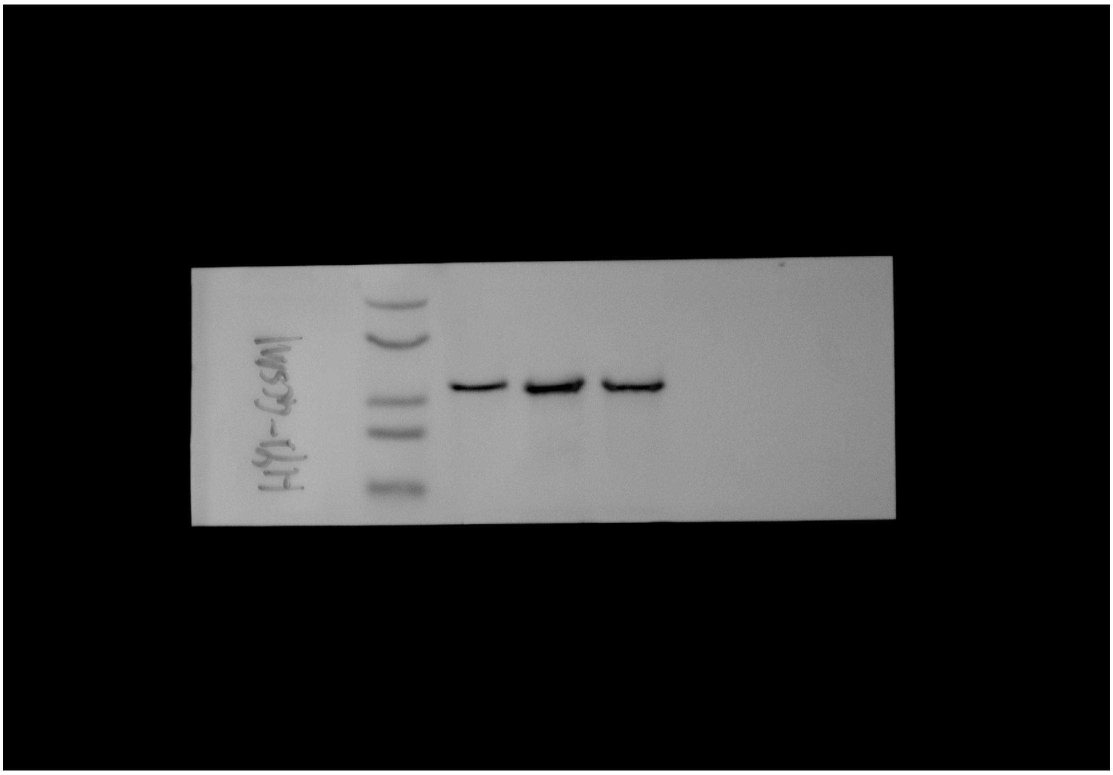

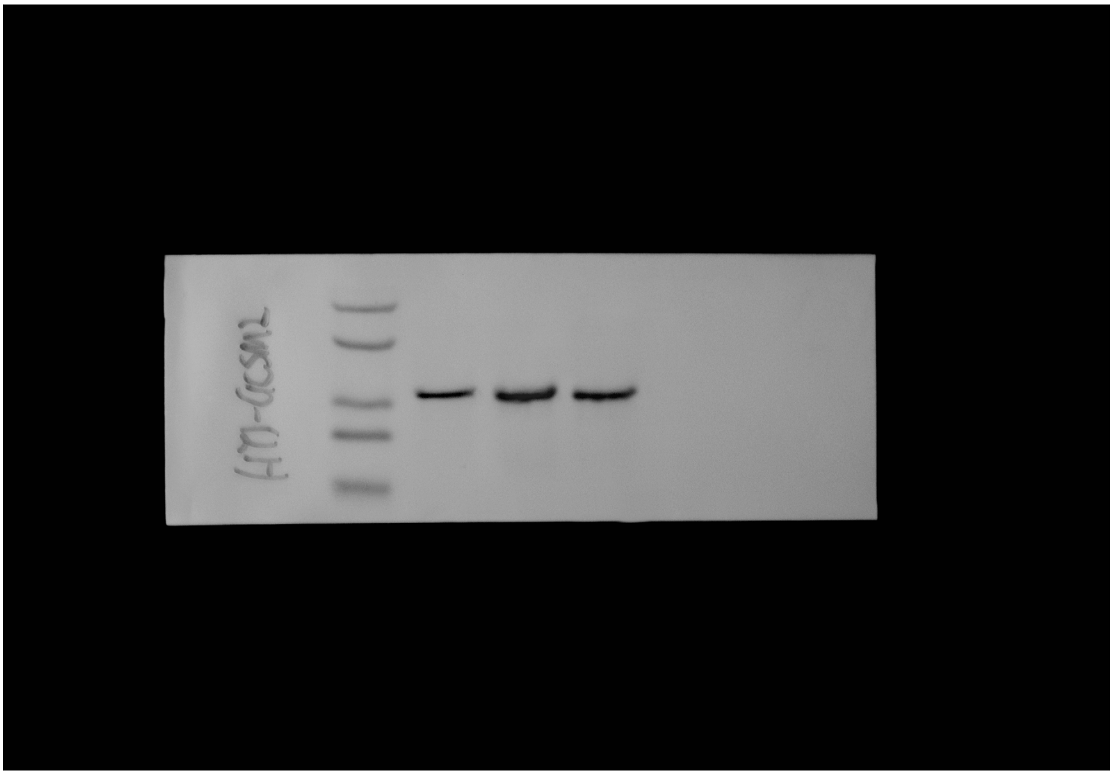

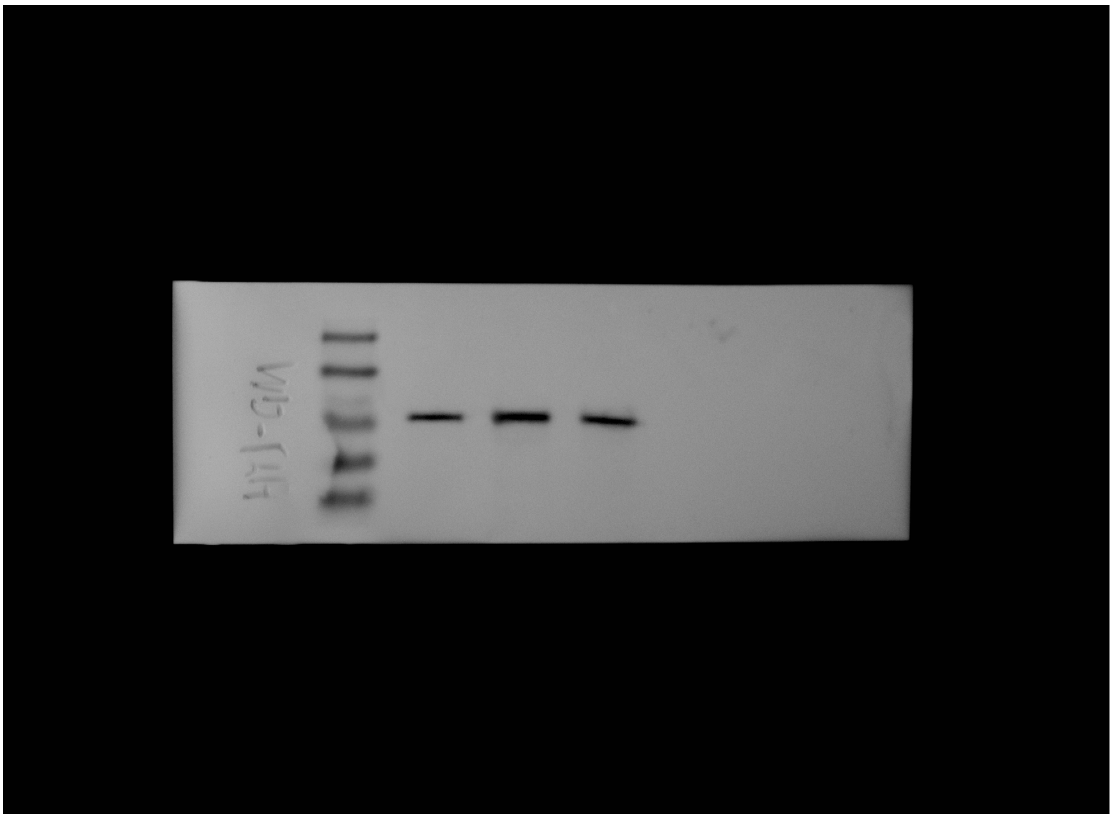

NQO1

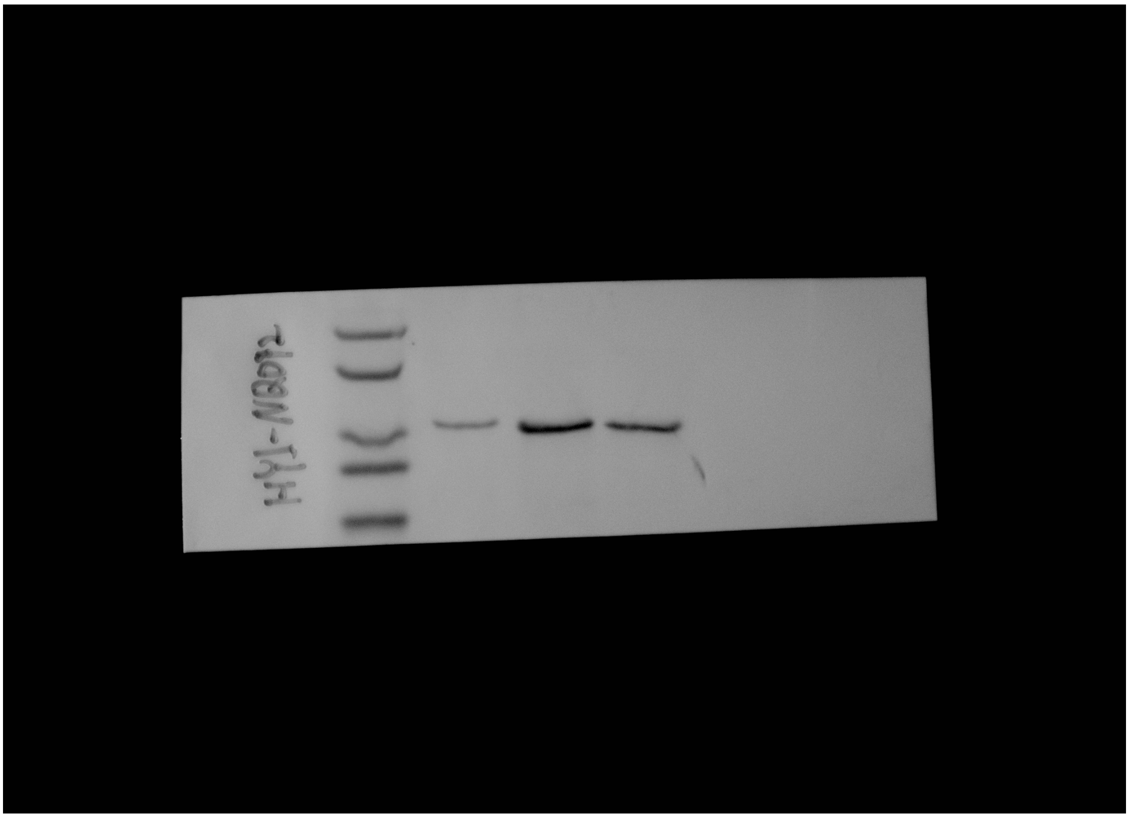
HYI-NB012

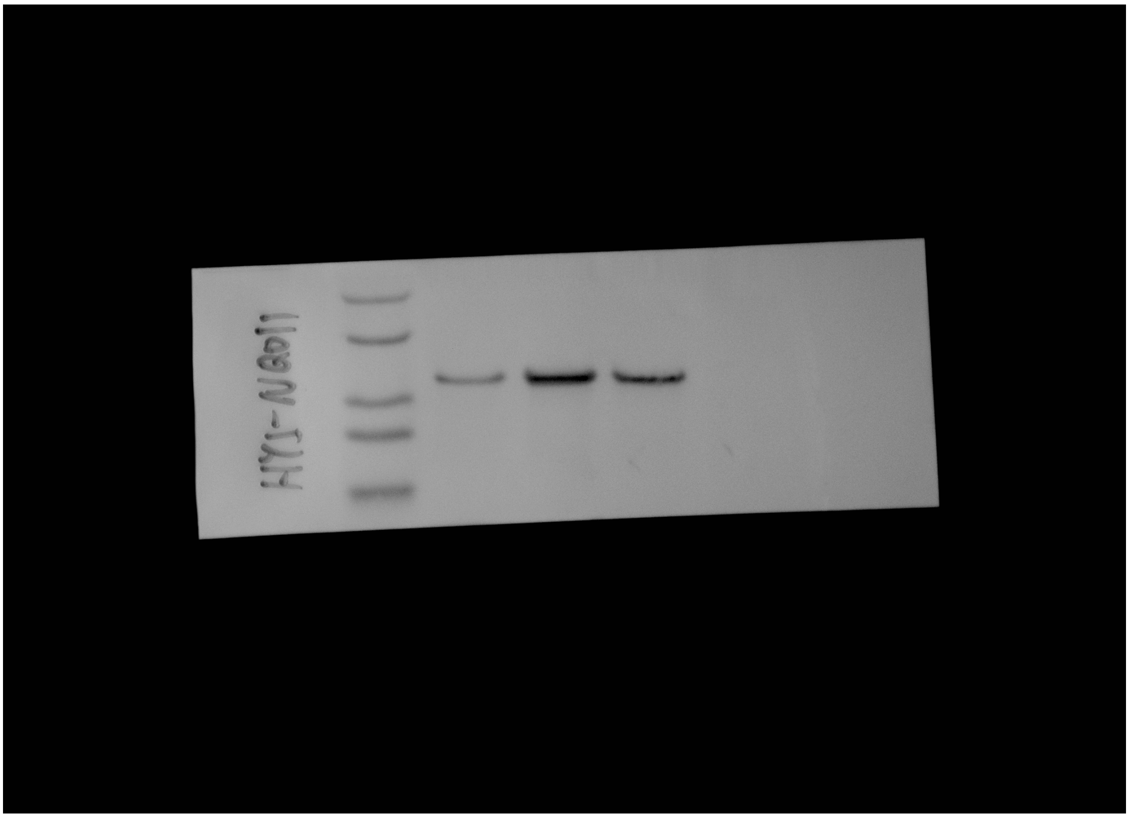
HYI-NB011

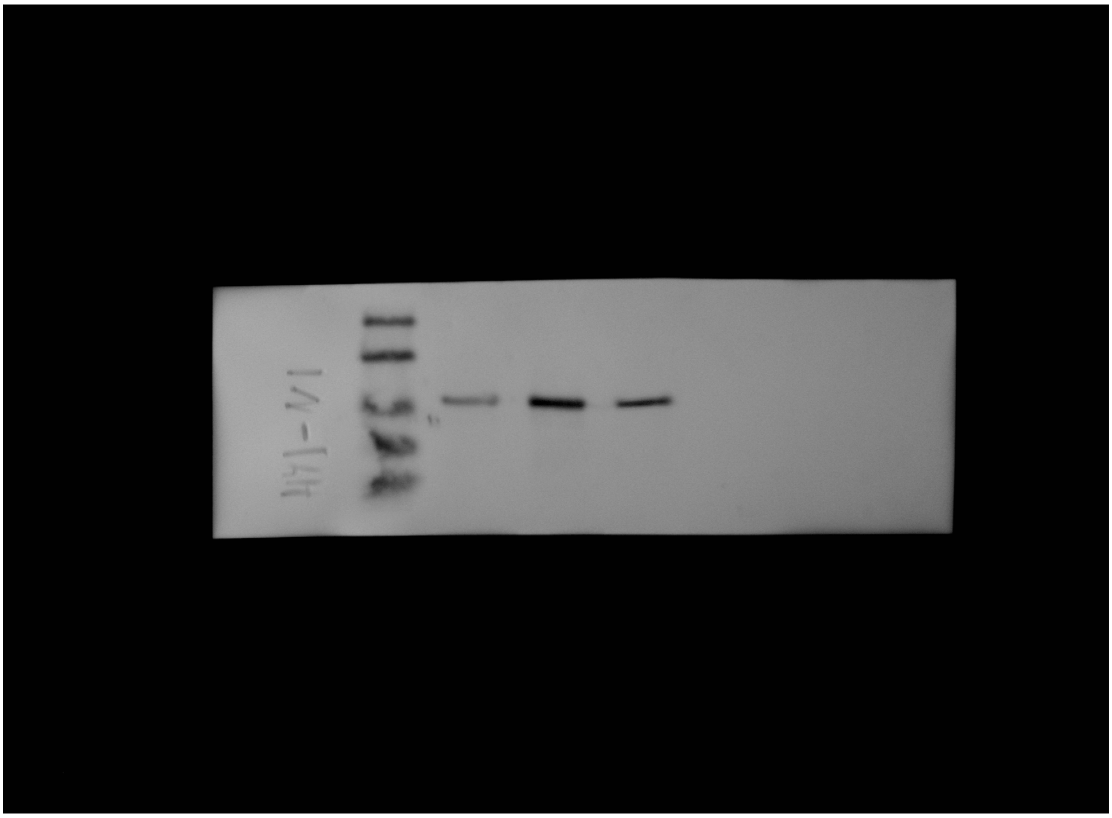

β-actin

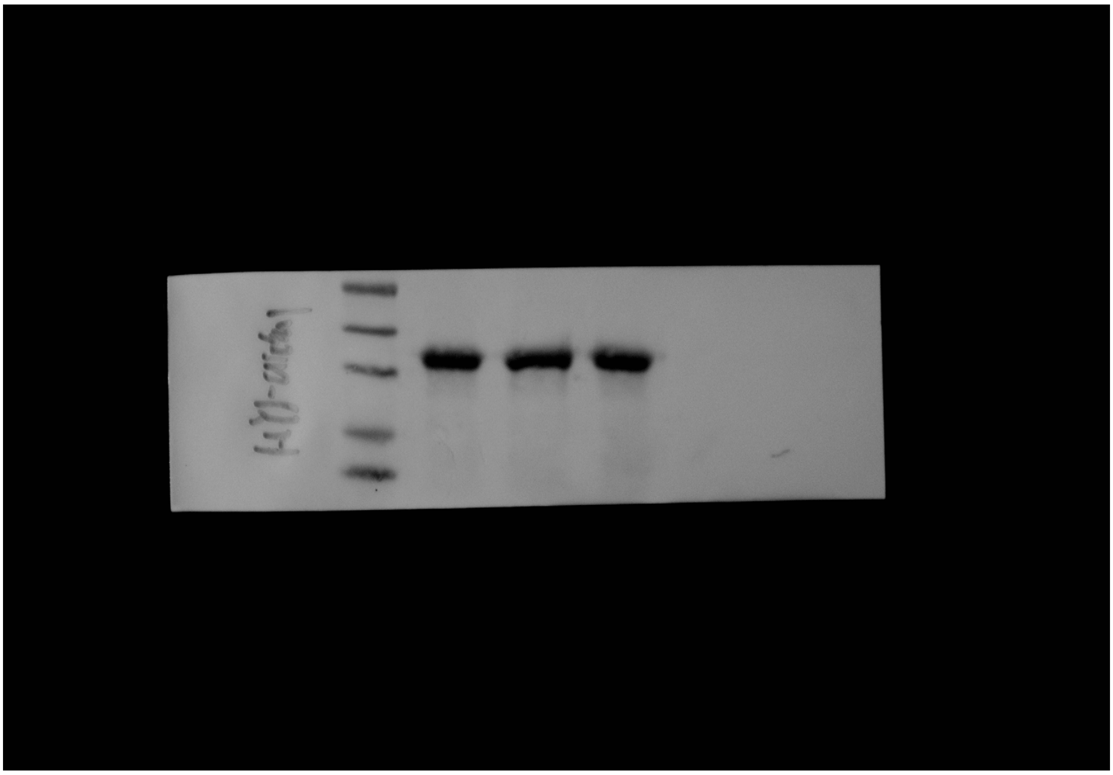

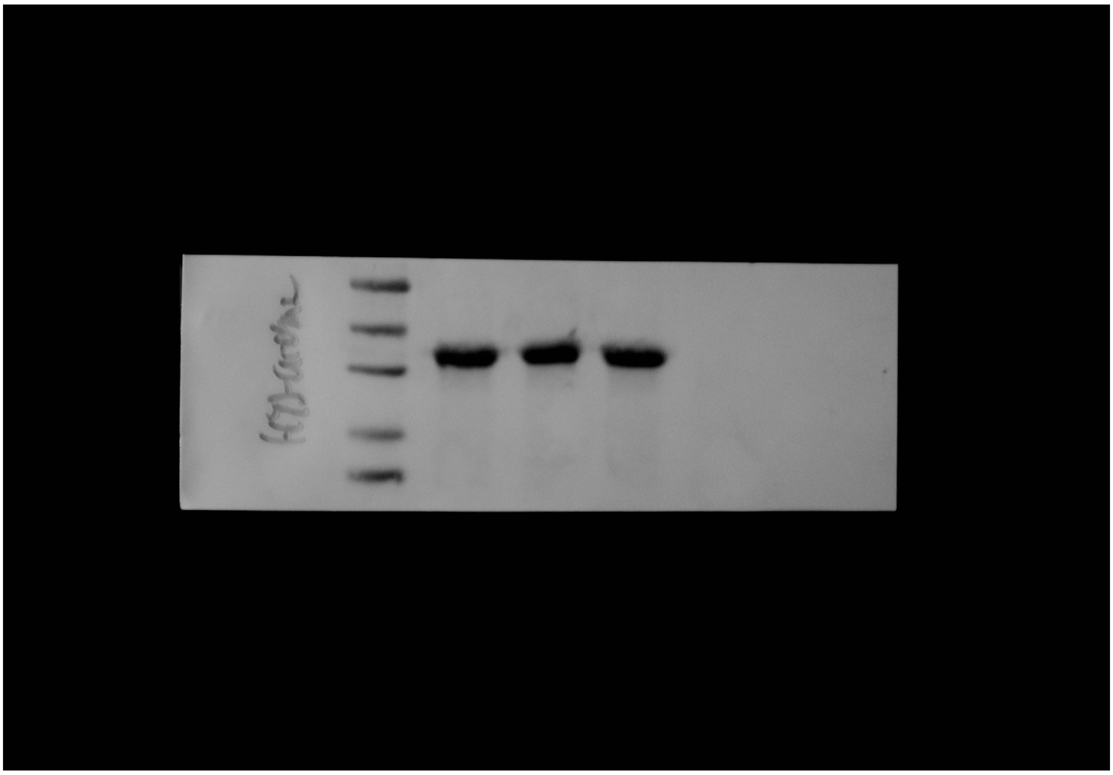

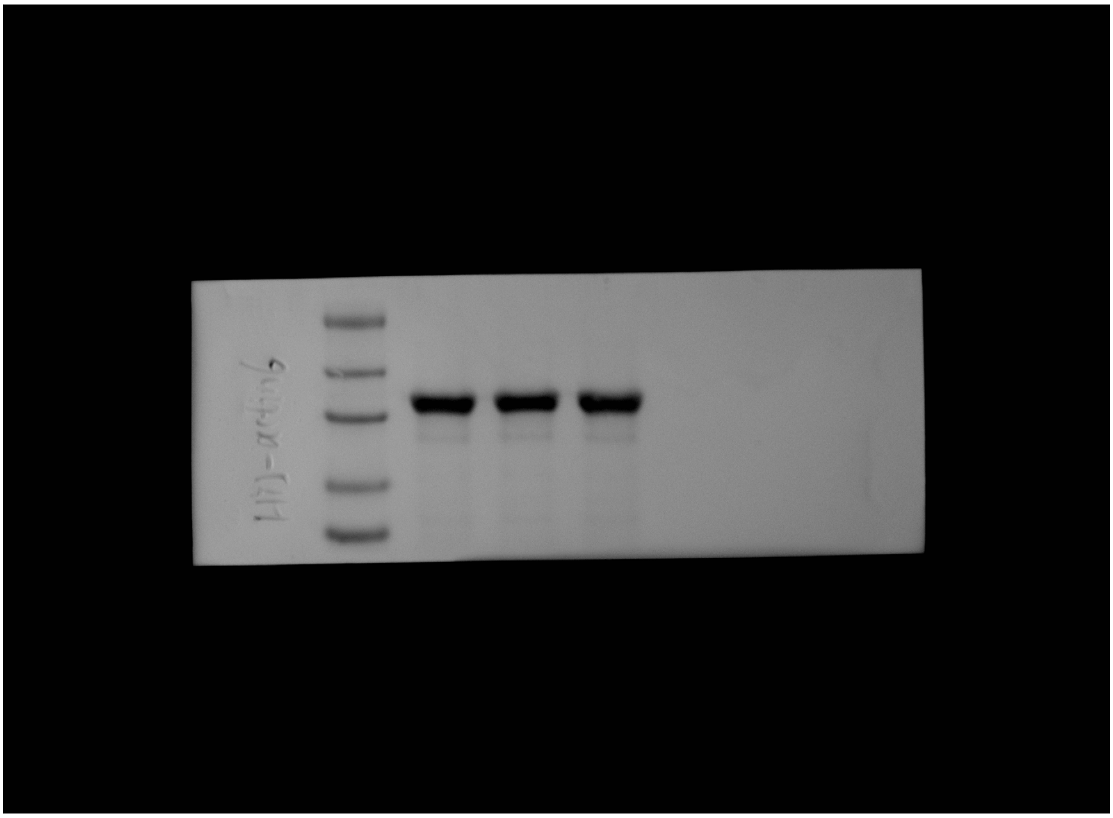

Figure 5B

BMAL1

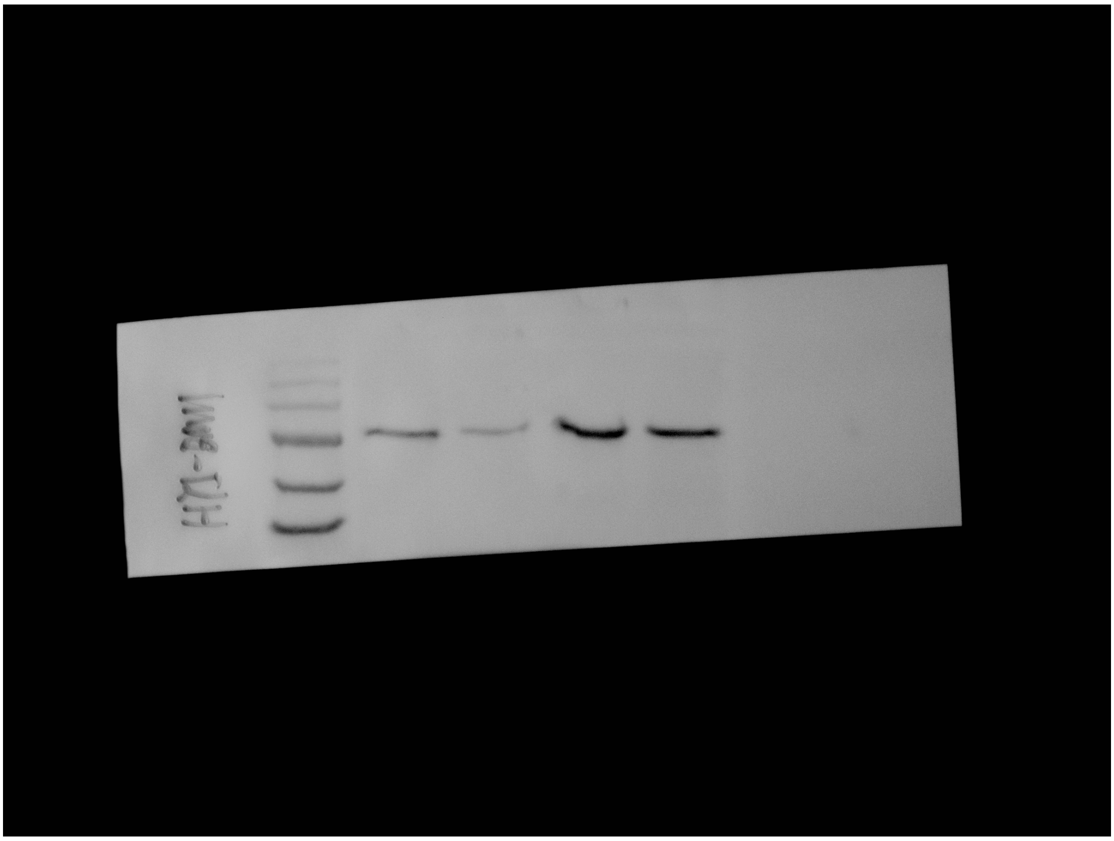

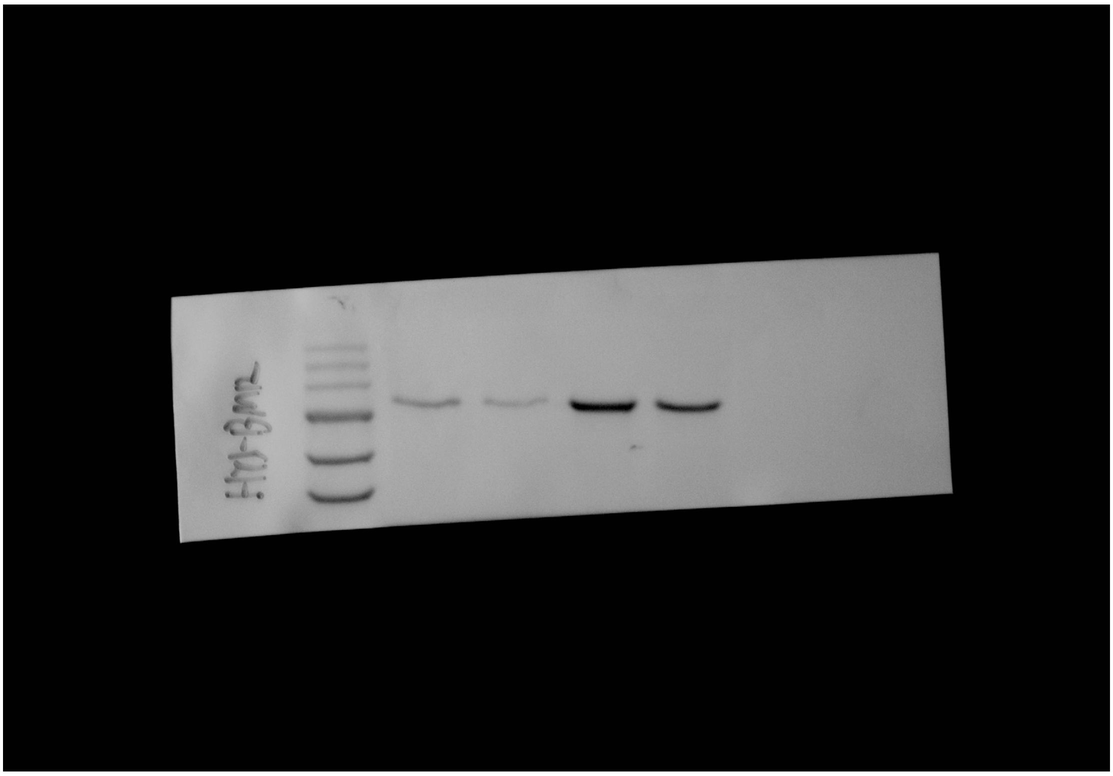

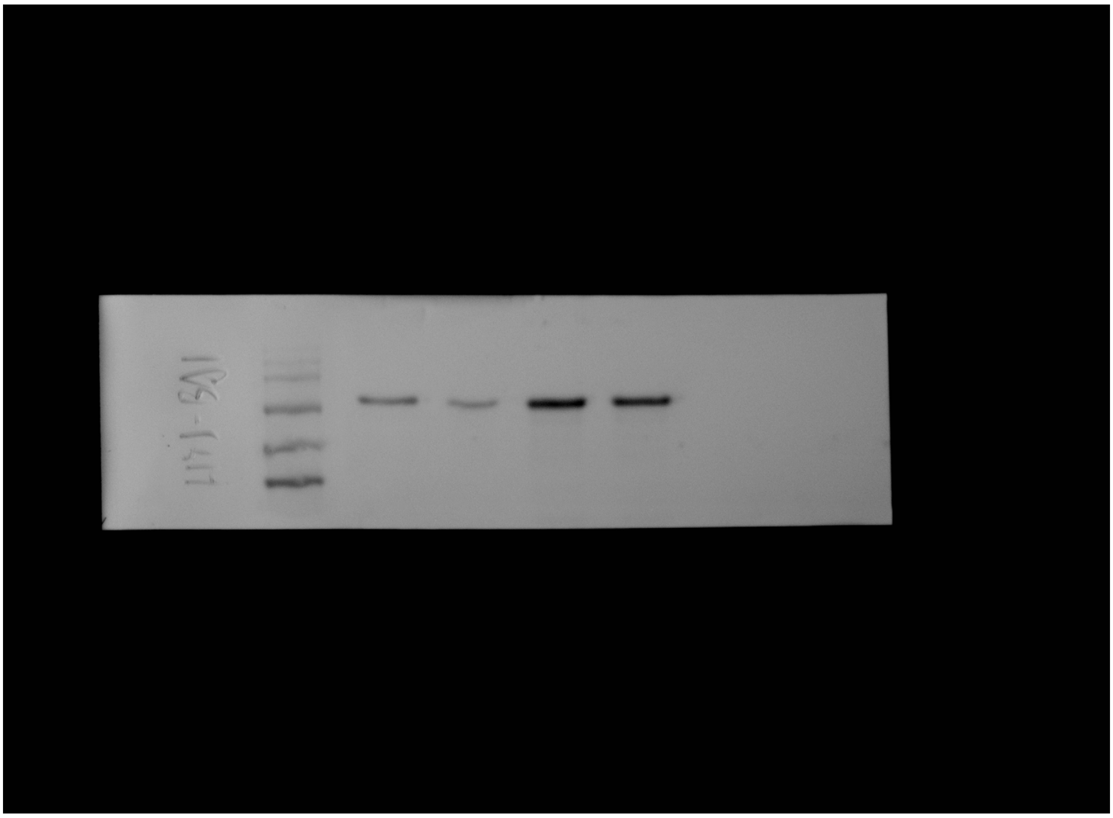

E2F1

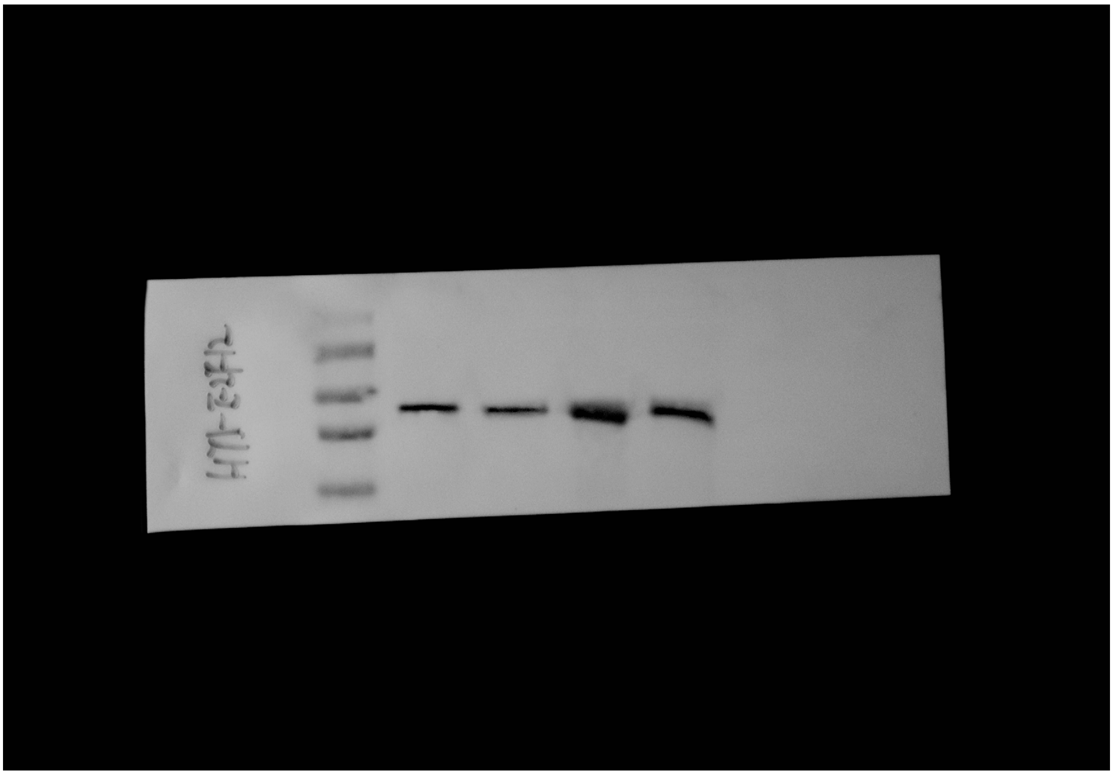

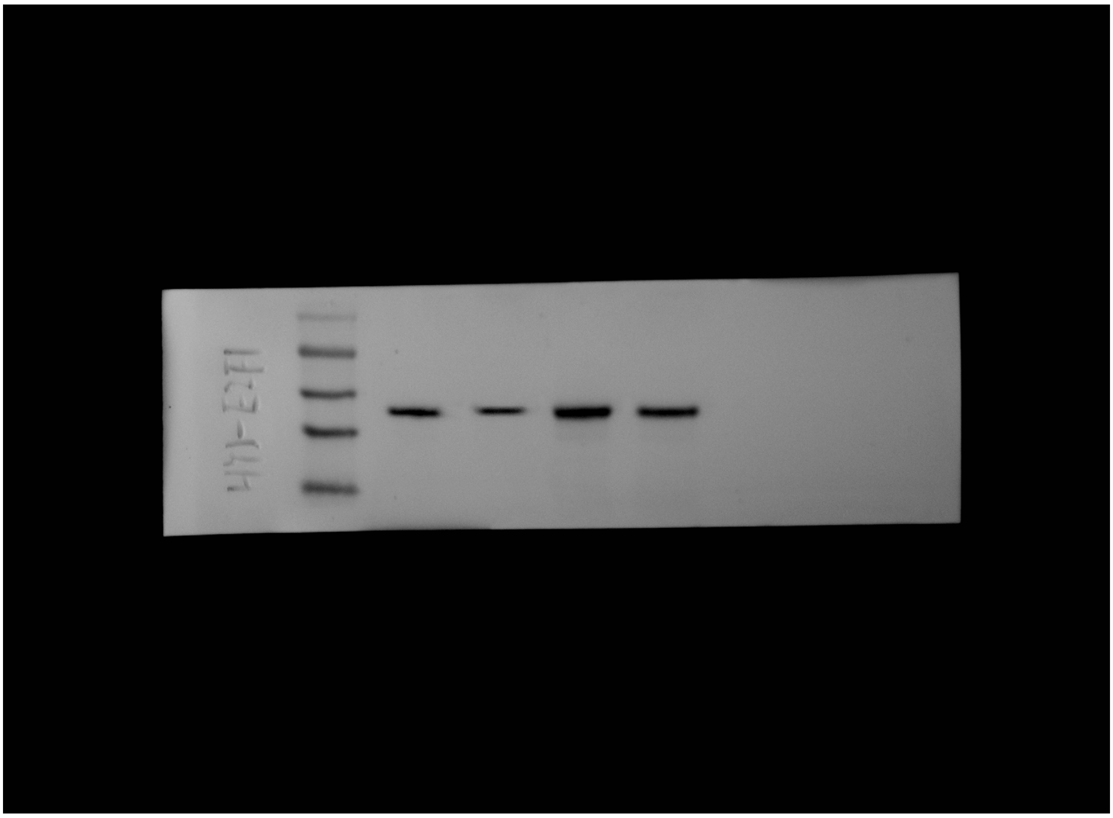

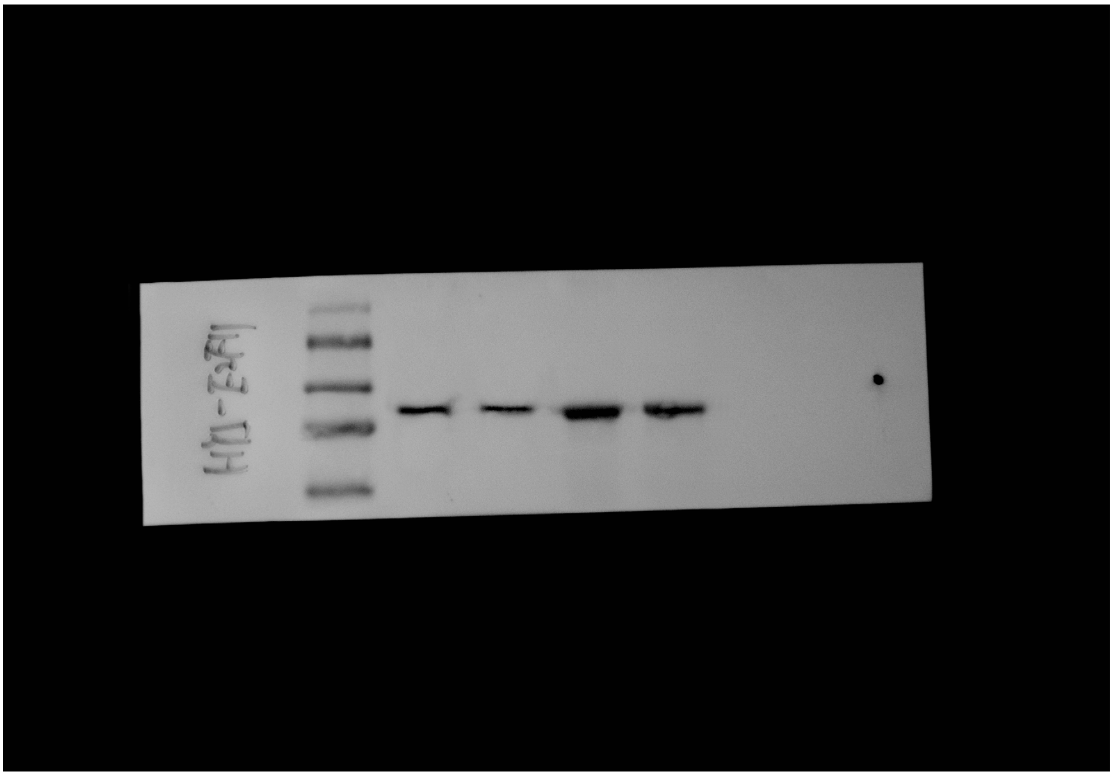

SIRT1

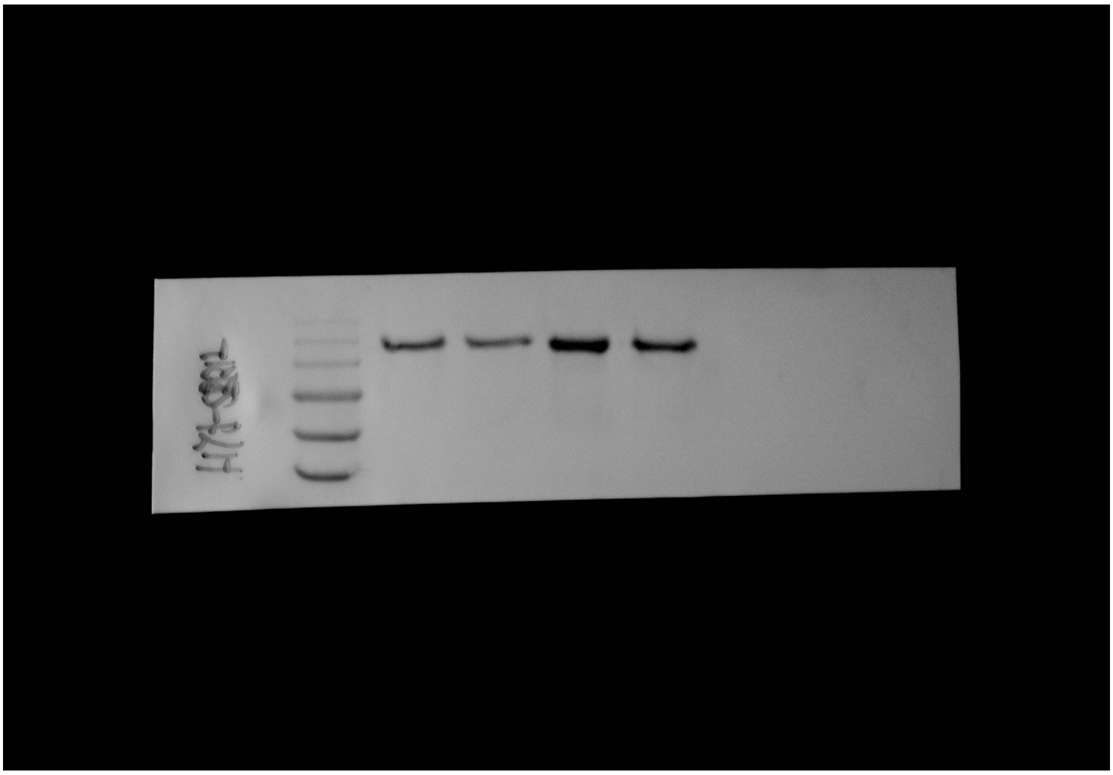

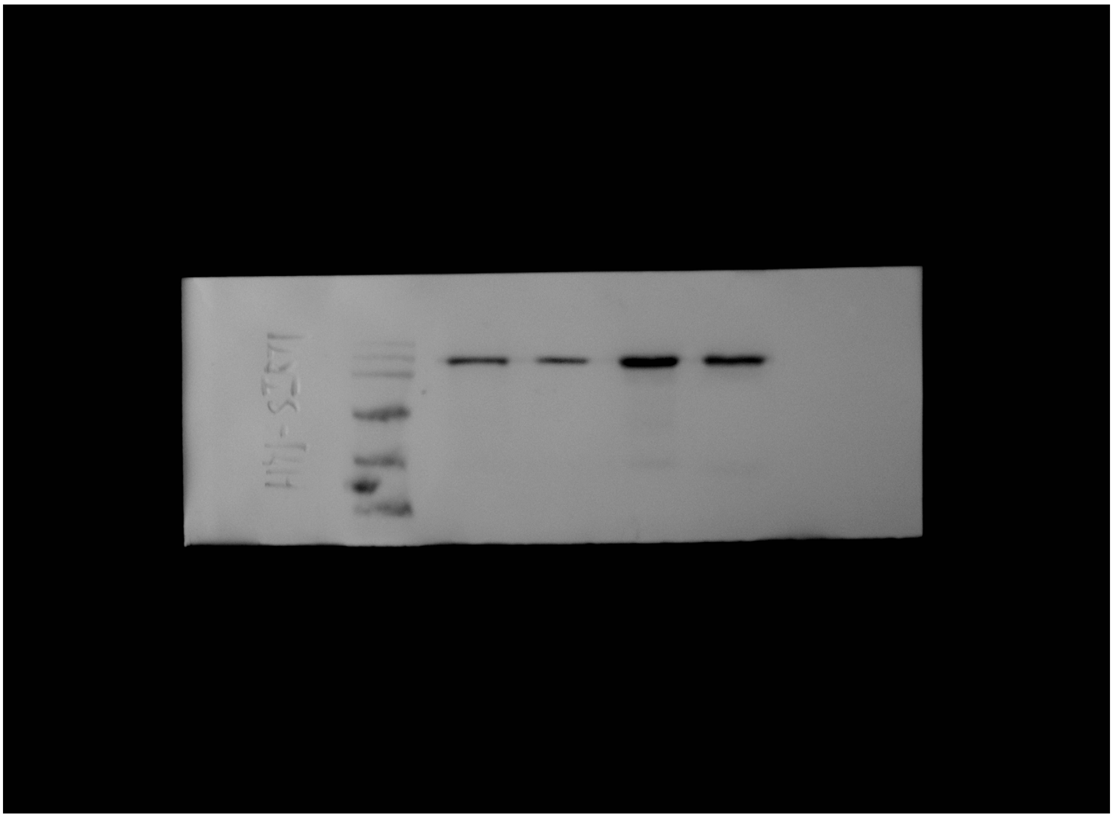

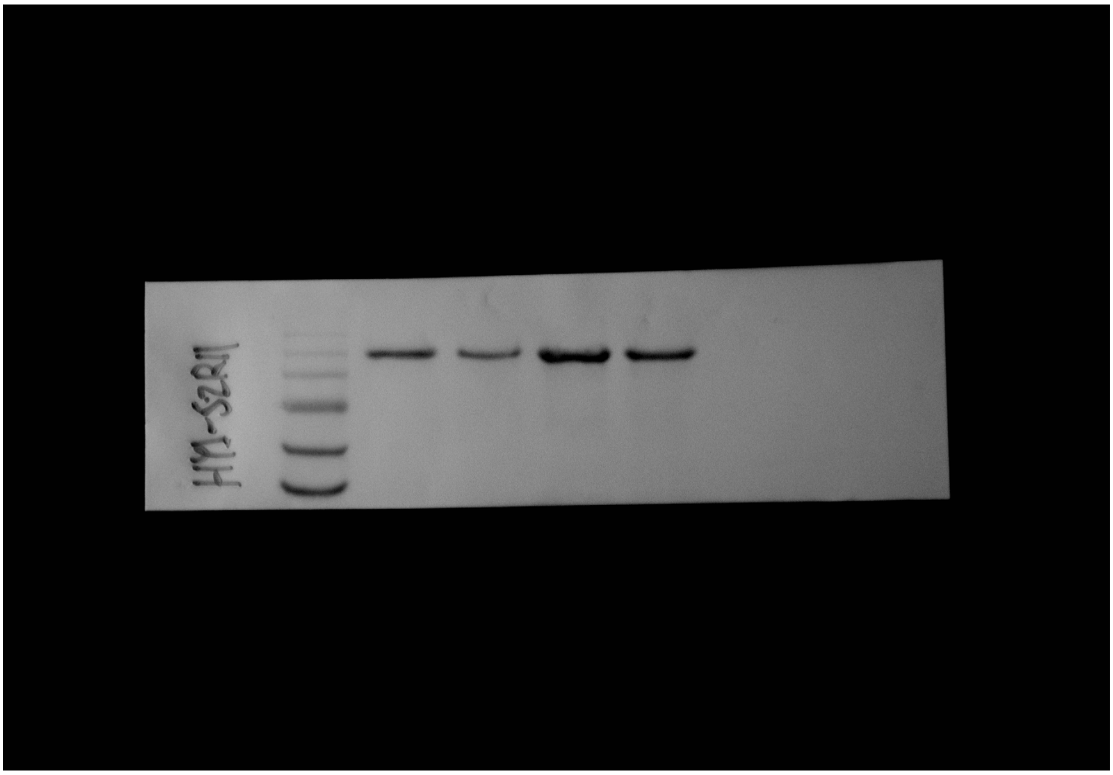

β-actin

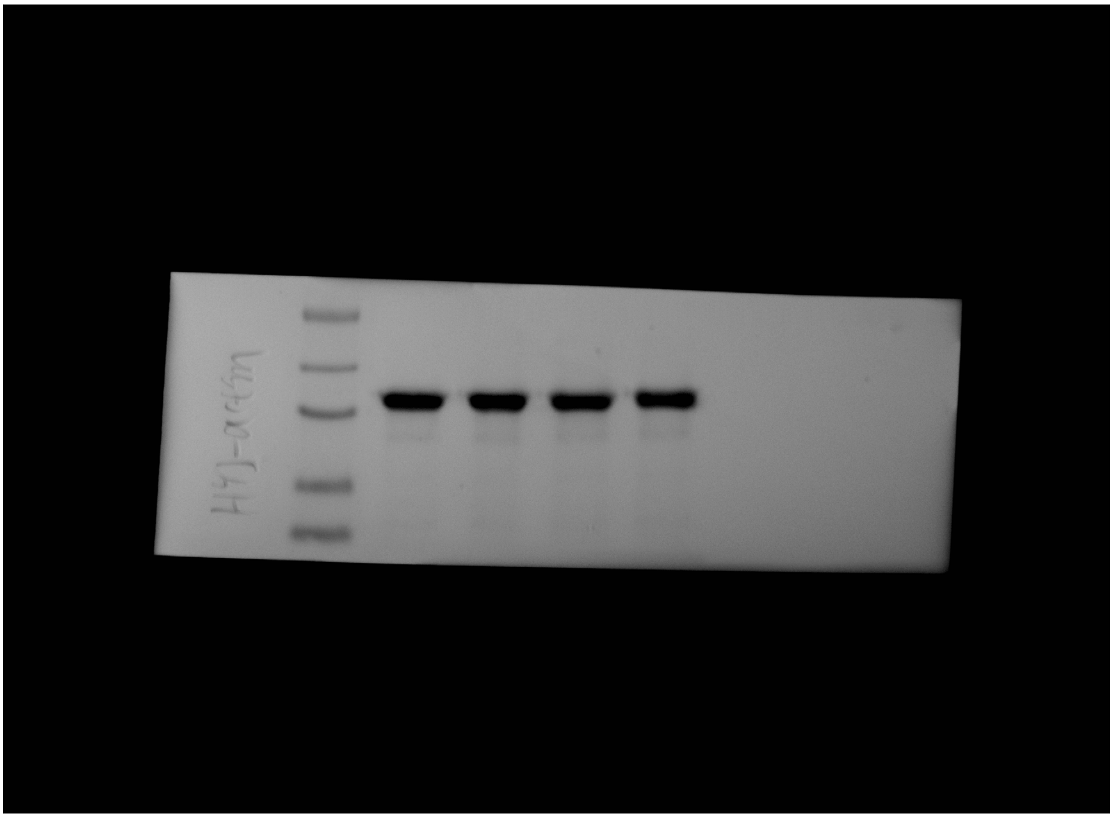

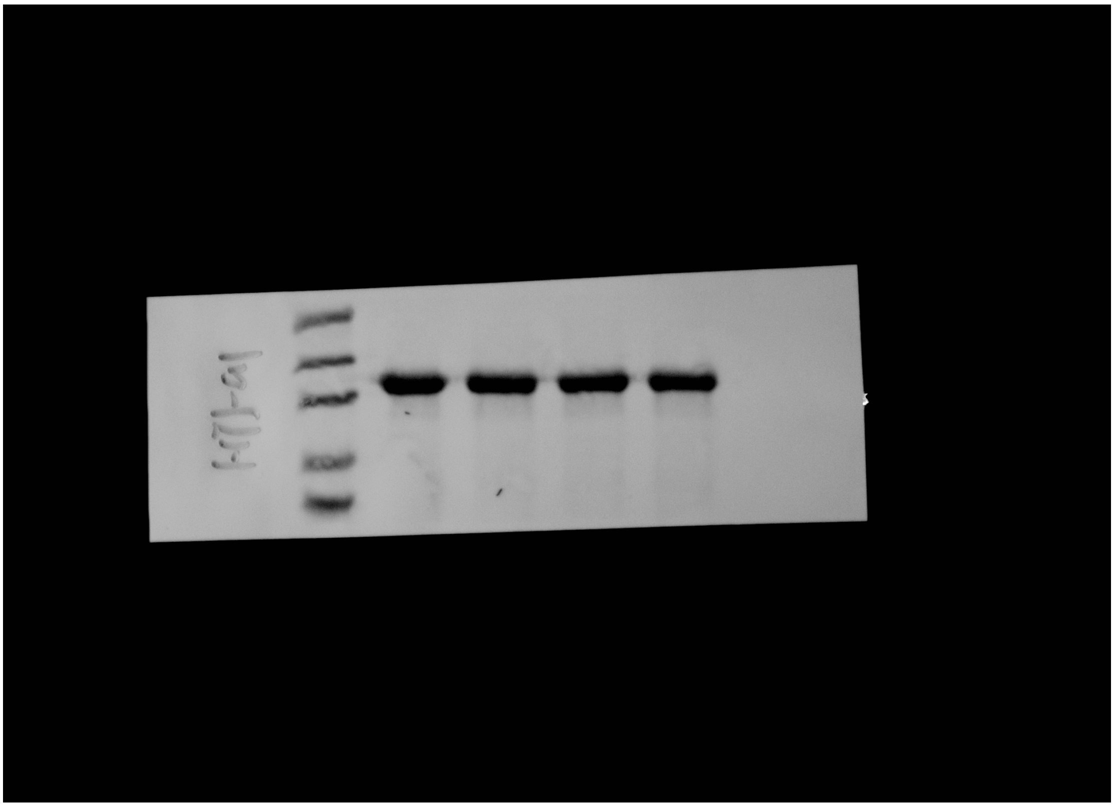

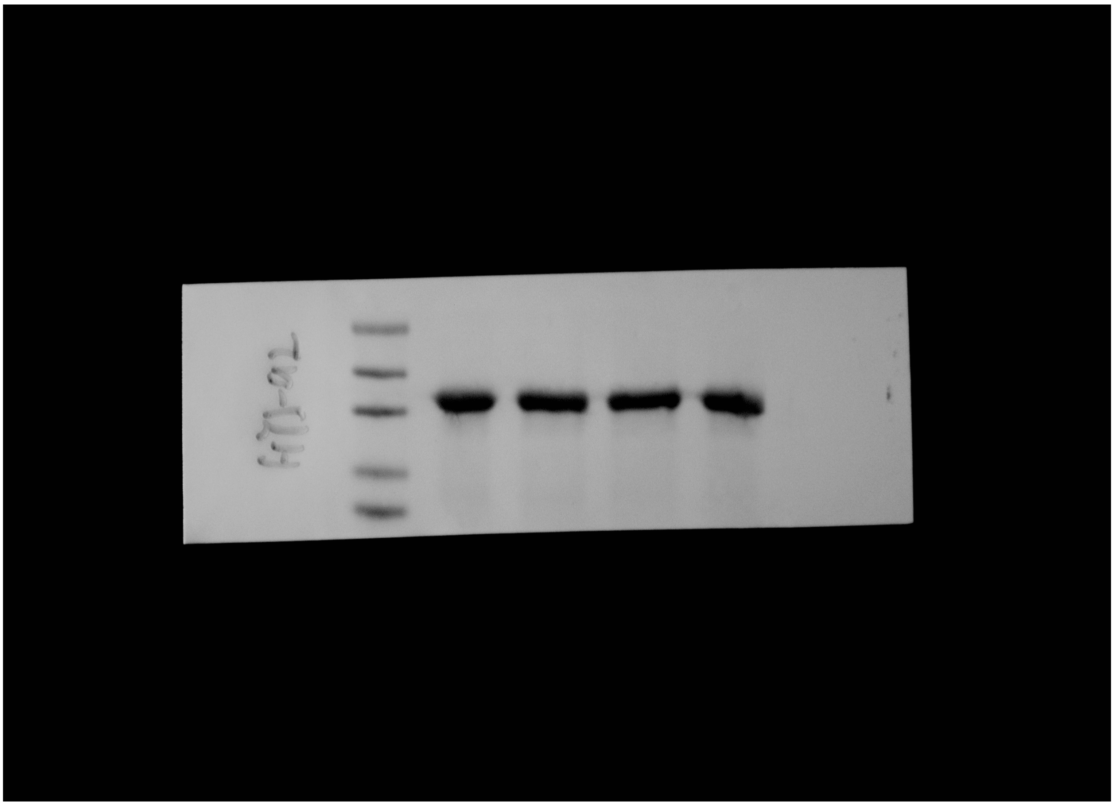

Figure 5E

GCLC

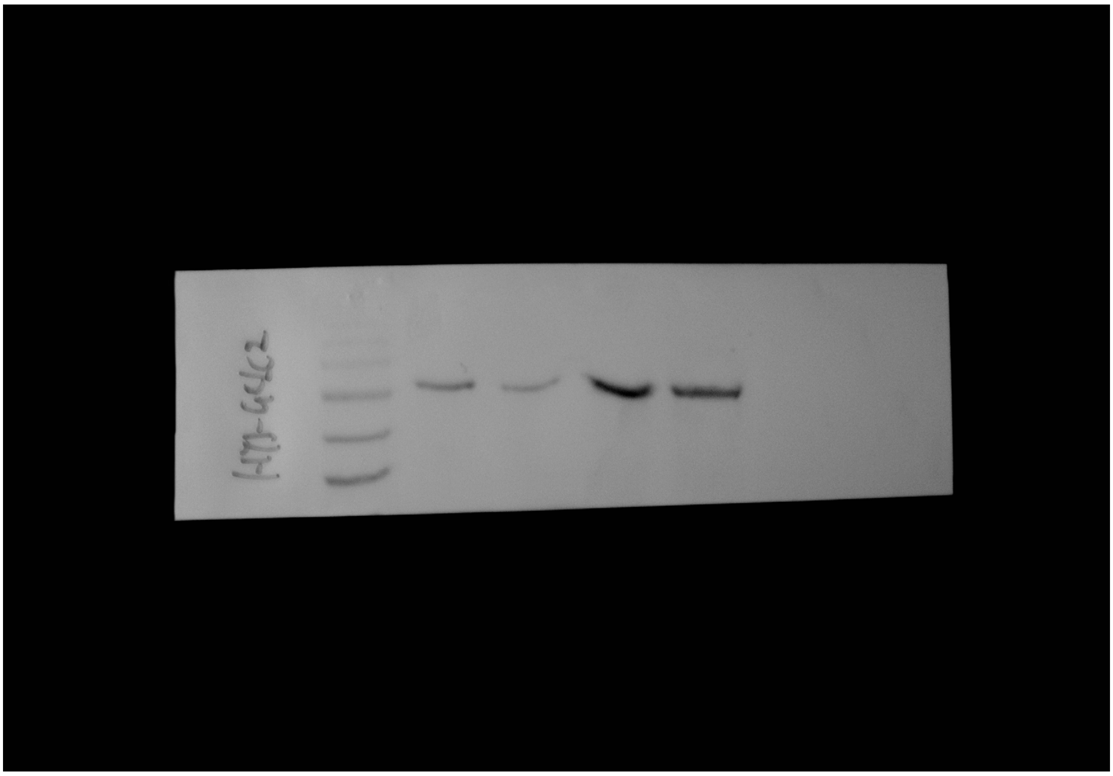

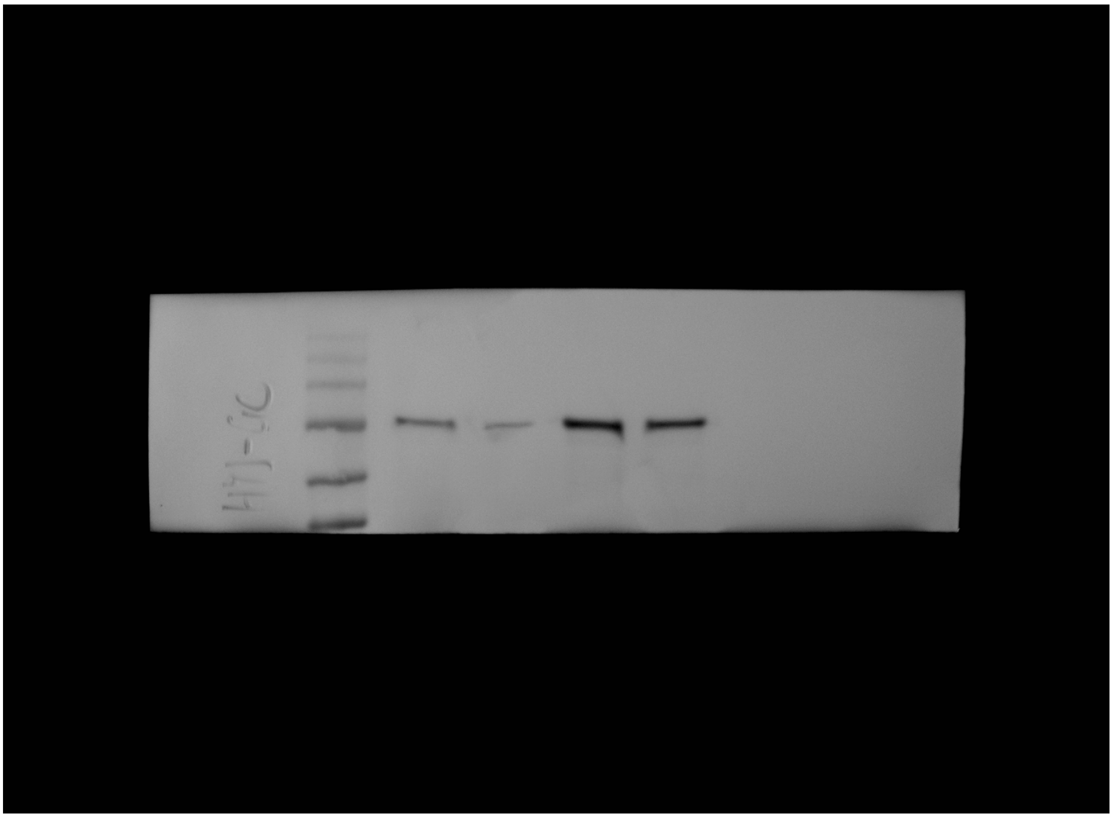

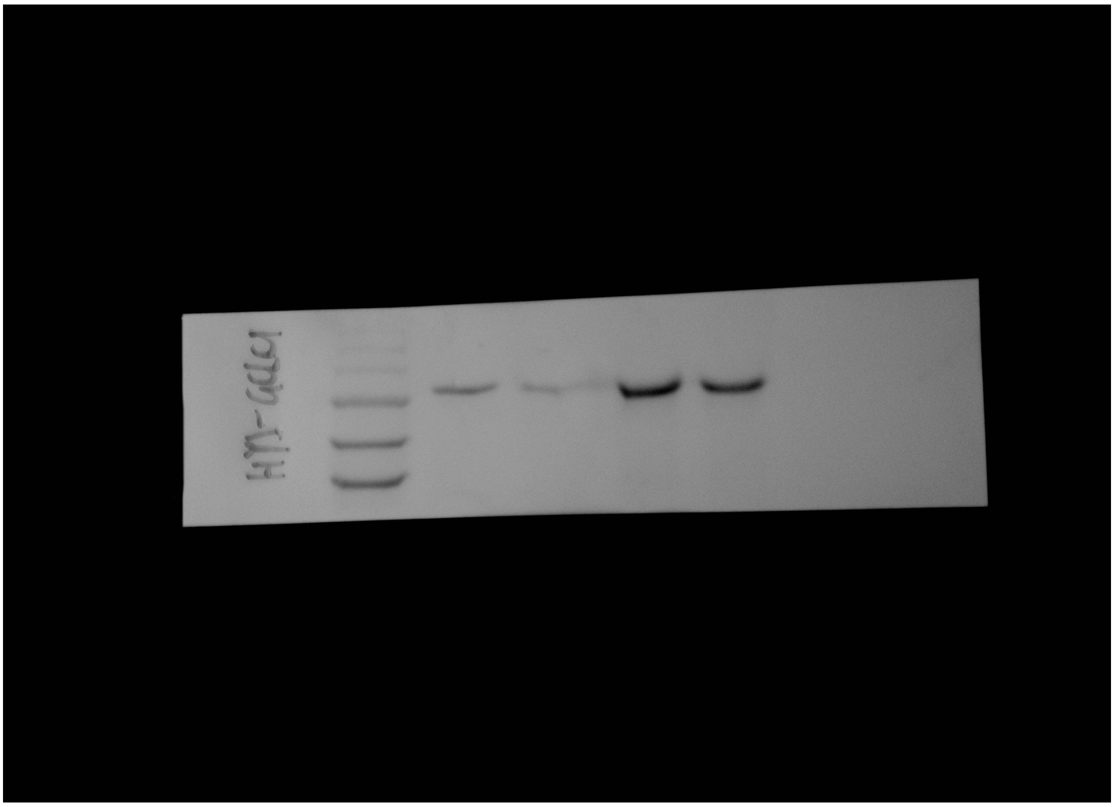

GCLM

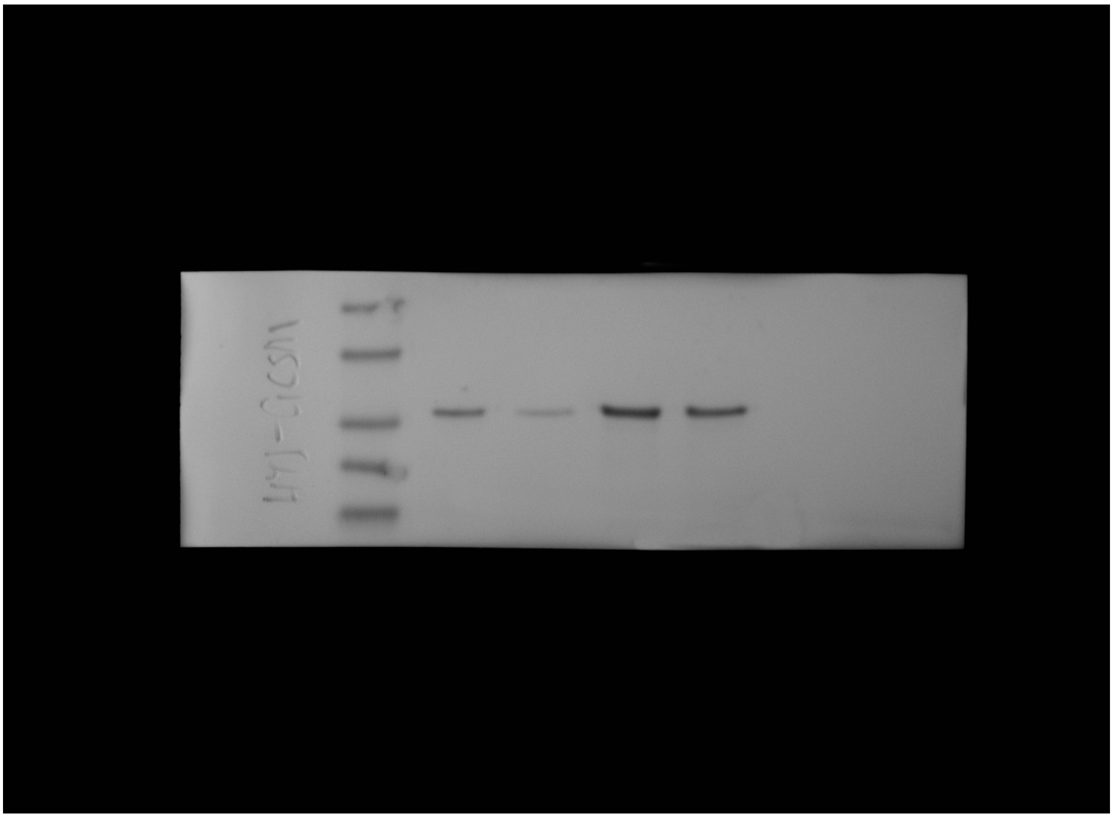

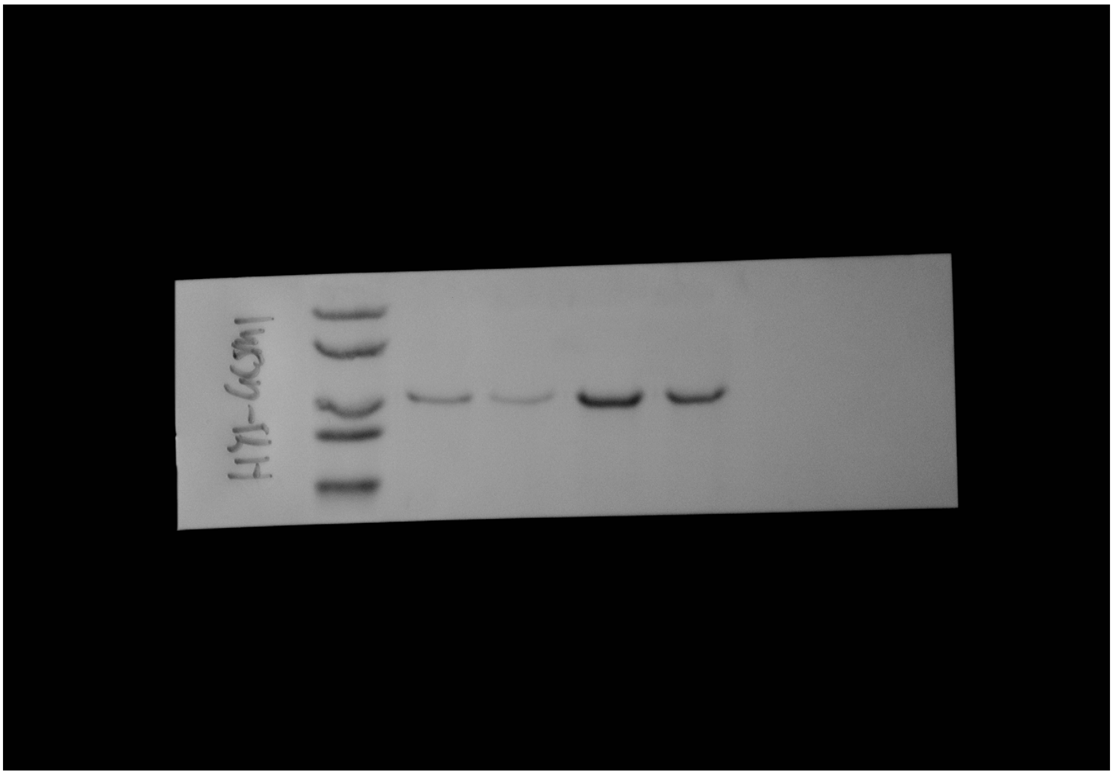

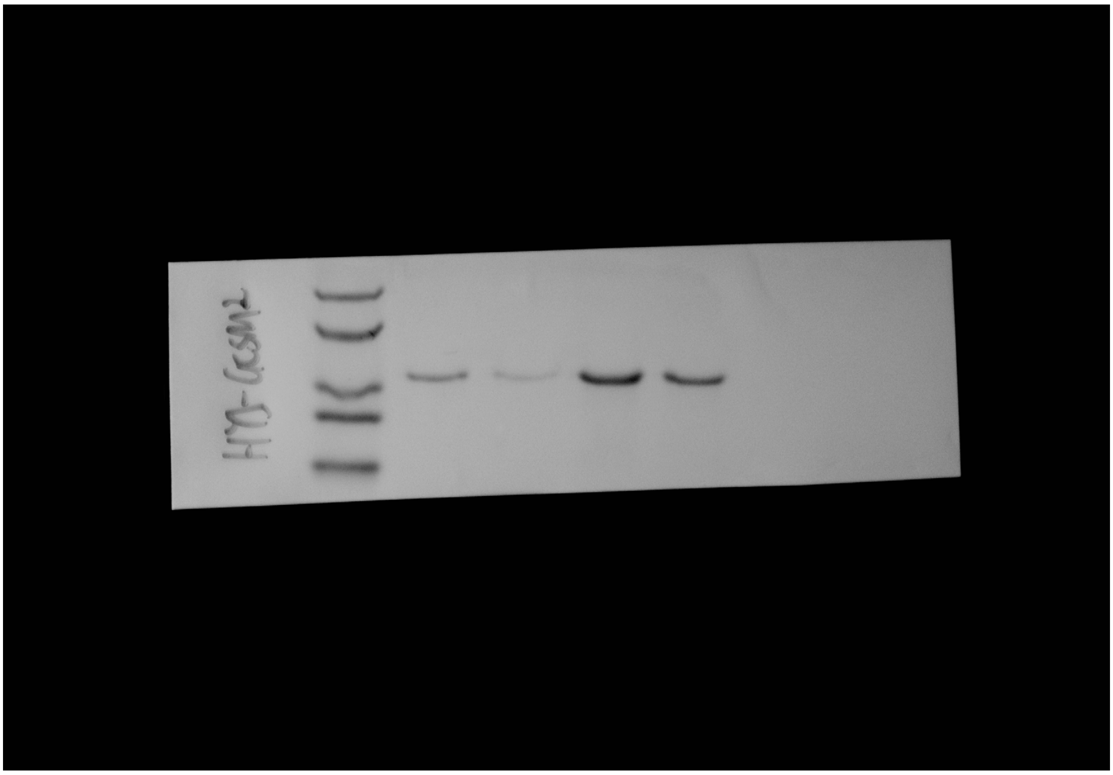

NQO1

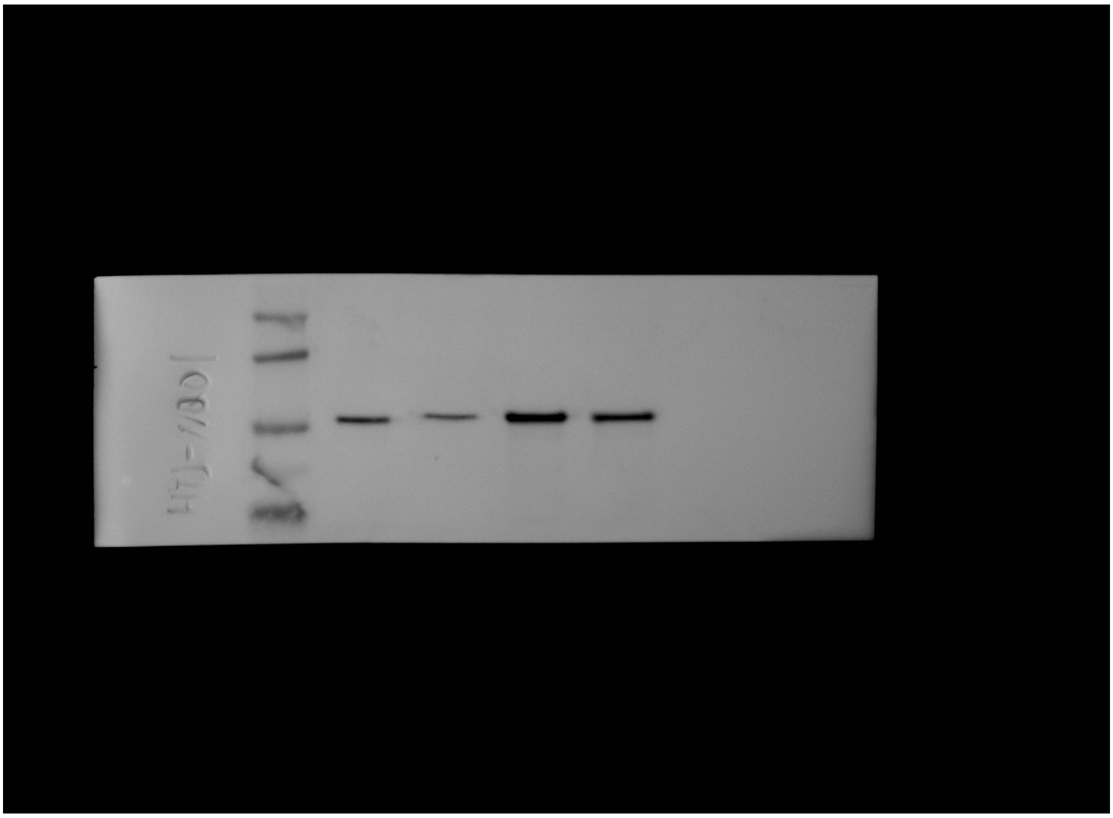

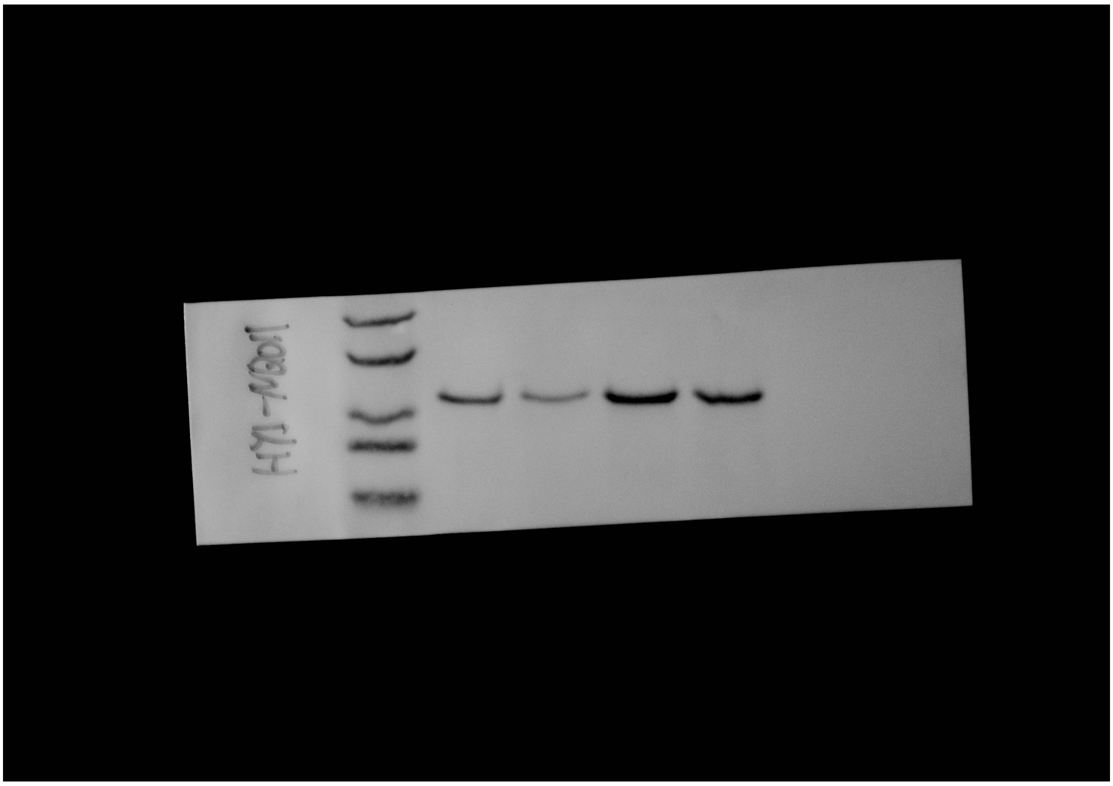

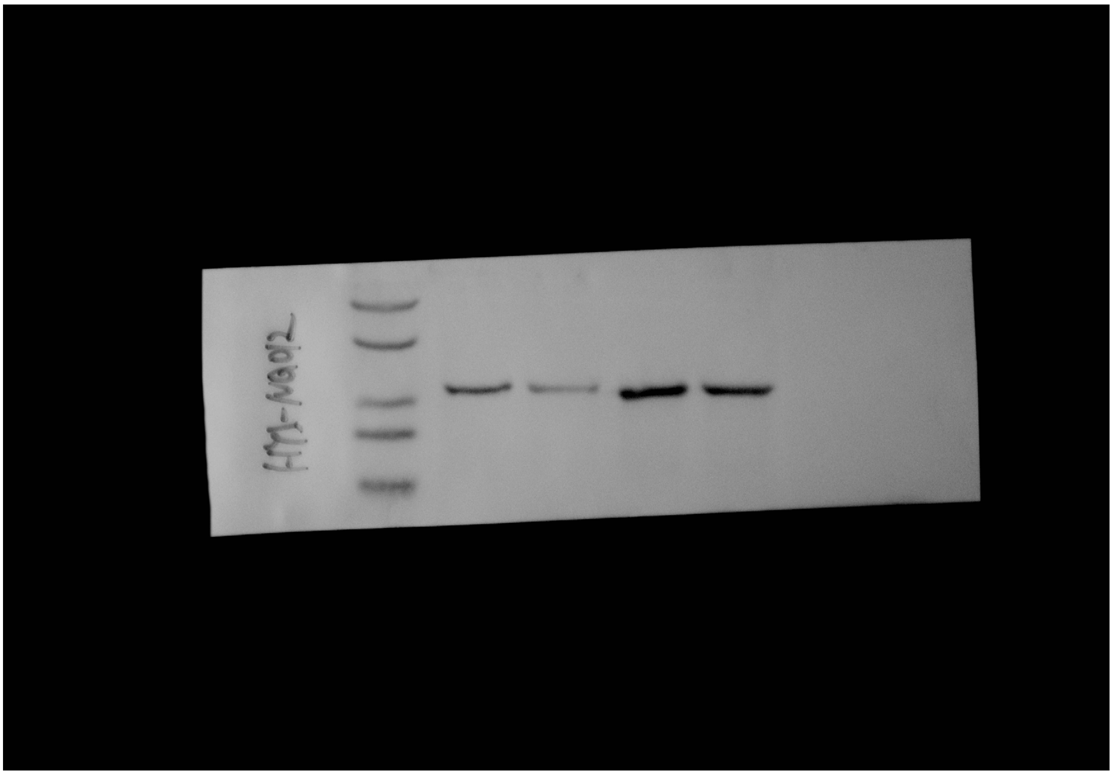

β-actin

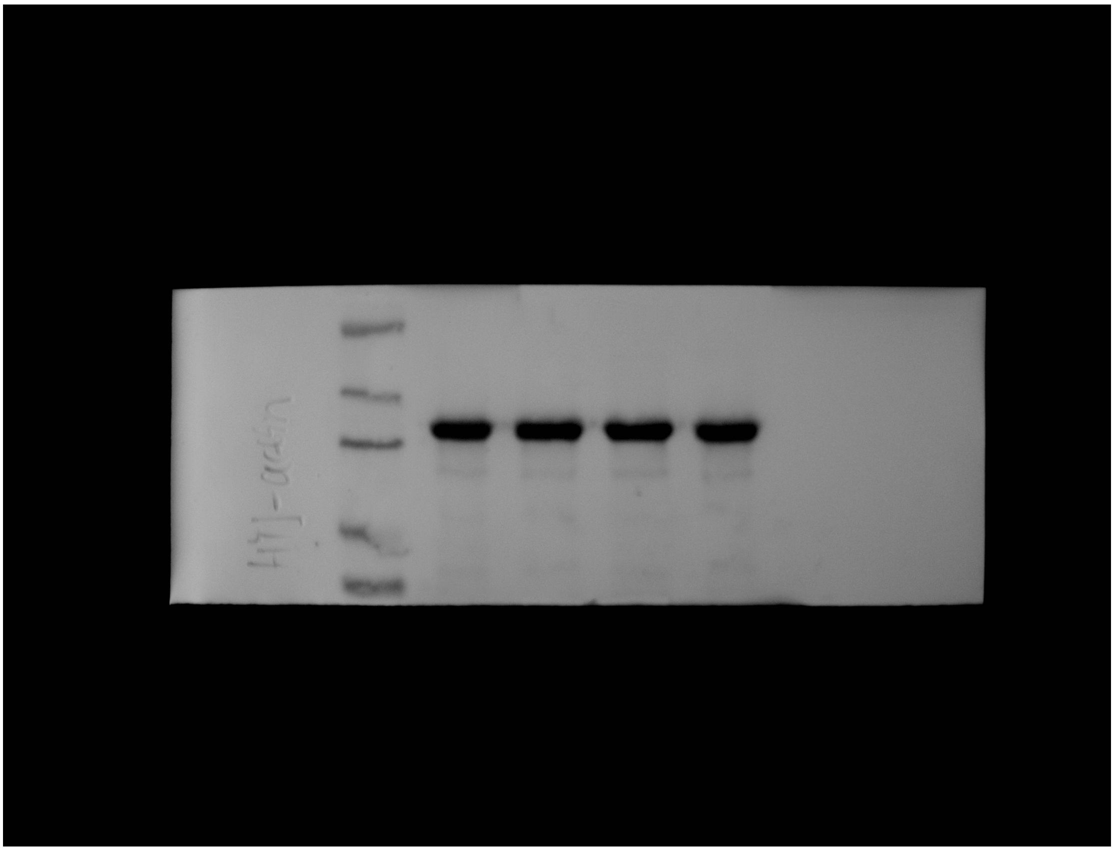

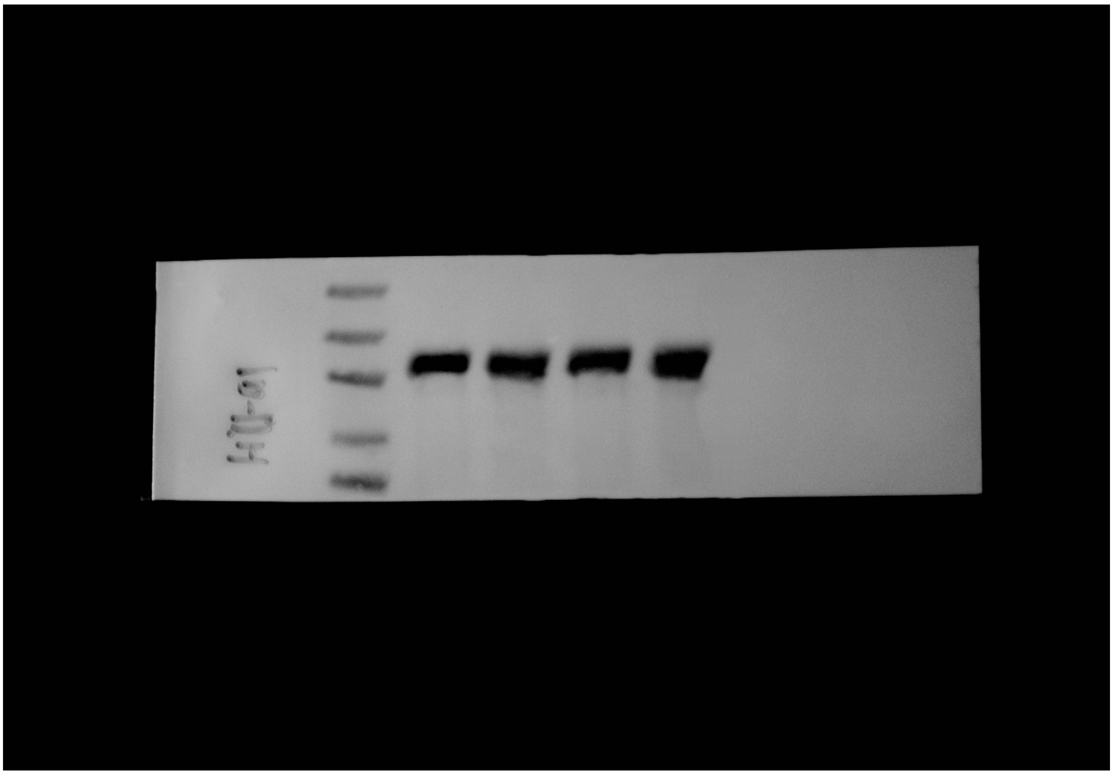

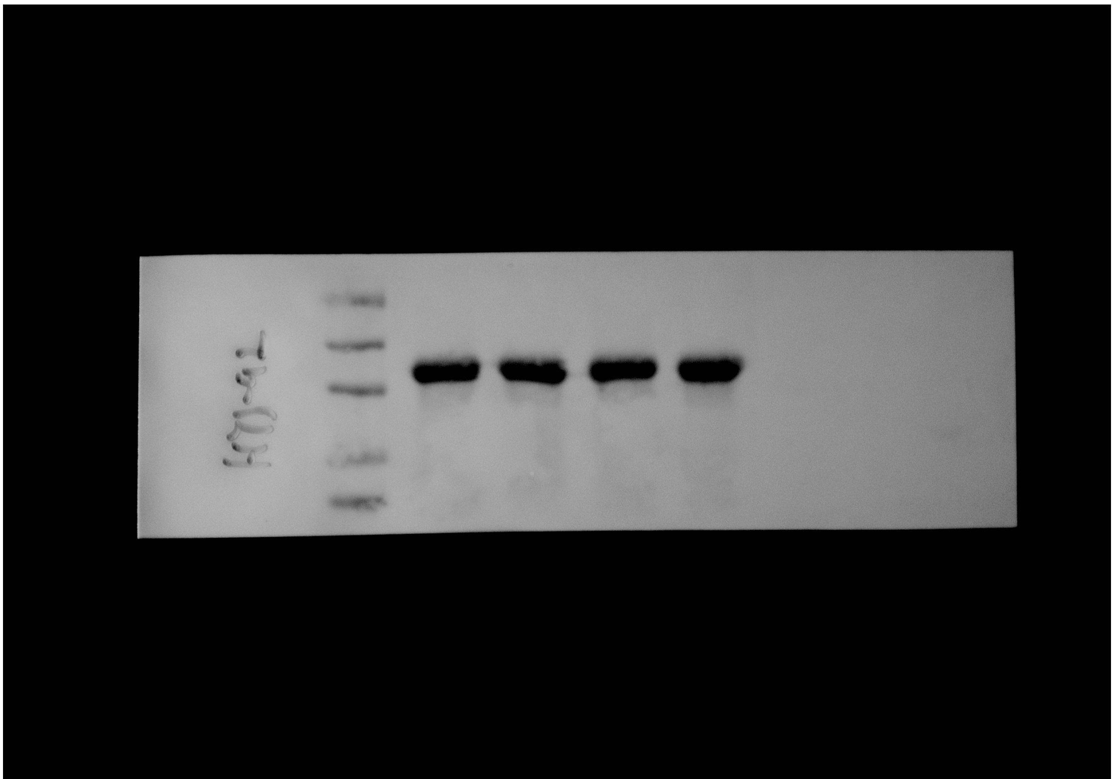

Figure 5F

BMAL1

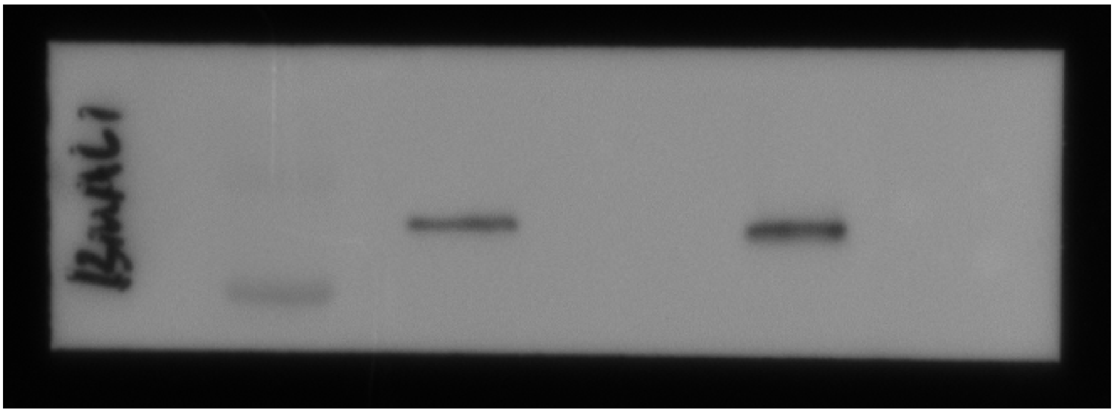

SIRT1

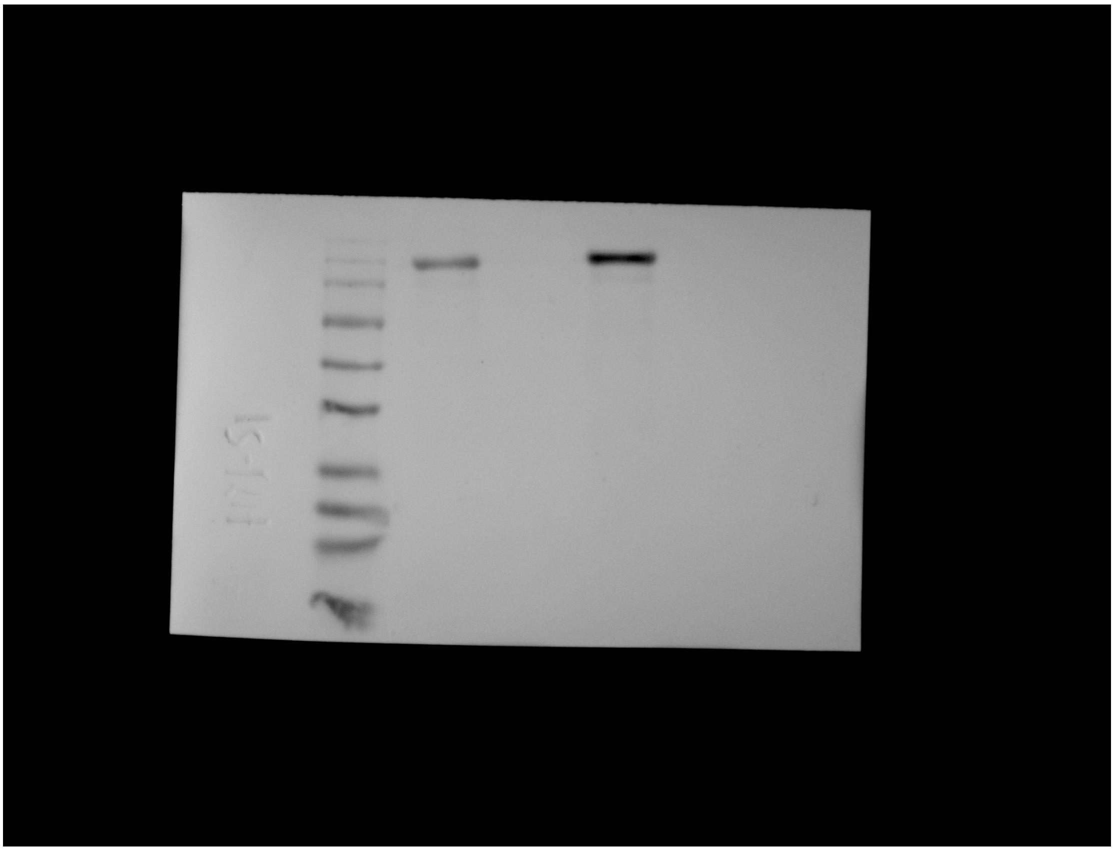

Figure 6B

BMAL1

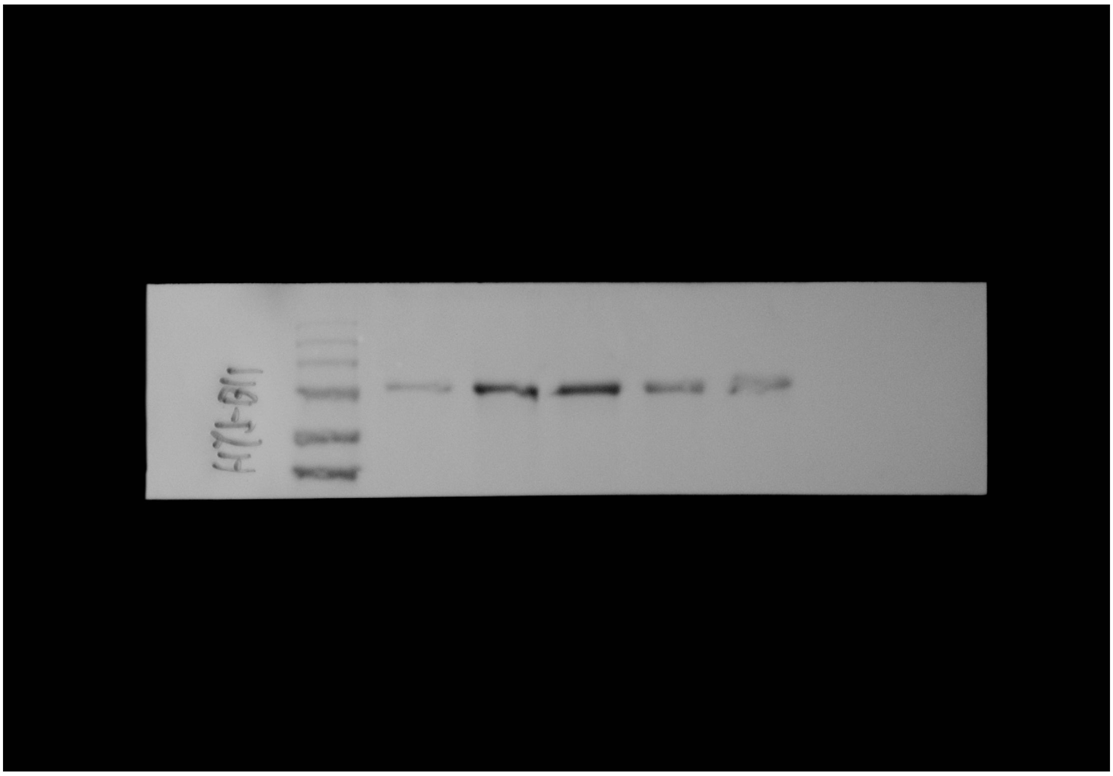

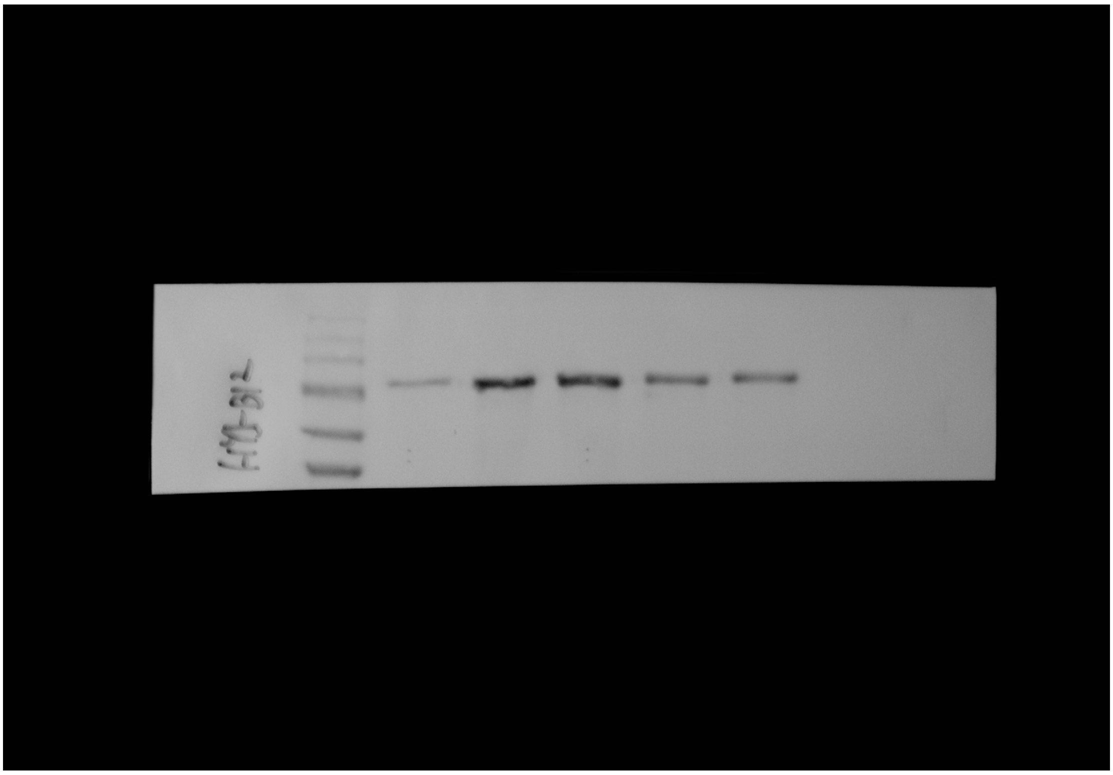

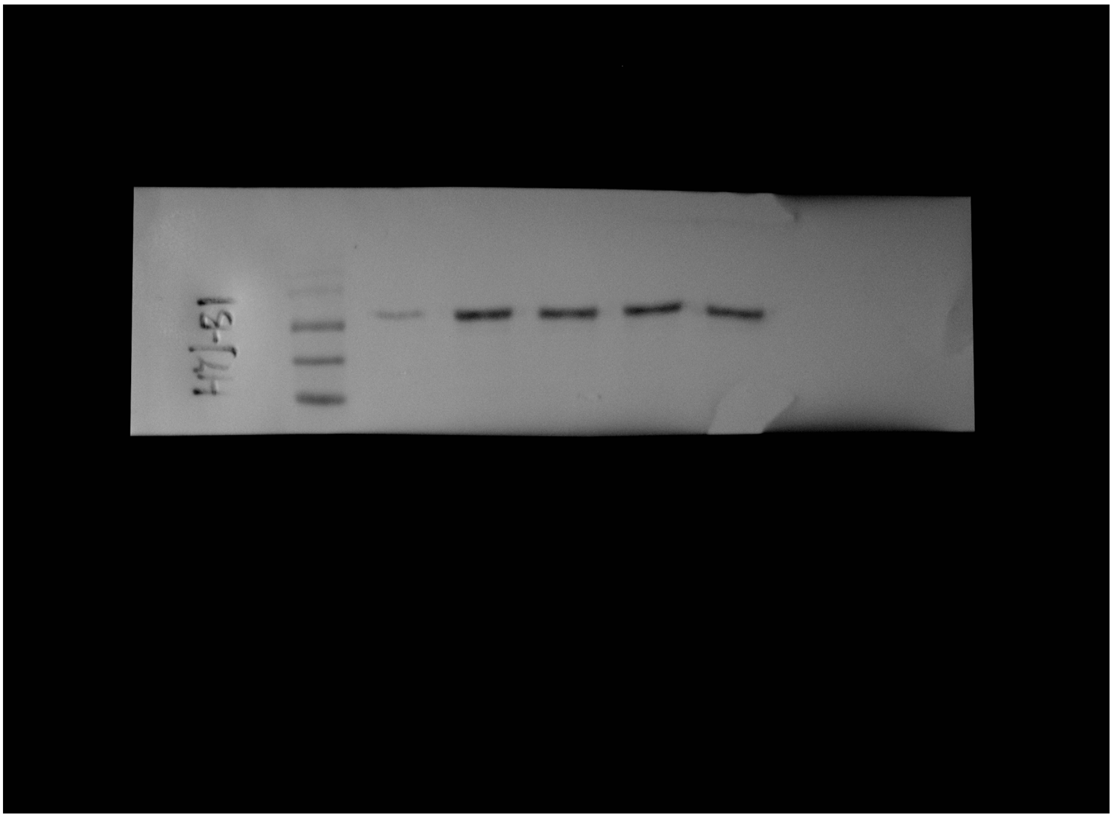

E2F1

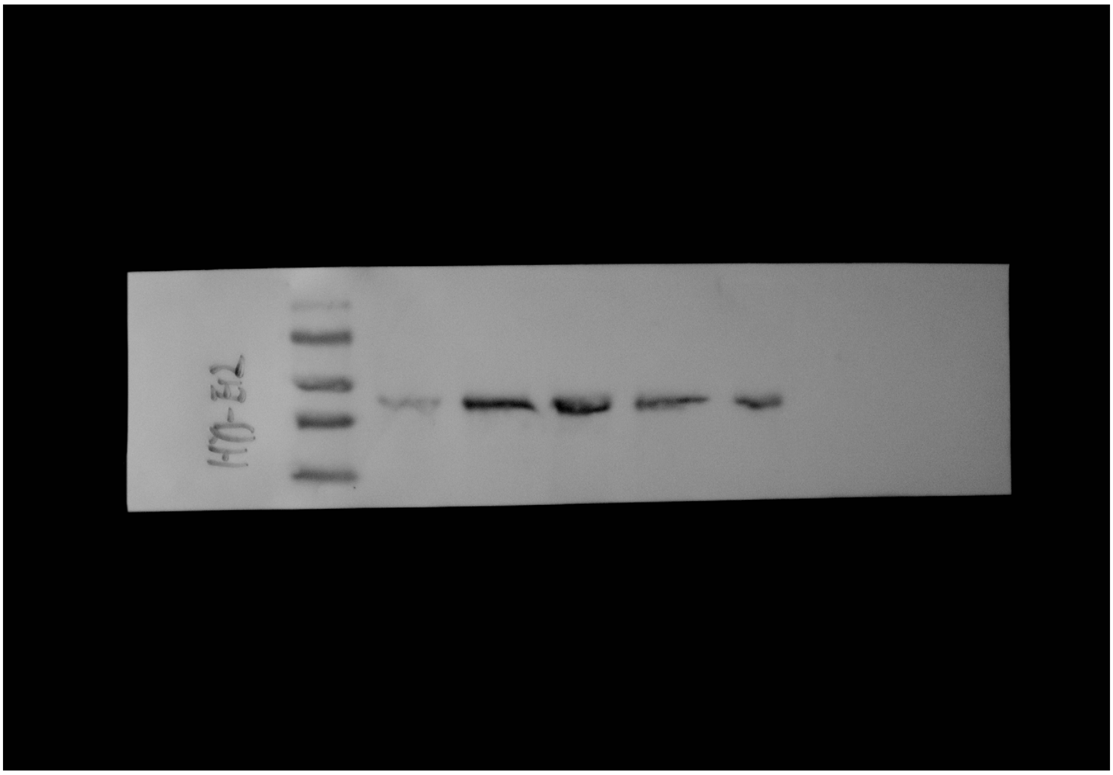

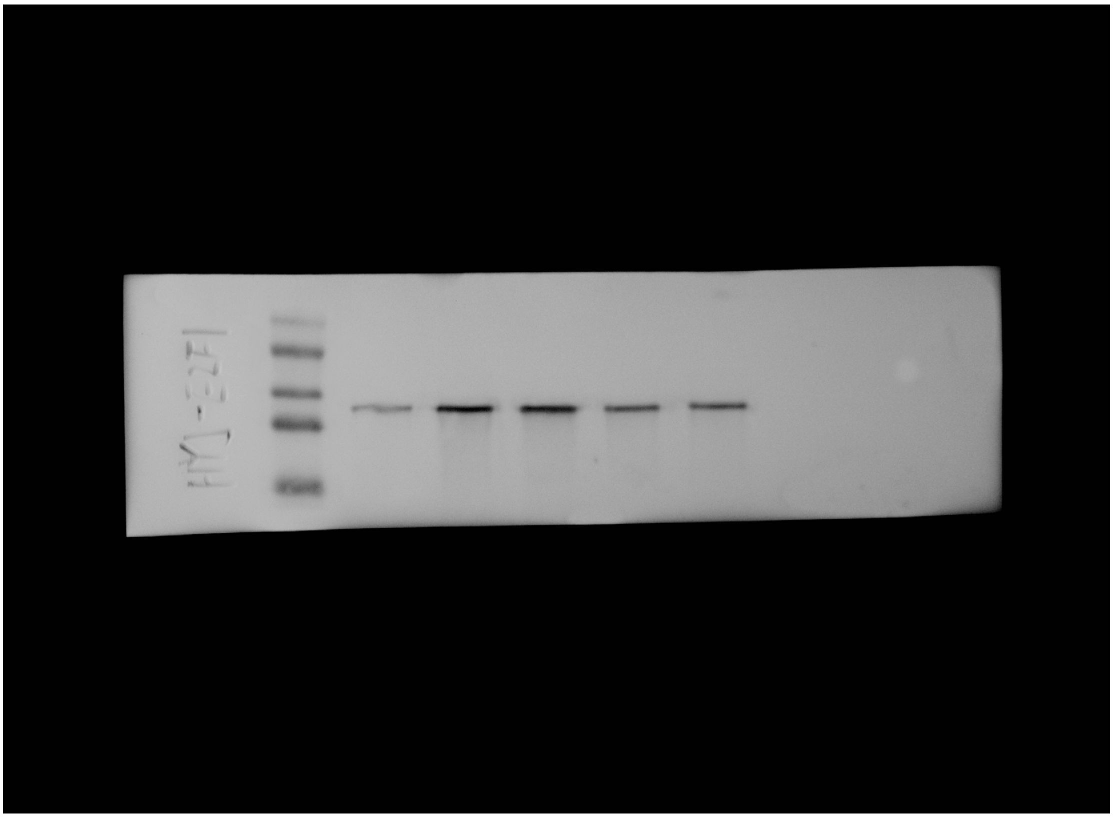

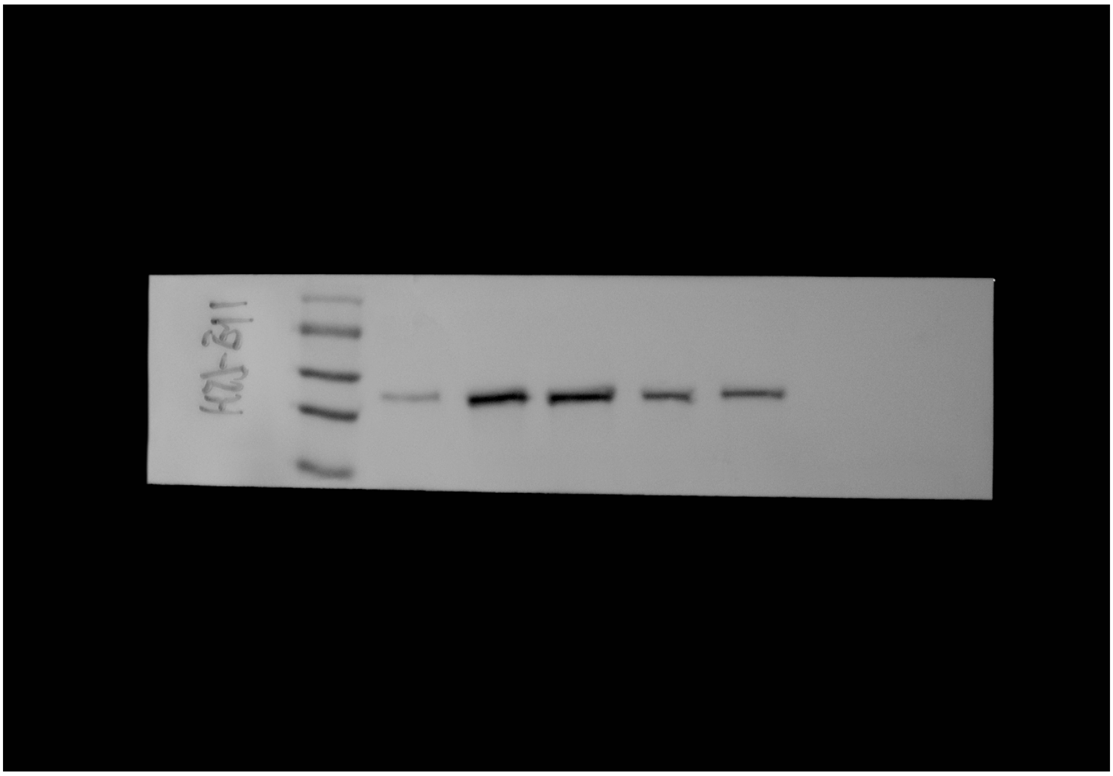

SIRT1

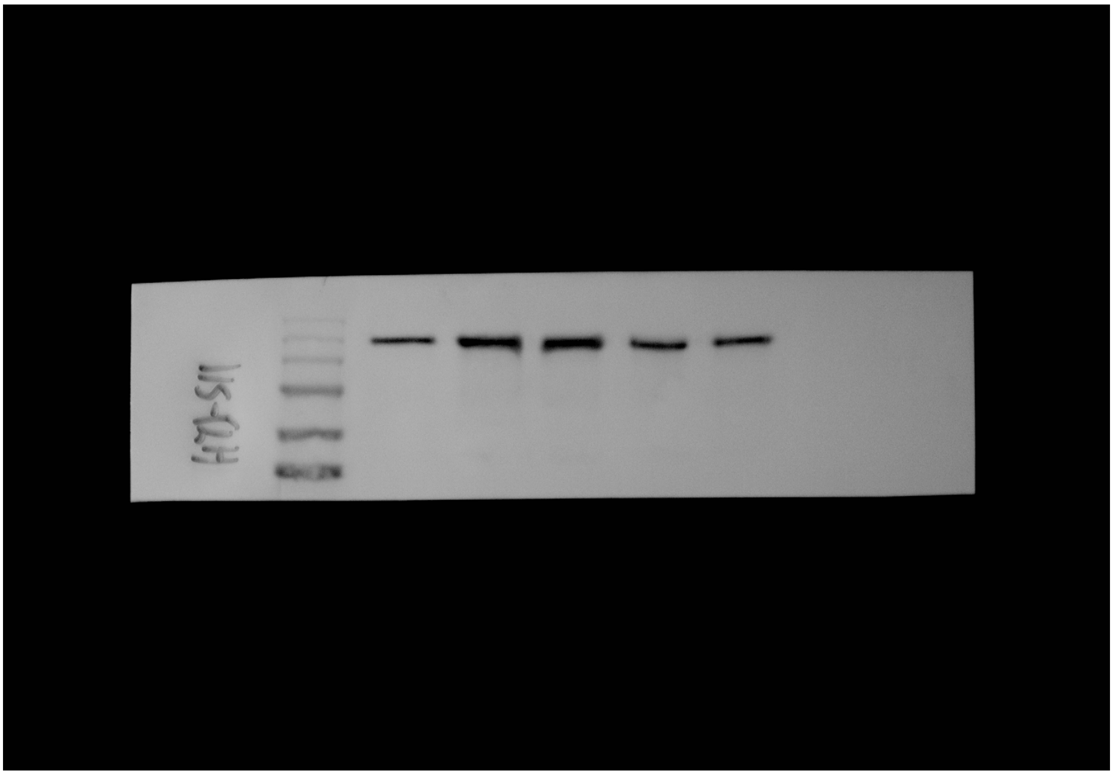

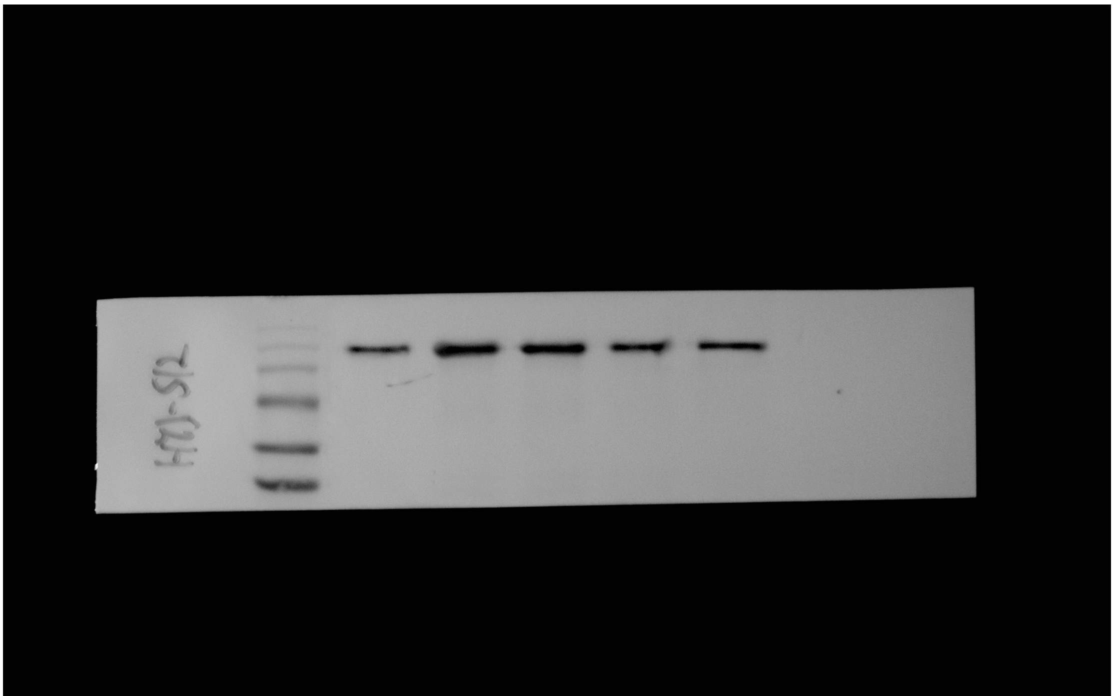

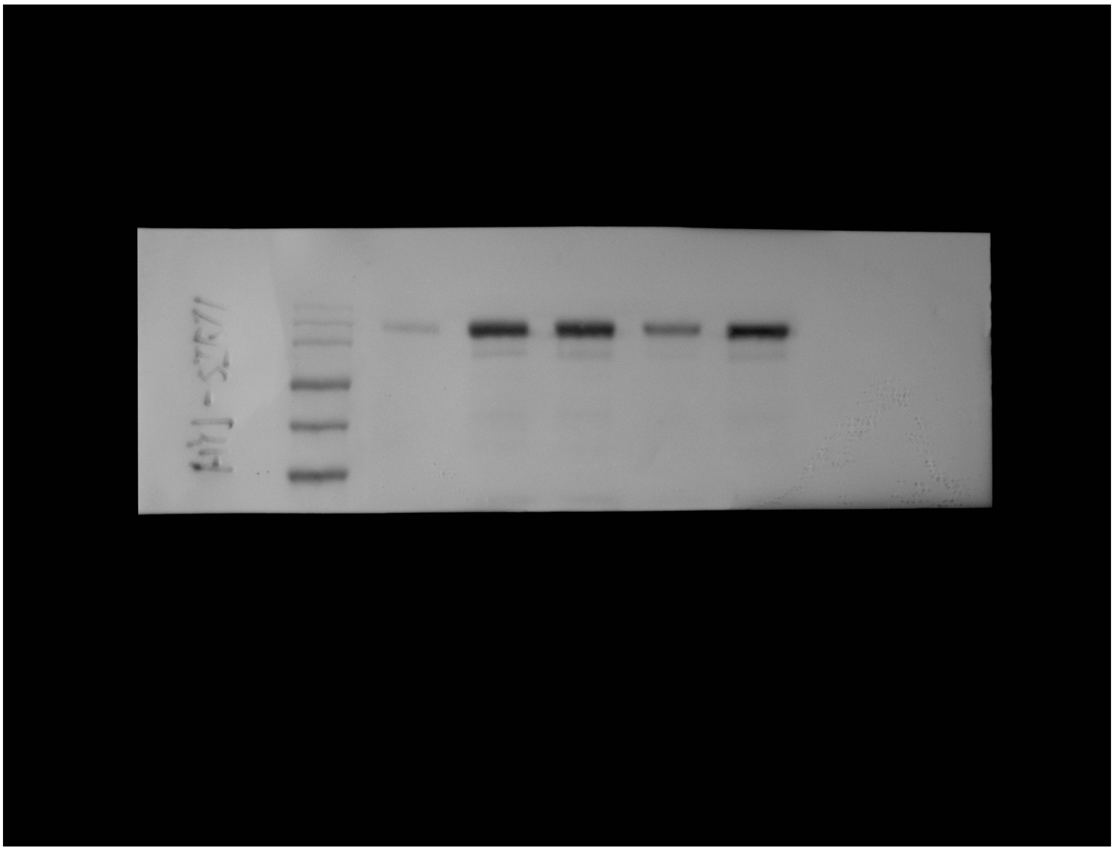

β-actin

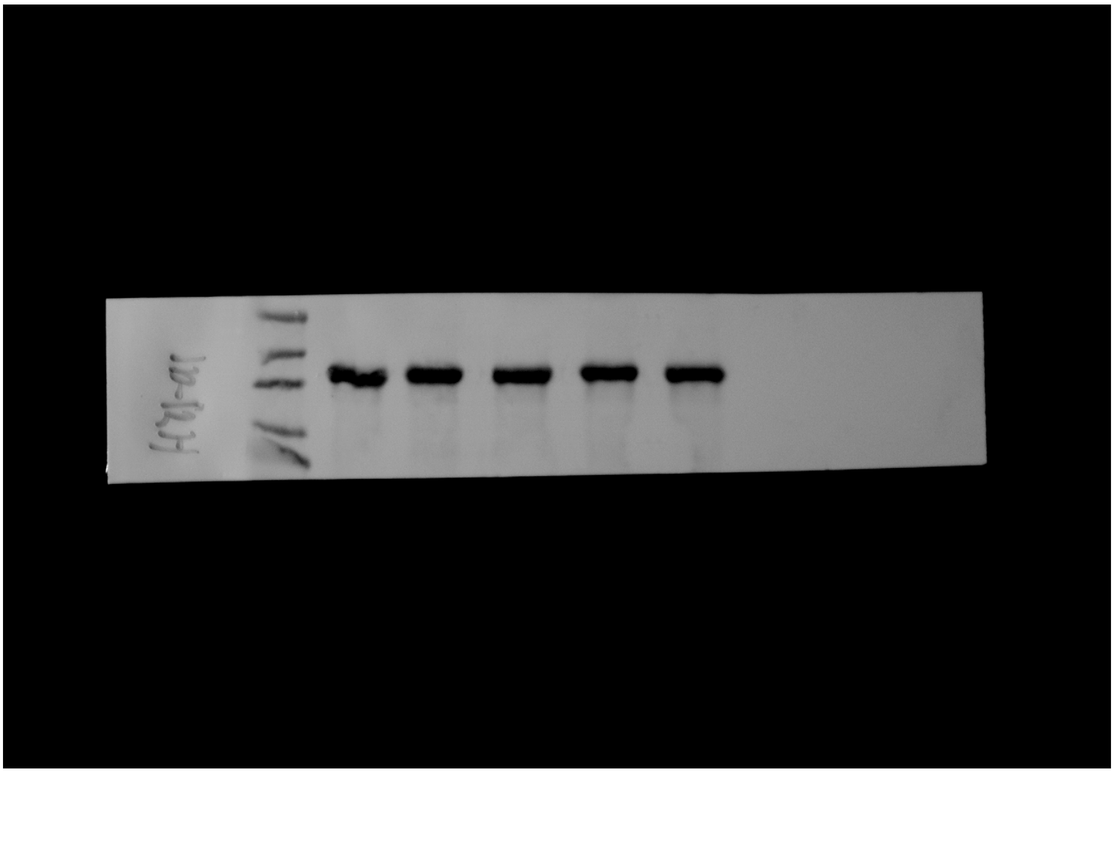

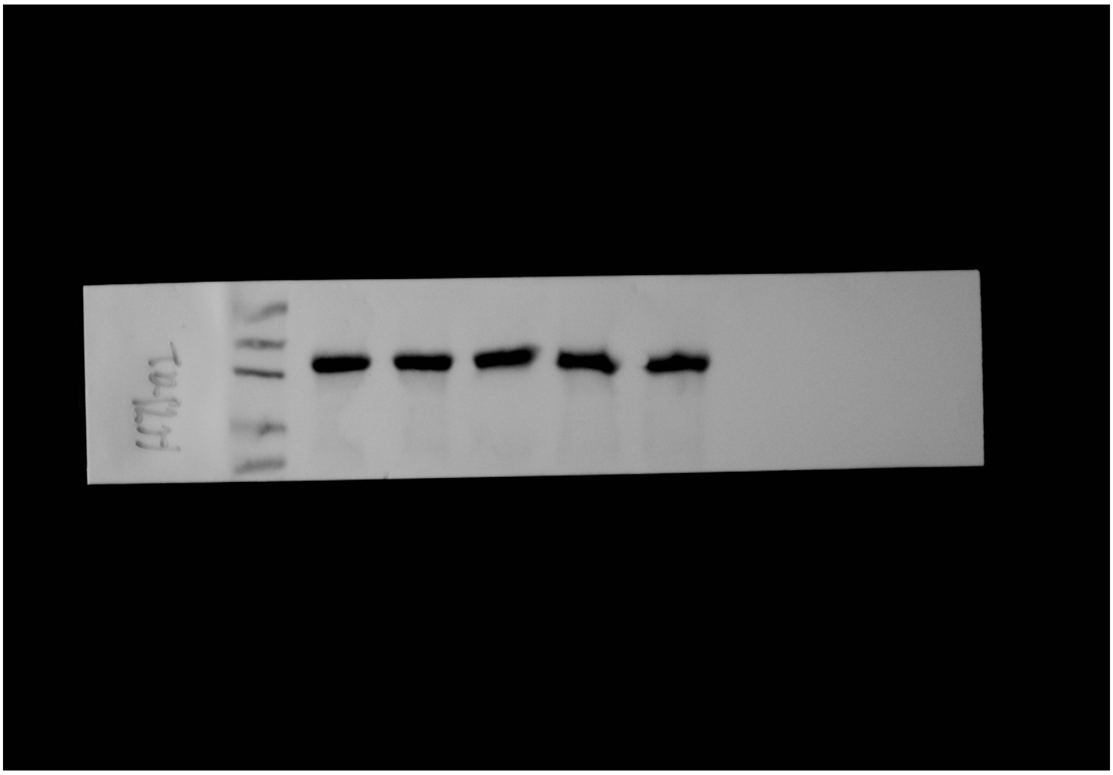

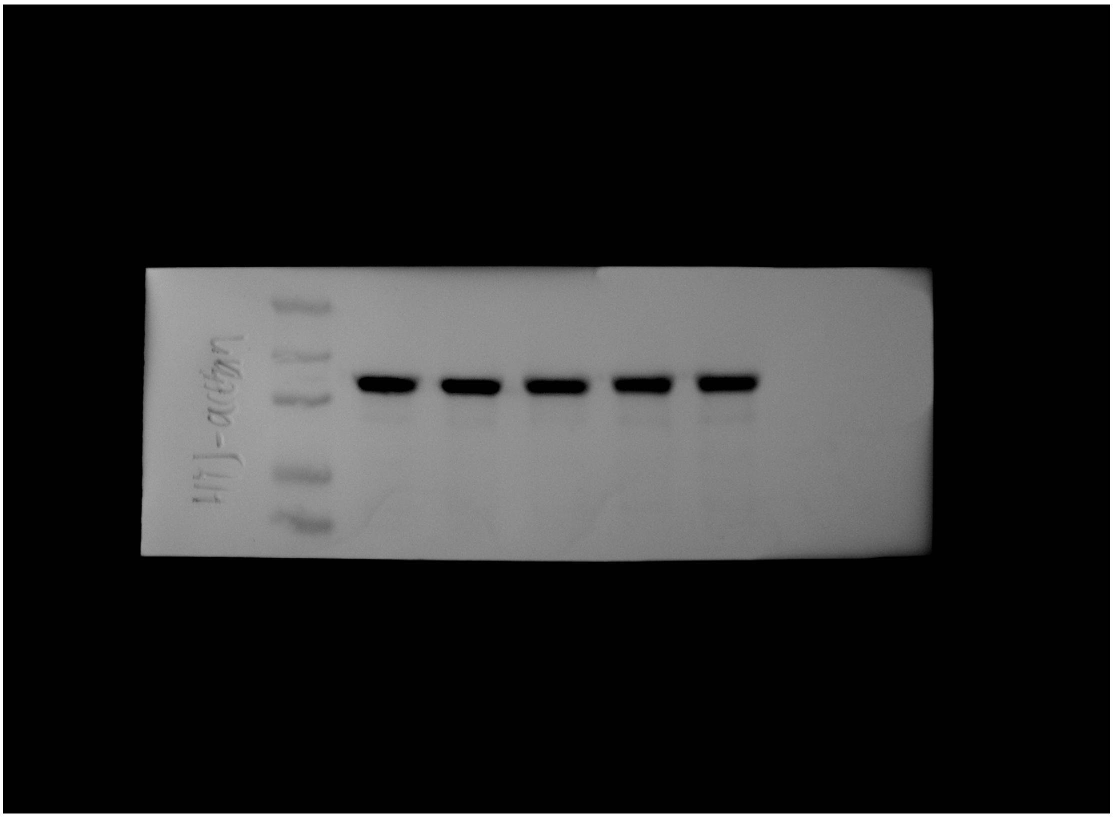

Figure 6D

GCLC

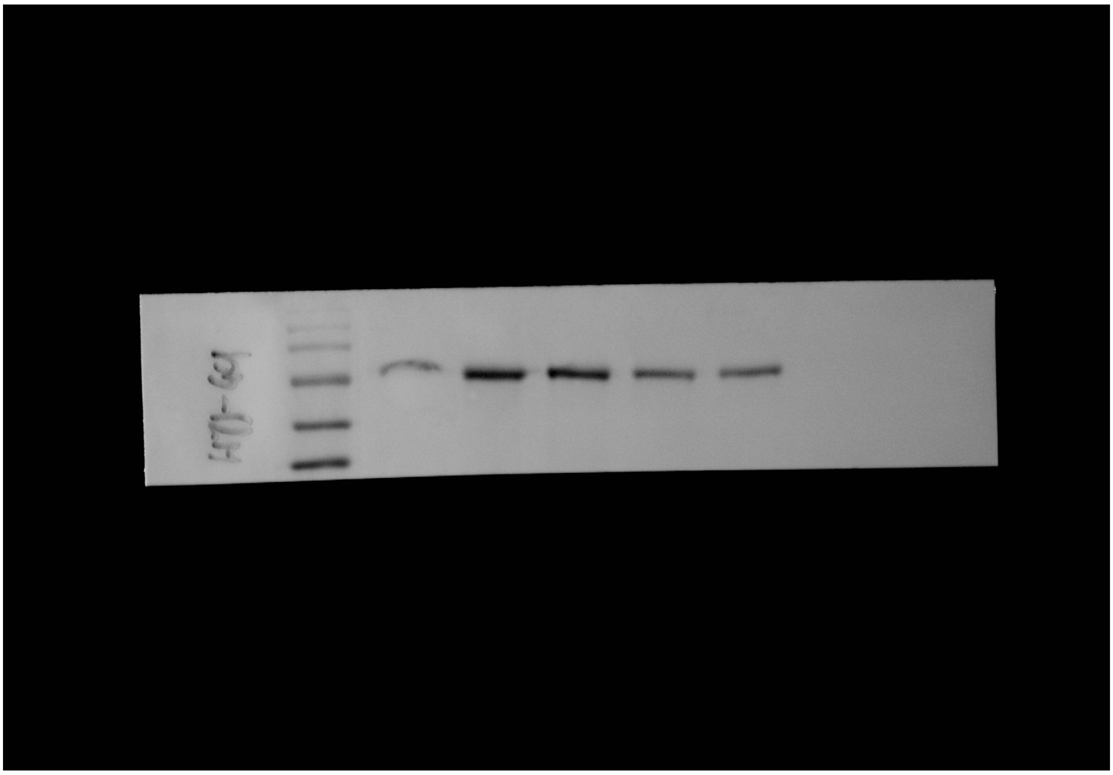

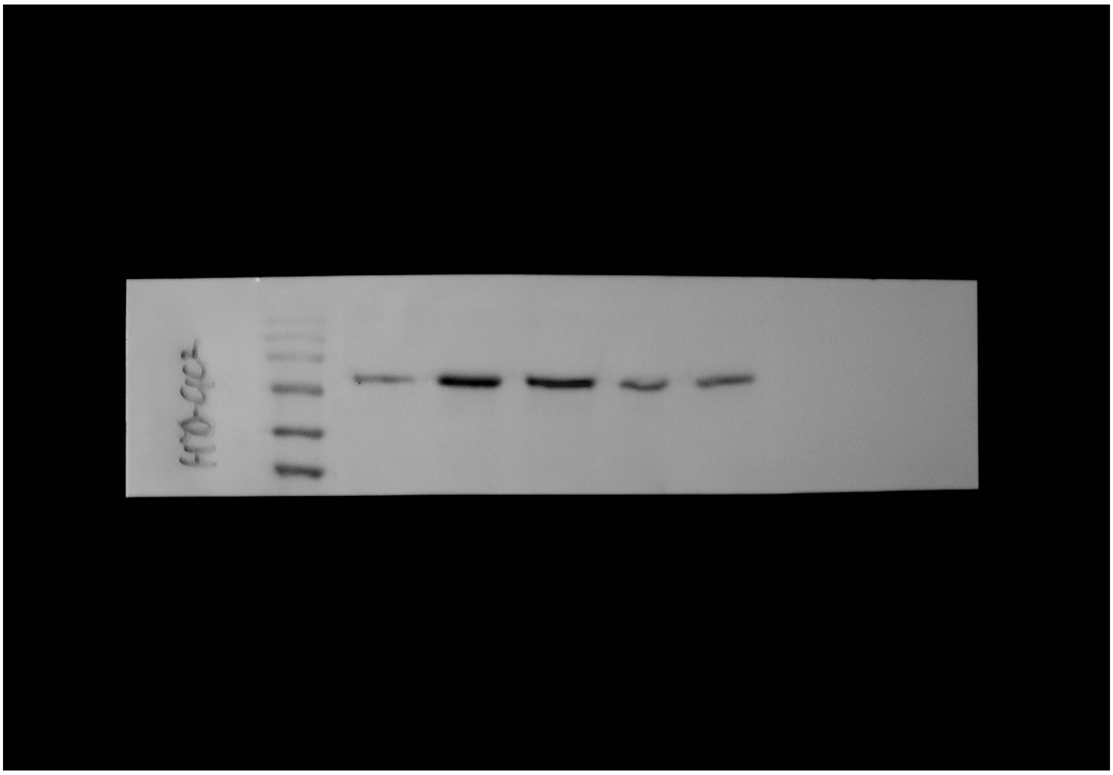

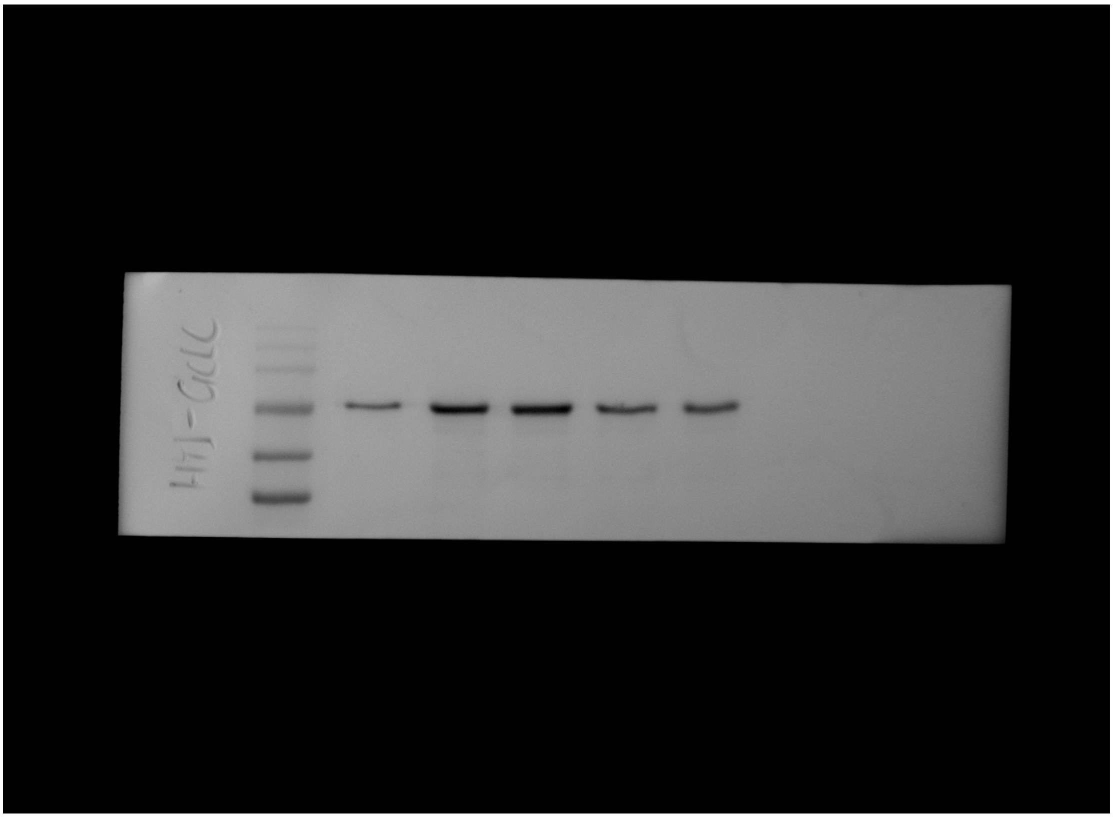

GCLM

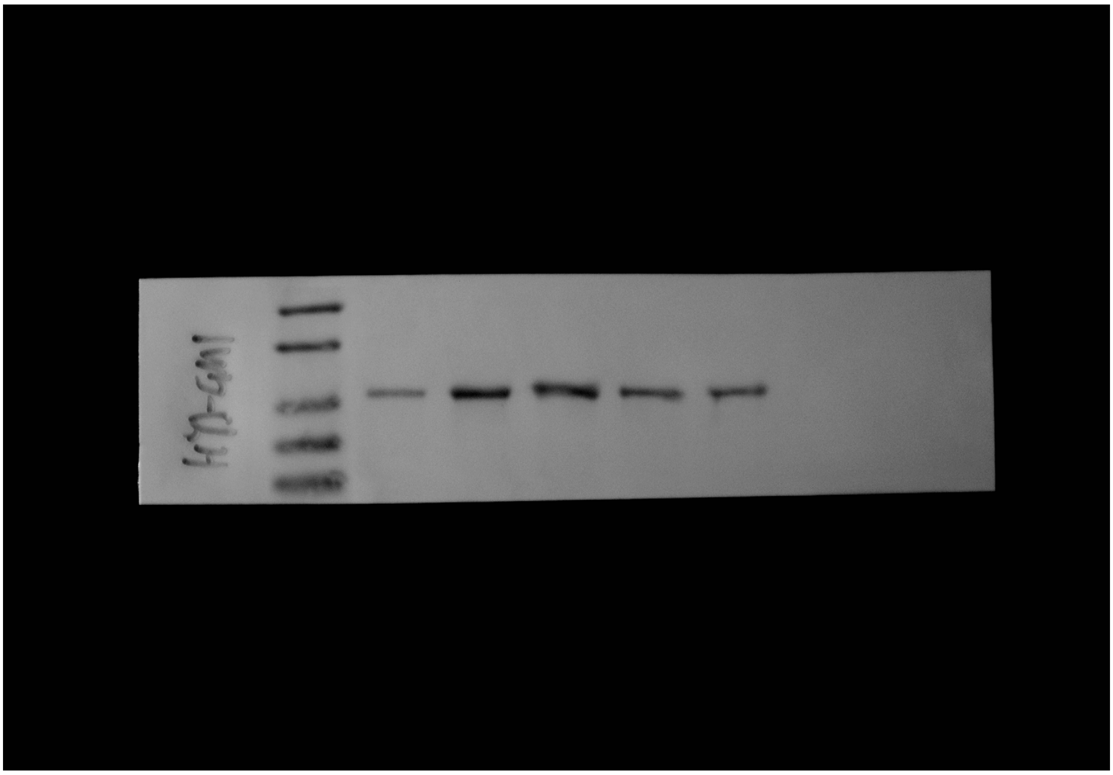

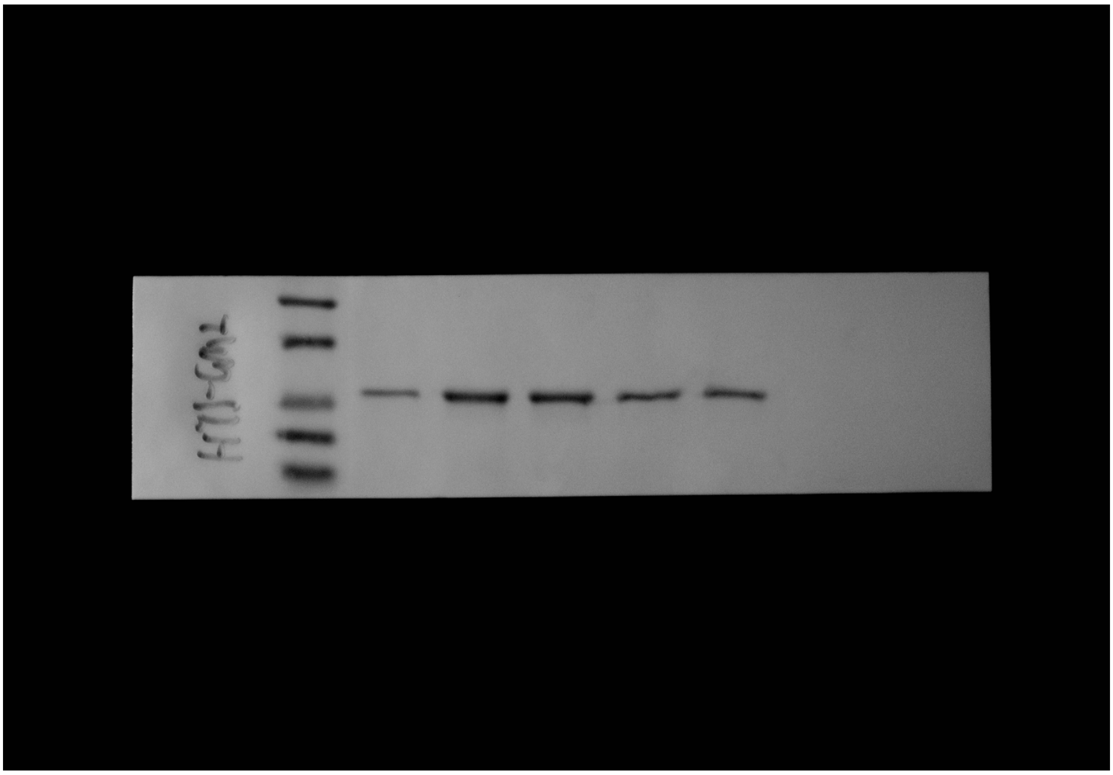

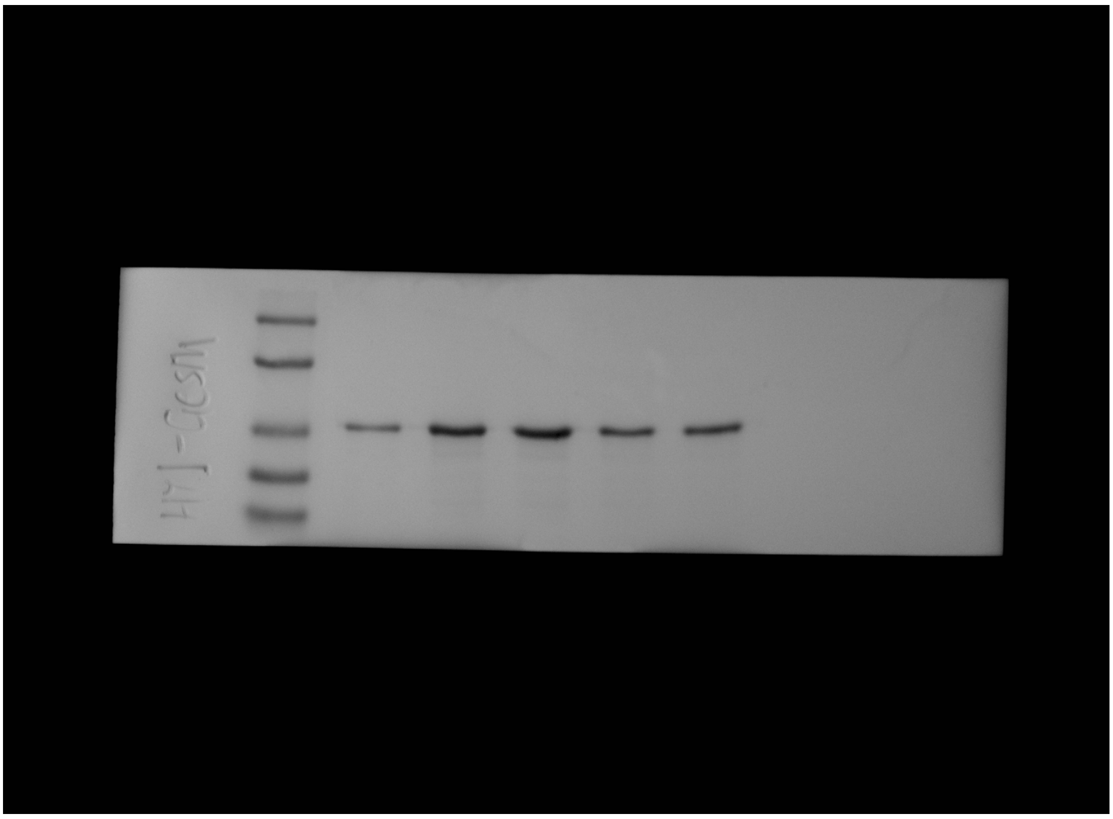

NQO1

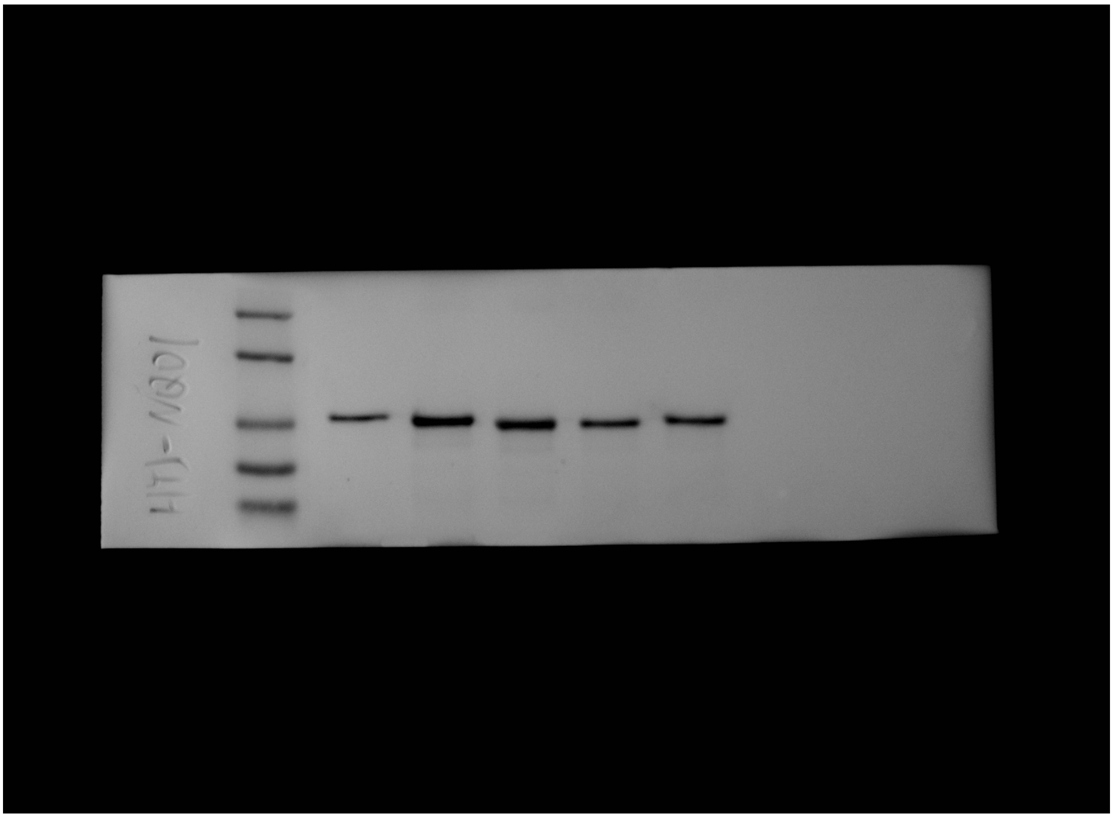

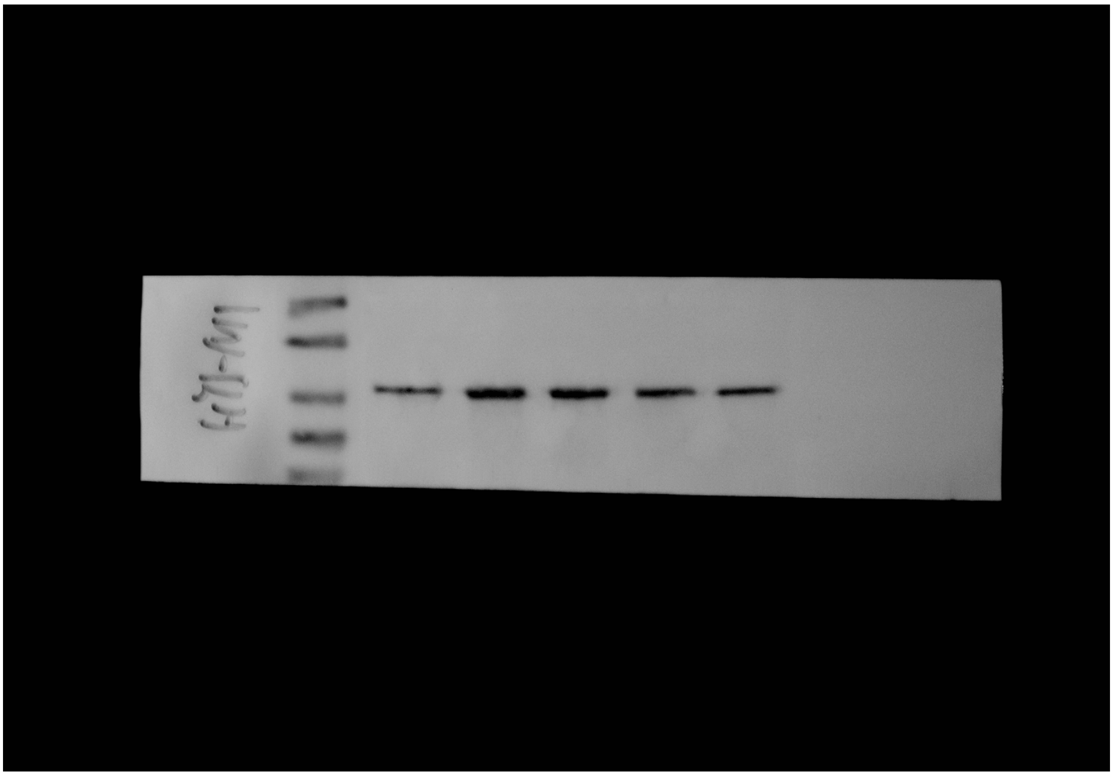

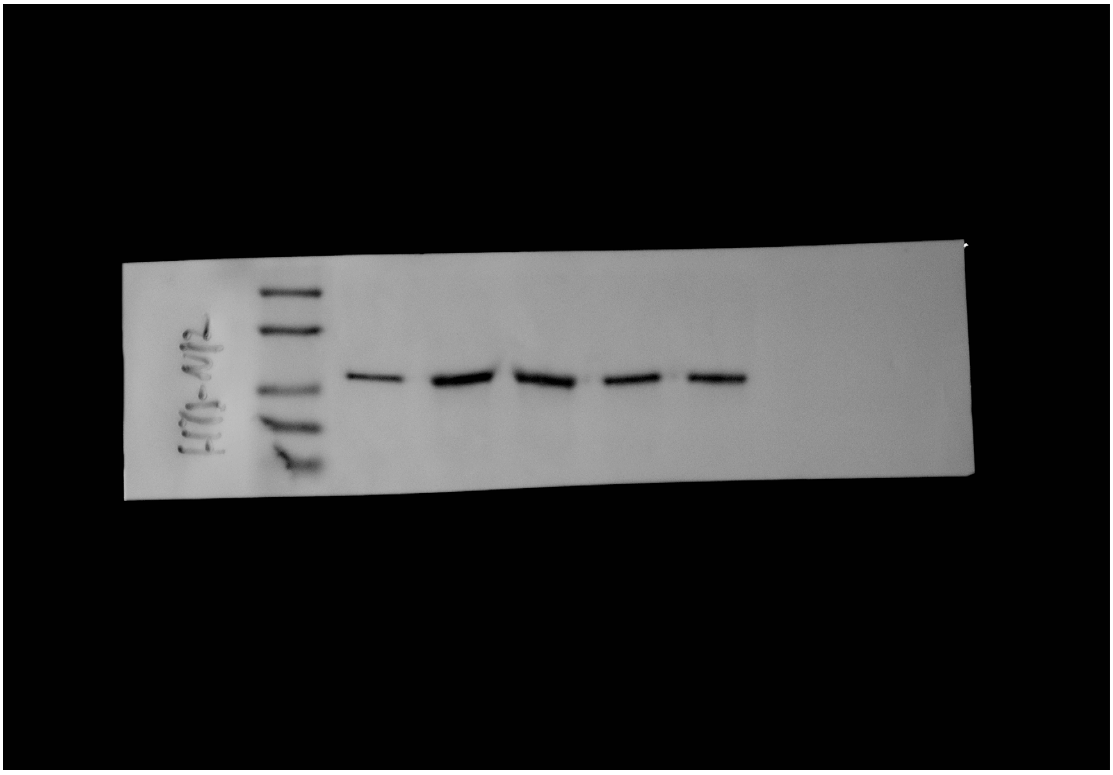

β-actin

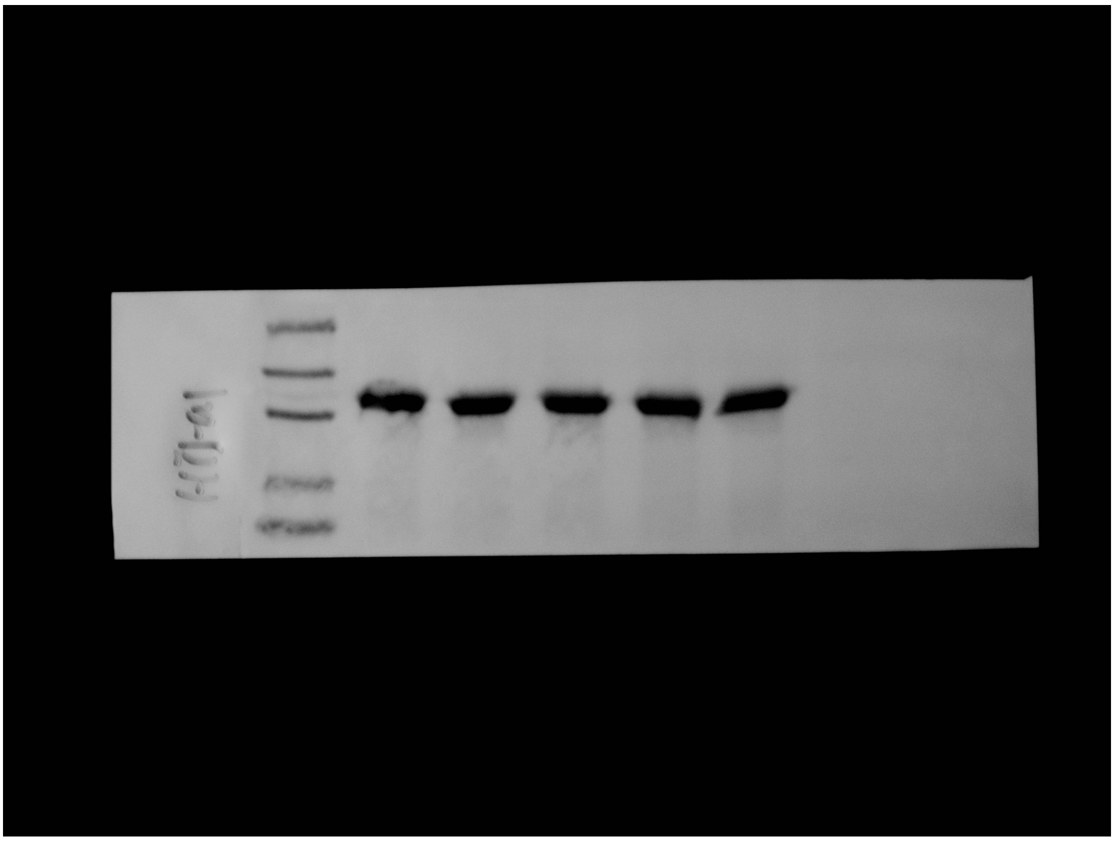

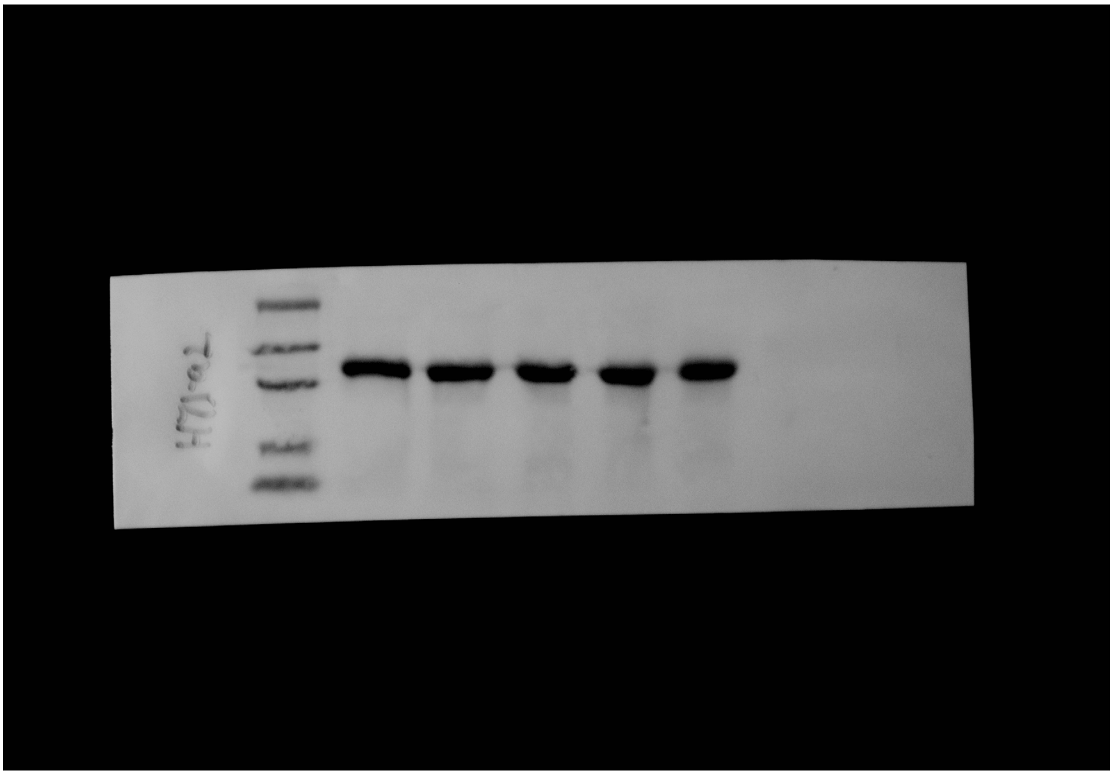

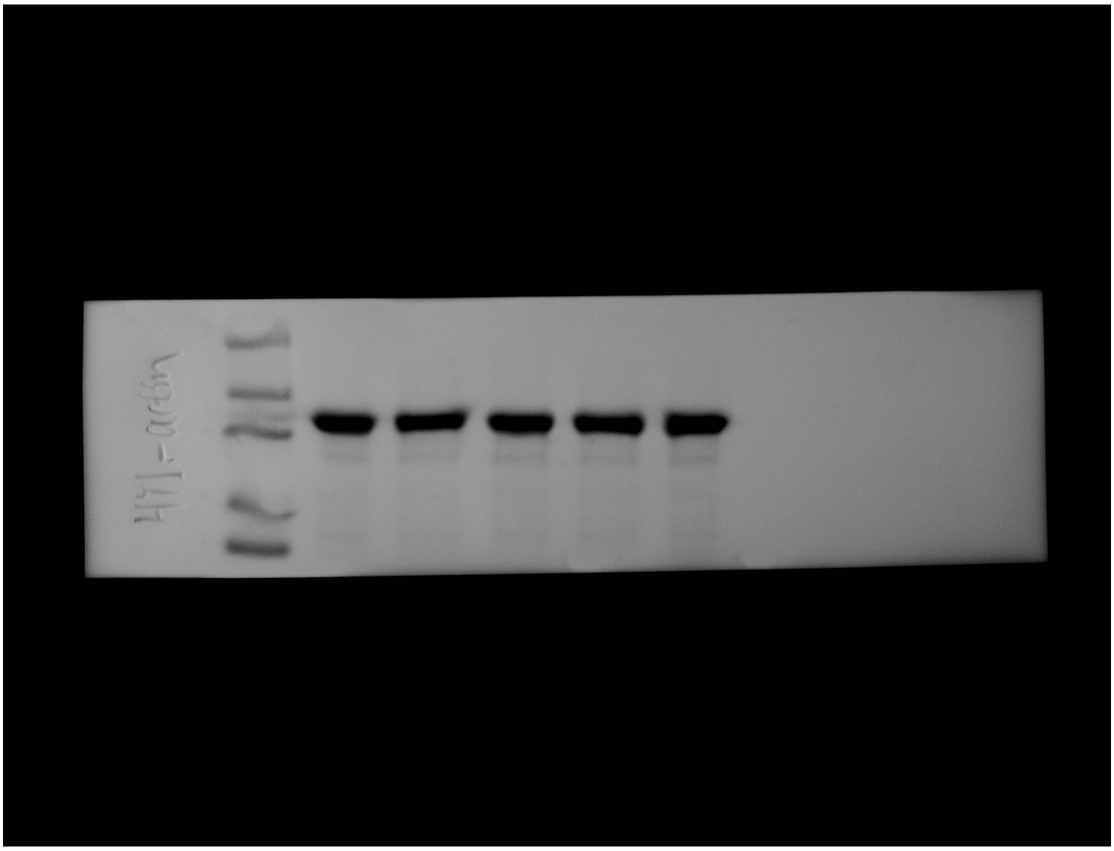

Figure 6F

E2F1

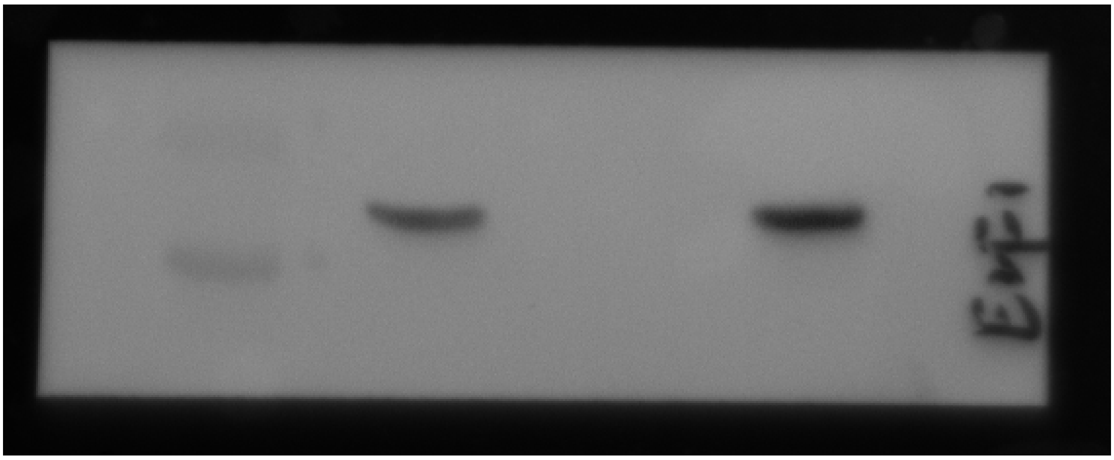

SIRT1

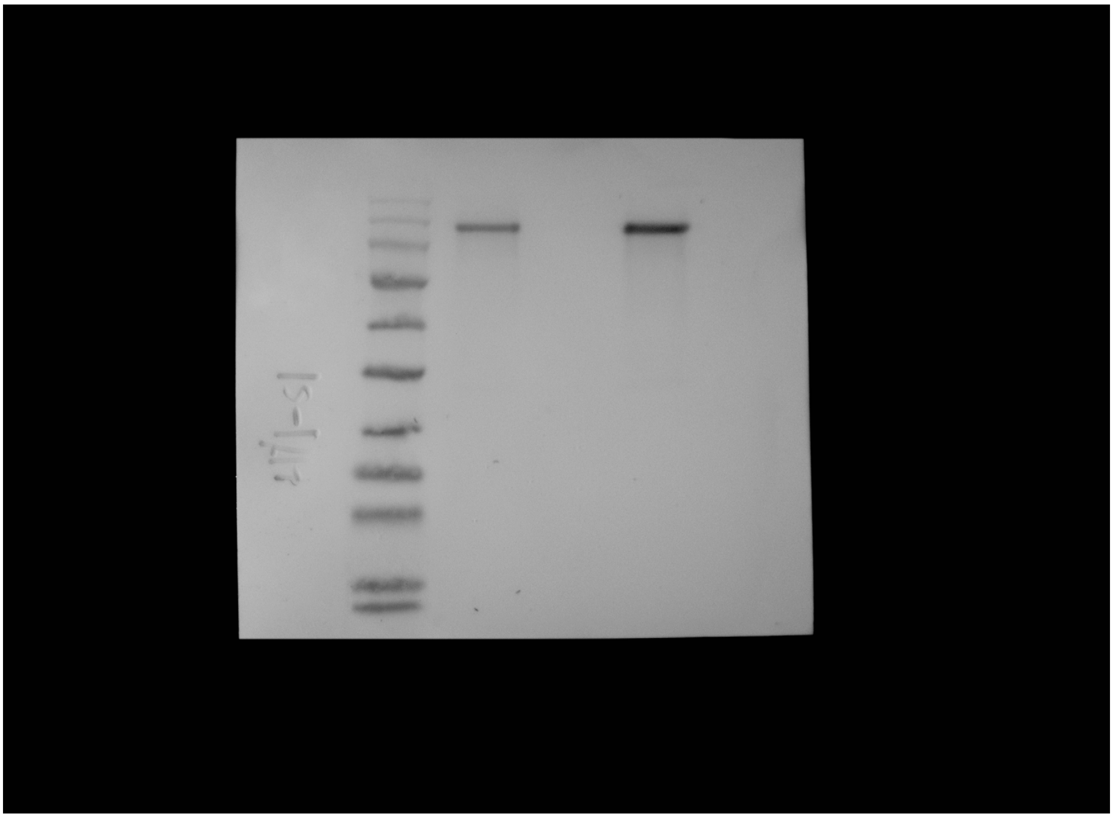

Figure 7B

BMAL1

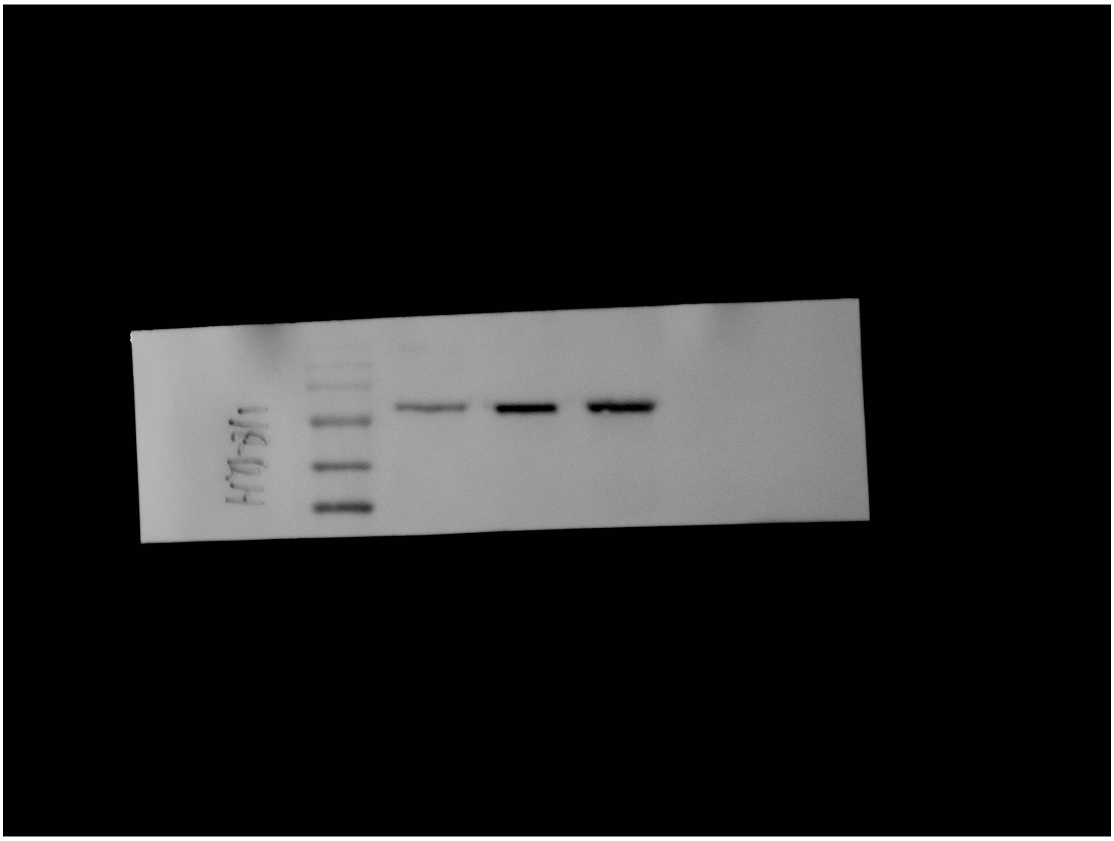

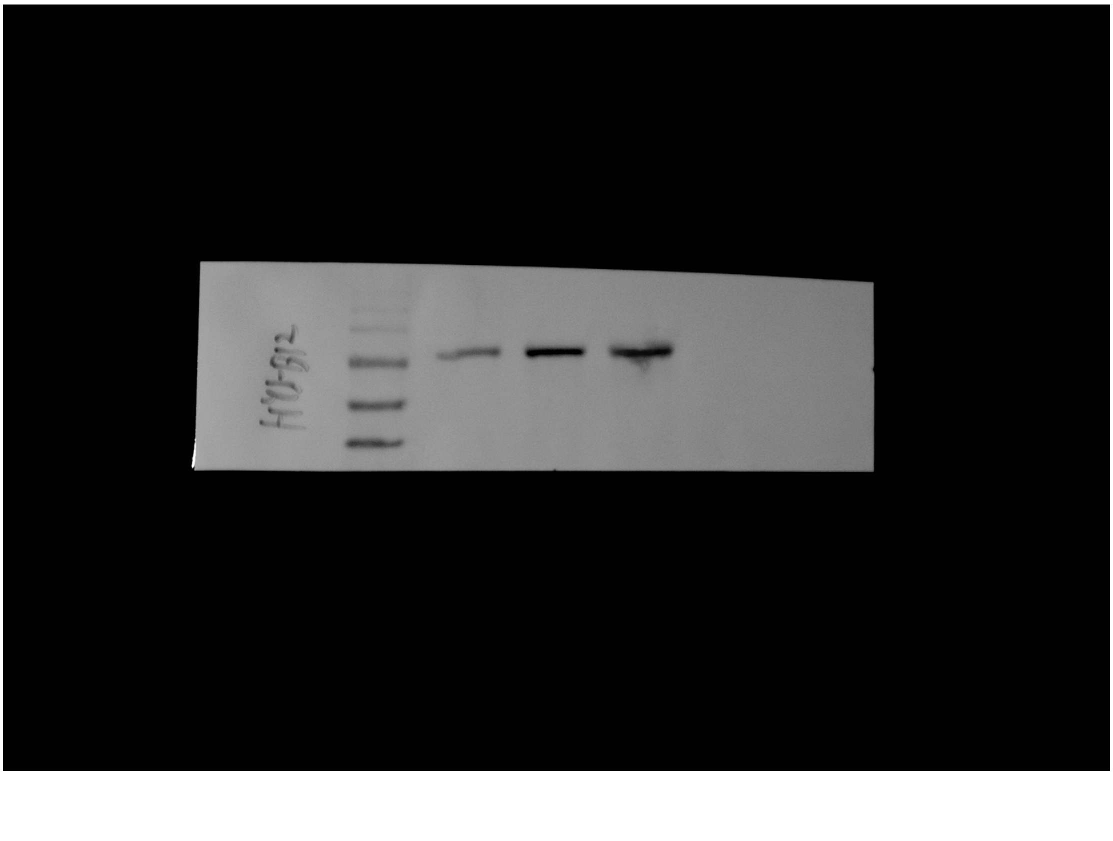

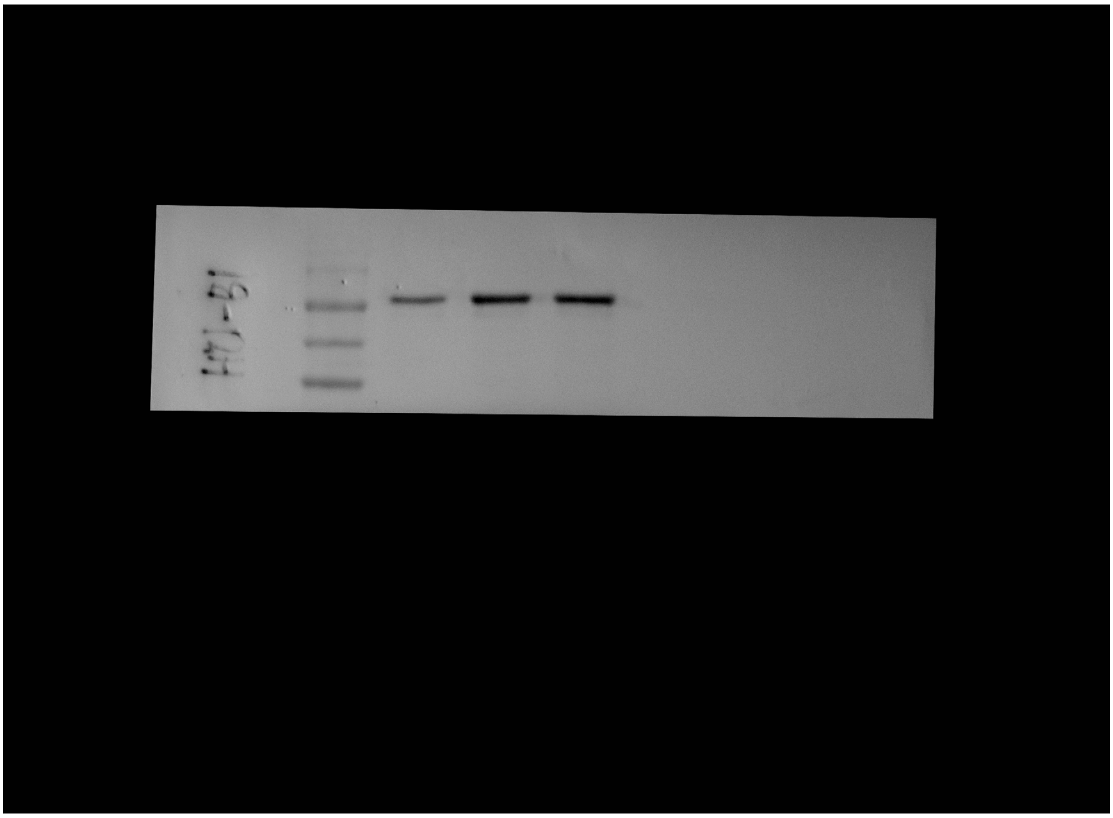

E2F1

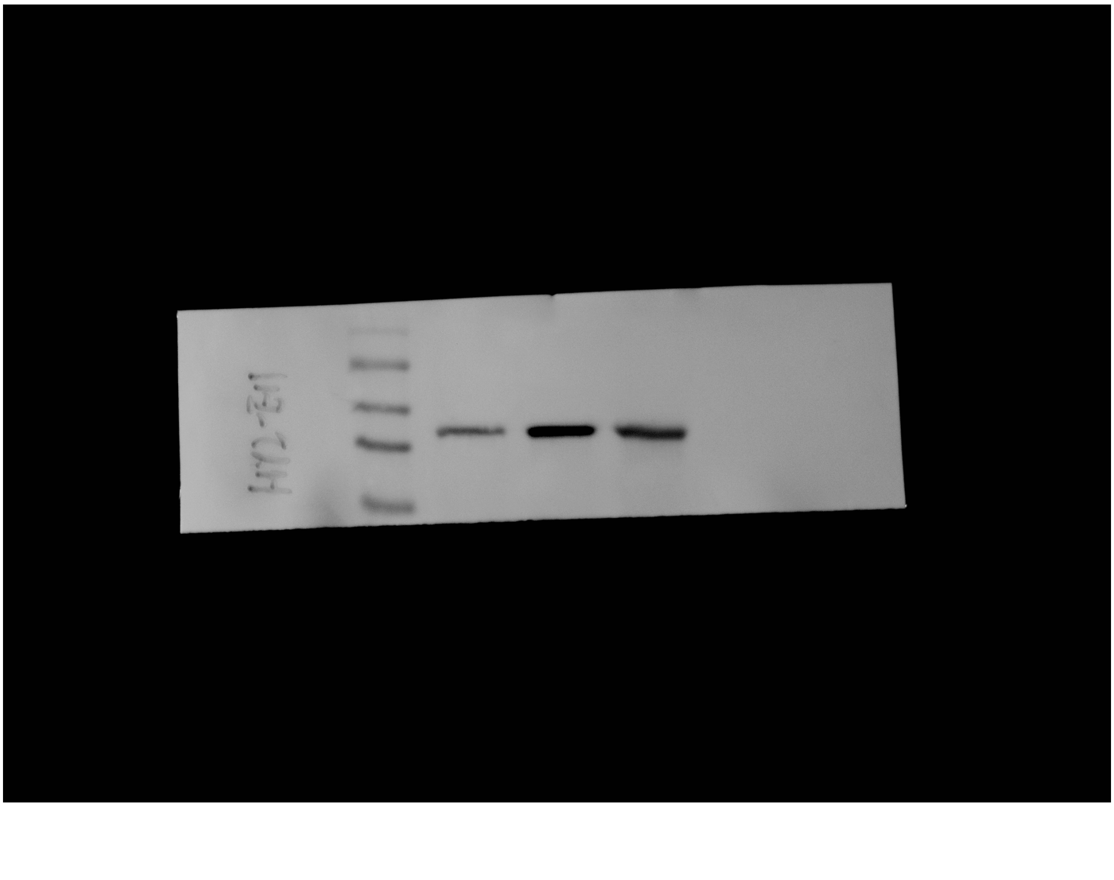

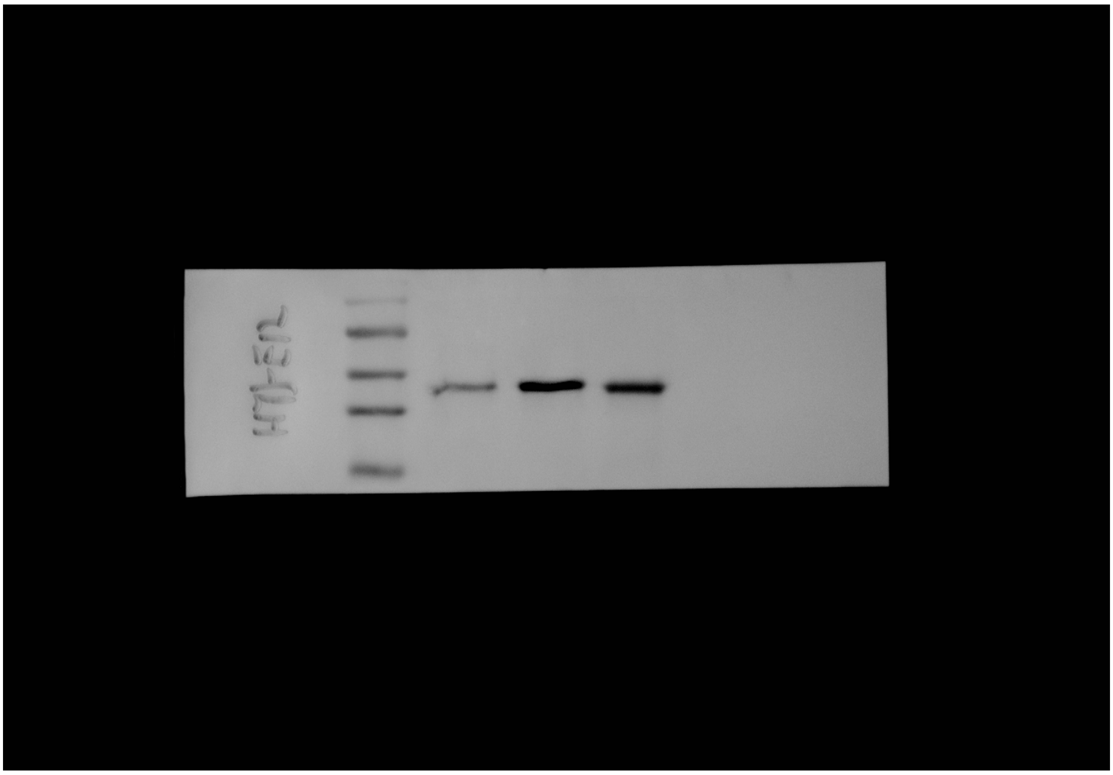

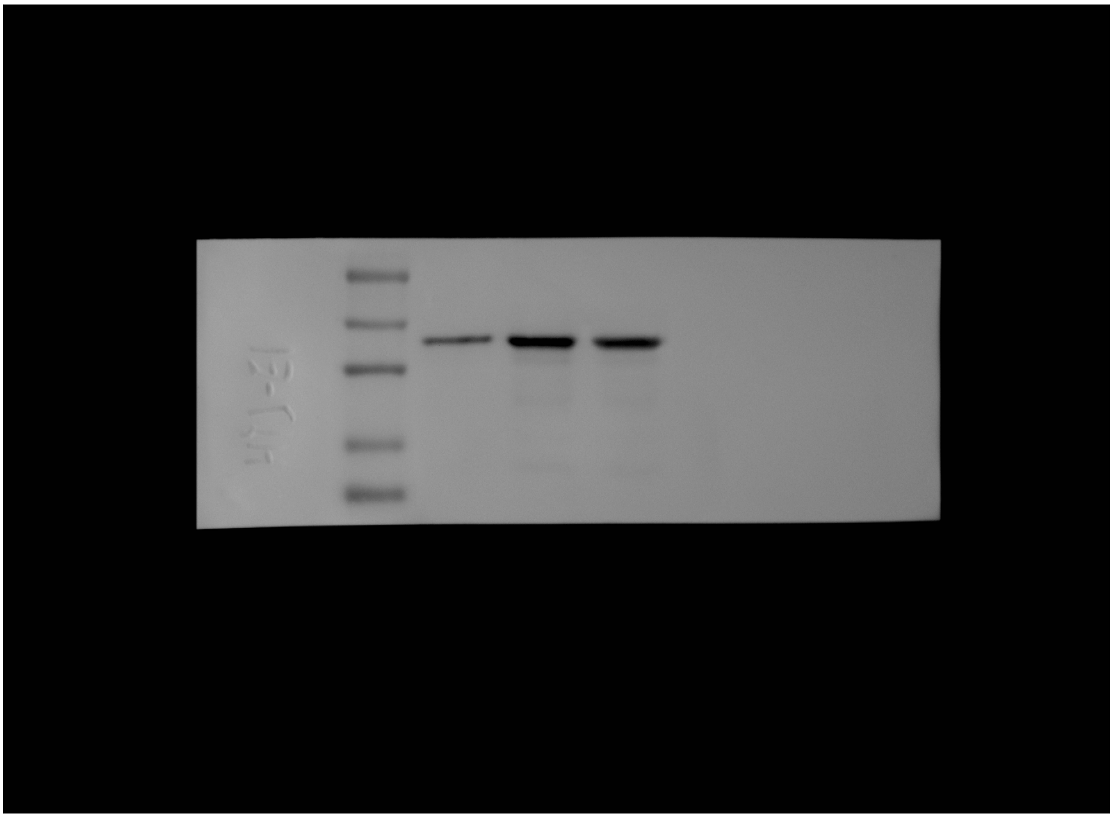

SIRT1

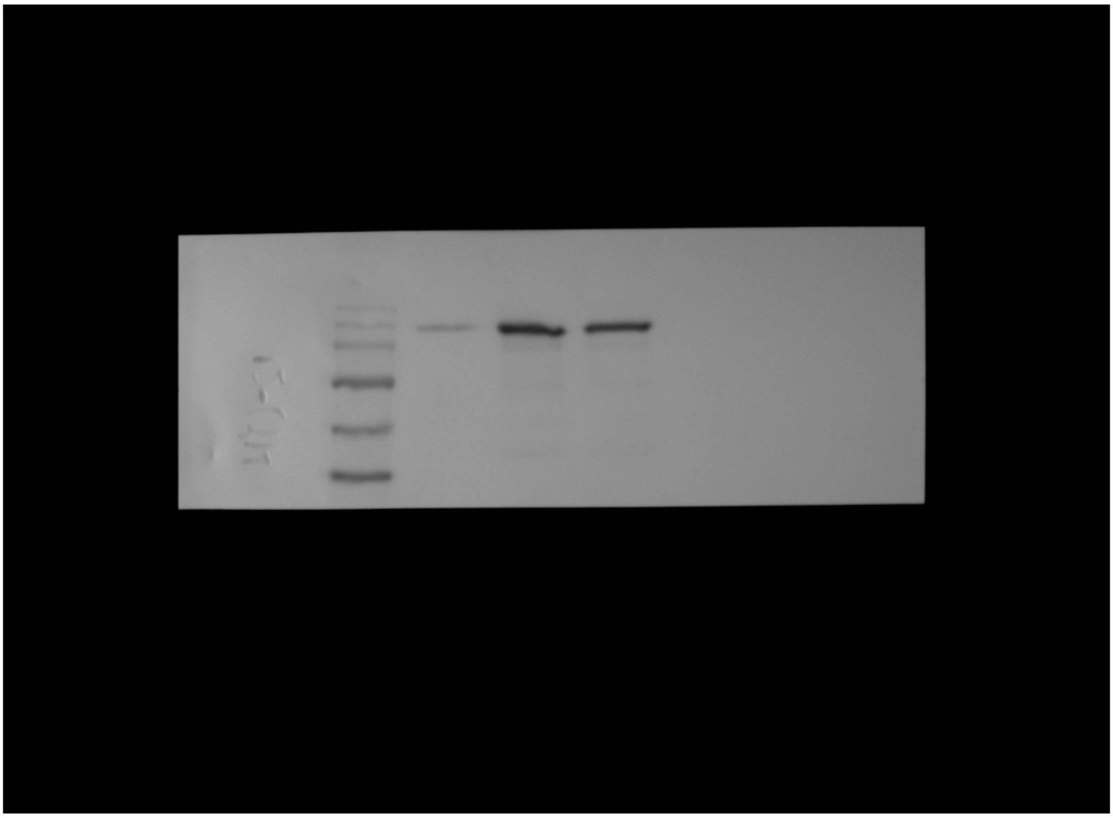

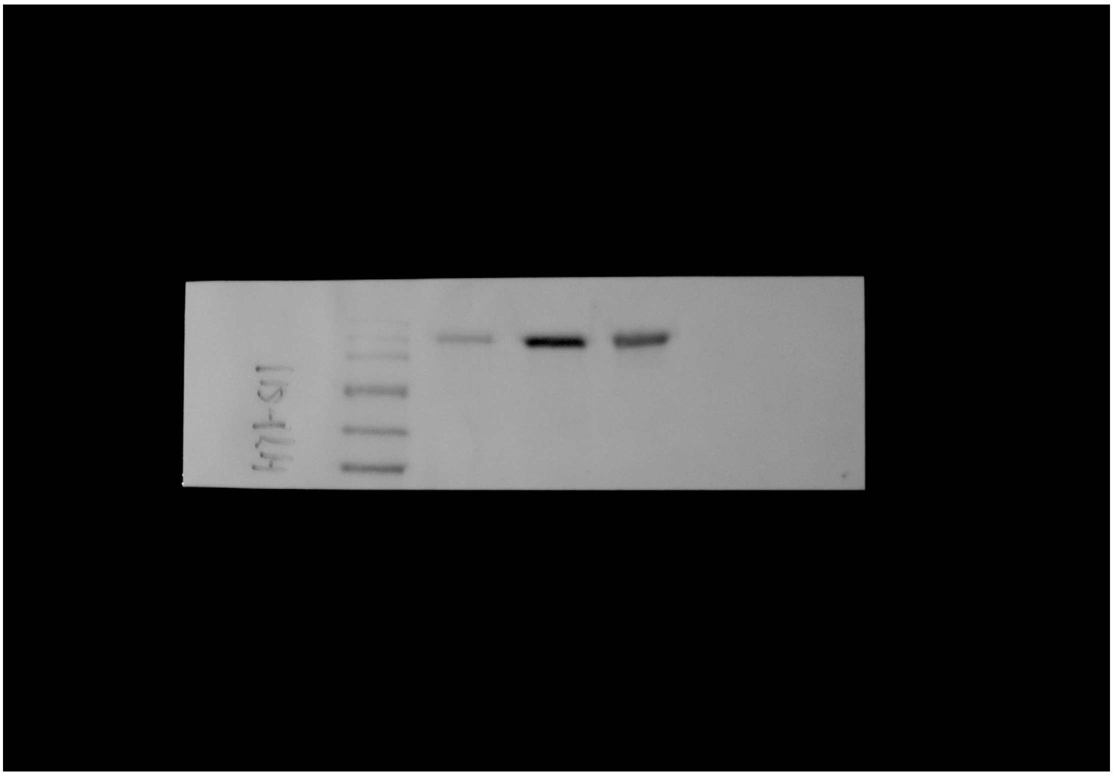

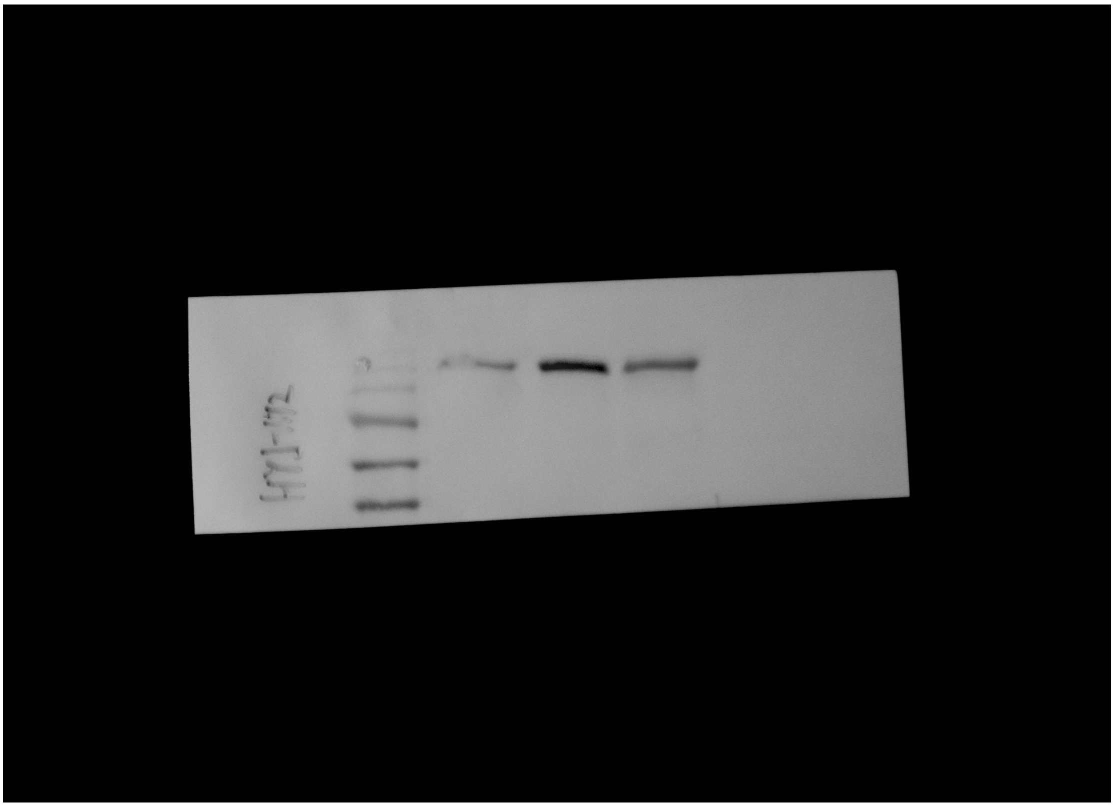

β-actin

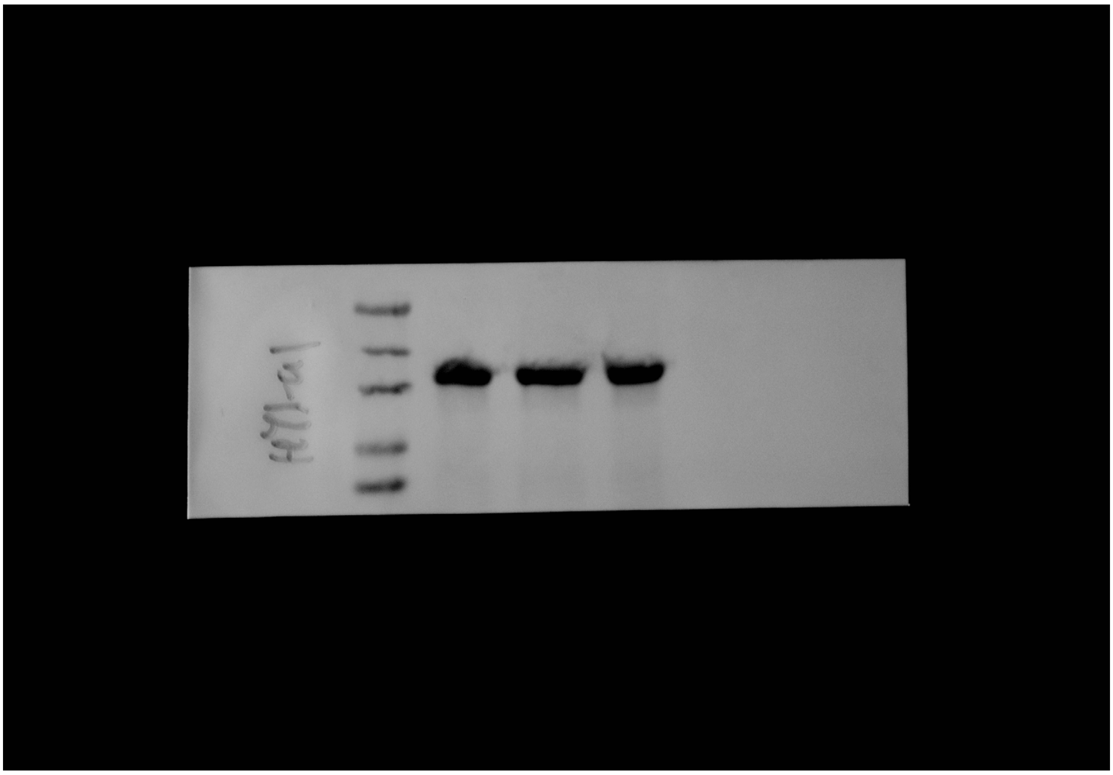

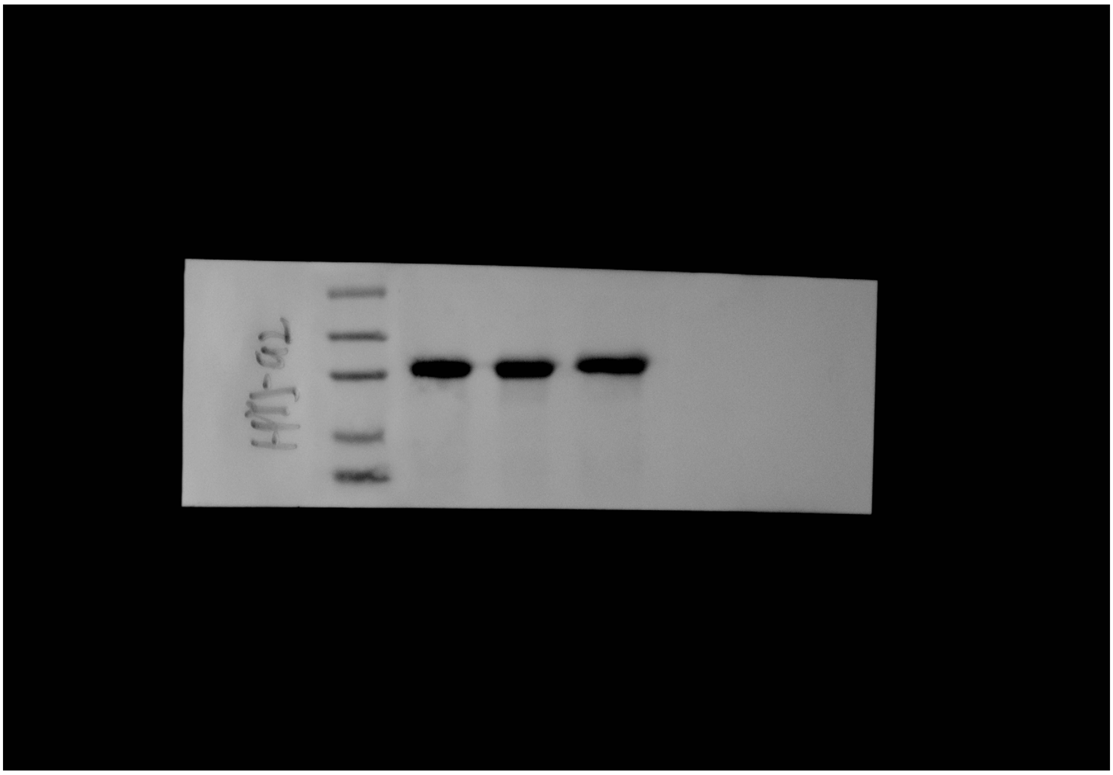

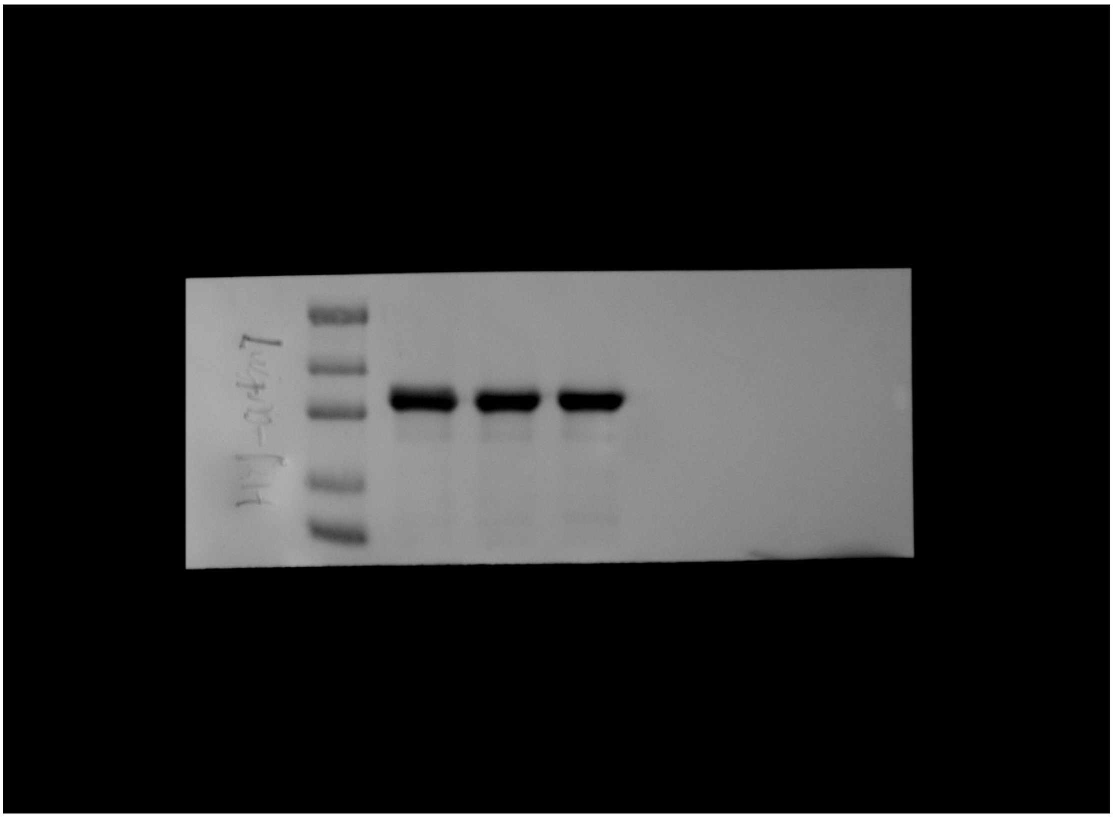

Supplement: Supplementary file 1 — Supplementary Figure 1 [file 41420_2025_2738_MOESM1_ESM.pdf]
